# Artemisinin resistance in *Plasmodium falciparum *is associated with an altered temporal pattern of transcription

**DOI:** 10.1186/1471-2164-12-391

**Published:** 2011-08-03

**Authors:** Sachel Mok, Mallika Imwong, Margaret J Mackinnon, Joan Sim, Ramya Ramadoss, Poravuth Yi, Mayfong Mayxay, Kesinee Chotivanich, Kek-Yee Liong, Bruce Russell, Duong Socheat, Paul N Newton, Nicholas PJ Day, Nicholas J White, Peter R Preiser, François Nosten, Arjen M Dondorp, Zbynek Bozdech

**Affiliations:** 1 School of Biological Sciences, Nanyang Technological University, Singapore; 2 Department of Molecular Tropical Medicine and Genetics, Faculty of Tropical Medicine, Mahidol University, Thailand; 3 Mahidol-Oxford Research Unit, Faculty of Tropical Medicine, Mahidol University, Thailand; 4 KEMRI-Wellcome Trust Research Programme, Kilifi, Kenya; 5 Wellcome Trust-Mahosot Hospital-Oxford University Tropical Medicine Research Collaboration, Mahosot Hospital, Vientiane, Lao People's Democratic Republic; 6 Faculty of Postgraduate Studies and Research, University of Health Sciences, Vientiane, Lao People's Democratic Republic; 7 Singapore Immunology Network, Biopolis, Agency for Science Technology and Research (ASTAR), Singapore; 8 Centre for Clinical Vaccinology and Tropical Medicine, Churchill Hospital, Oxford, UK; 9 The National Center for Parasitology, Entomology, and Malaria Control, Phnom Penh, Cambodia; 10 Shoklo Malaria Research Unit, Mae Sot, Thailand

**Keywords:** *Plasmodium falciparum, in vivo *artemisinin-resistance, field isolates, comparative genomics, comparative transcriptomics

## Abstract

**Background:**

Artemisinin resistance in *Plasmodium falciparum *malaria has emerged in Western Cambodia. This is a major threat to global plans to control and eliminate malaria as the artemisinins are a key component of antimalarial treatment throughout the world. To identify key features associated with the delayed parasite clearance phenotype, we employed DNA microarrays to profile the physiological gene expression pattern of the resistant isolates.

**Results:**

In the ring and trophozoite stages, we observed reduced expression of many basic metabolic and cellular pathways which suggests a slower growth and maturation of these parasites during the first half of the asexual intraerythrocytic developmental cycle (IDC). In the schizont stage, there is an increased expression of essentially all functionalities associated with protein metabolism which indicates the prolonged and thus increased capacity of protein synthesis during the second half of the resistant parasite IDC. This modulation of the *P. falciparum *intraerythrocytic transcriptome may result from differential expression of regulatory proteins such as transcription factors or chromatin remodeling associated proteins. In addition, there is a unique and uniform copy number variation pattern in the Cambodian parasites which may represent an underlying genetic background that contributes to the resistance phenotype.

**Conclusions:**

The decreased metabolic activities in the ring stages are consistent with previous suggestions of higher resilience of the early developmental stages to artemisinin. Moreover, the increased capacity of protein synthesis and protein turnover in the schizont stage may contribute to artemisinin resistance by counteracting the protein damage caused by the oxidative stress and/or protein alkylation effect of this drug. This study reports the first global transcriptional survey of artemisinin resistant parasites and provides insight to the complexities of the molecular basis of pathogens with drug resistance phenotypes *in vivo*.

## Background

Artemisinin combination therapy (ACT) is recommended by the World Health Organization as the first-line treatment for falciparum malaria in all endemic regions [1,2]. The excellent effectiveness and tolerability of ACTs brought new enthusiasm into world-wide efforts to eliminate human malaria which until today accounts for 243 million cases of infection and 863,000 deaths per annum [3]. The core components of ACTs - artemisinin and its derivatives, provide an important alternative to quinoline and antifolate-based compounds. Resistance to these older compounds that emerged on the Thai-Cambodian border and subsequently spread across the world has severely compromised their use and contributed to a dramatic rise in malaria morbidity prior to introduction of the ACTs in the late 1990's [4-7]. Learning from past mistakes, much effort is being invested in proper management of ACTs in order to sustain their efficacy and prevent the spread of resistance [1].

In spite of these efforts, there have been sporadic reports of artemisinin resistance *in-vivo *and *in-vitro *for many years (from Yunnan Province, Southwest China [8], Vietnam [9] and French Guiana [10]). Although the biological and clinical significance of these reports were uncertain [11], these early warning signs suggested a possibility of emergence of malaria parasites resistant to artemisinin [12-14]. Recently, unequivocal evidence of reduced artemisinin susceptibility from Western Cambodia has been reported [15]. Curiously, this was also the epicenter of chloroquine and sulfadoxine-pyrimethamine resistance. Dondorp et al. (2009) documented markedly prolonged parasite clearance times (median PCT 84 hours (interquartile range 60 to 96 hours) in Pailin, Western Cambodia. This compares with a median PCT of 48 hours (36 to 66 hours) on the Western border of Thailand [15]. Since this study, reports of delayed parasite clearance have emerged in other parts of the region, including the Thai-Myanmar border [13]. Although it has yet to be established whether artemisinin resistance has spread westward, the possibility of the spread of resistant parasites through Asia to Africa would be disastrous.

The mechanism of artemisinin resistance is unknown. The resistant phenotype detected in Western Cambodia does not associate with any polymorphisms in the established drug resistance markers [15]. *In vitro *susceptibility testing of parasites which are cleared abnormally slowly *in vivo*, showed essentially no shift in IC_50 _(50% inhibition concentration) values *in vitro *[15]. This apparent discrepancy between the experimental and the clinical data may be explained by the reduced susceptibility of *Plasmodium *parasites at only the ring stage (first third) of its 48 hours intraerythrocytic developmental cycle (IDC) [15,16]. Another phenomenon that has been suggested to explain artemisinin resistance is an increased propensity for these parasites to form "dormant" (or quiescent) rings under artemisinin exposure [17,18]. However, this process, suggested by *in vitro *studies, is unlikely to explain the slow first order decline in parasitemia with time that was observed in Western Cambodian patients treated with artemisinin-based drugs [19]. The reduced artemisinin susceptibility phenotype of the resistant malaria parasites exhibits a heritable pattern suggesting that it has a genetic basis [20]. Identifying the genetic determinants will be crucial for understanding the molecular basis of artemisinin resistance and will also provide an important molecular tool for epidemiological surveys.

Here we carry out genome-wide gene expression analyses in order to identify key elements of a transcriptional profile underlining artemisinin resistance. We show that the *P. falciparum *parasites with slow clearance after artemisinin treatment exhibit reduced expression levels of generic metabolic (e.g. glycolysis, nucleotide metabolism) or cellular (e.g. DNA replication) pathways in the ring and trophozoite stages but strong increased expression of essentially all functionalities associated with protein synthesis, folding and trafficking in the schizont stages. This specific "tune-up" of the transcriptional pattern in these resistant *P. falciparum *isolates is associated with altered expression of a number of genes involved in cell cycle regulation, transcription regulation, chromatin remodeling as well as intracellular signaling. Altogether these results provide the first set of testable genetic markers associated with artemisinin resistance.

## Results

### Gene expression associated with artemisinin resistance

The main purpose of these studies was to characterize the transcriptional profile associated with artemisinin resistance in field isolates of *P. falciparum *[15]. For this, we conducted DNA microarray analyses of parasites collected from patients in Pailin, Western Cambodia which had slow parasite clearance (detectable parasitemia 78-96 hours following administration of ACTs; isolates CP025, CP037 and CP040; Additional file 1). For comparison we analyzed transcriptomes of additional Southeast Asian isolates collected from Xepon, Savannakhet Province, Laos (isolates BMT061, BMT076, BMT077, XPN003), from Mae Sot, Thailand (isolates NHP2094, NHP4459, NHP4460), and one additional isolate from Pailin (CP022) with normal clearance (~54 hour). At the time of collection, all isolates exhibited high synchronicity; ~100% parasites were at the ring stage (Additional file 2). Parasites were then cultured for up to 48 hours *in vitro *and total RNA was isolated from samples harvested at regular intervals of 2-8 hours.

**Figure 1:**
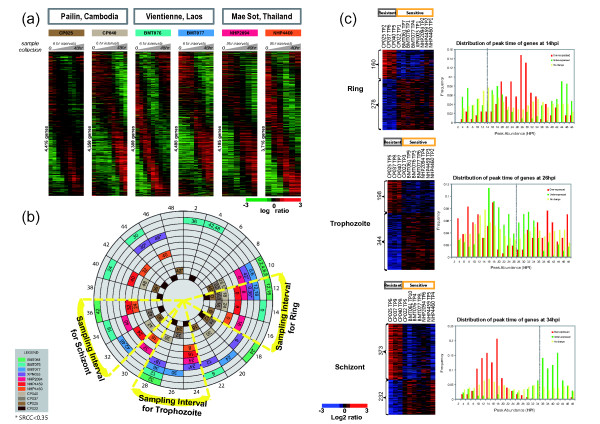
**Transcriptome analyses of the *ex vivo *cultured *P. falciparum *parasites**. (a) Transcriptomes of the *ex-vivo *IDC of 6 field isolates (representative set of total 11 transcriptomes generated in this study; Additional file 3) from the 3 geographical locations over 48 hour sampling time. The heat maps represent the mean-centered log_2 _microarray expression ratios for the *P. falciparum *genes that were ordered according to the phase calculated by the Fast Fourier Transformation for the reference *in vitro *IDC transcriptome. (b) Age estimate of the *ex vivo *culture time points for all 11 field isolates. Each colored box represents age estimate (hpi; shown by numbers outside the circle) of the isolate sample relative to the *in vitro *reference IDC transcriptome calculated as a best fit Correlation (Spearman rank, see materials and methods). Indicated within each box is the sampling collection time with respect to the first sampling time denoted by 0 h that correspond to the initial sample collection from the infected patients prior to culturing. Sampling times with * represent Spearman Rank correlation value less than 0.35 which indicates a deteriorating synchronicity in the later IDC time points and were excluded from the analysis. Time points included in 3 windows (yellow frame) corresponding to rings at 12-16 hpi, trophozoites at 24 to 28 hpi and schizonts at 32 to 36 hpi were selected for further analysis. (c) Clusters of genes with significant differential expression (p-value < 0.01) between the resistant (grey) and susceptible (orange) parasites at ring (14 hpi), trophozoite (26 hpi) and schizont (34 hpi) stages. Shown are the mean-centered expression log_2 _ratios for these genes ranked by the z-score based on the differential expression between the resistant and susceptible isolates. Graphs represent the frequency distribution of the peak abundance time in the IDC transcriptome for each group of genes: over-expressed (red bars), under-expressed (green bars) or no significant change in expression (yellow bars). The grey lines represent the middle of the time IDC interval used for the analysis (e.g. ring, (14 hpi), trophozoite (26 hpi) and schizonts (34 hpi).

Similar to previous experiments on *in vitro*-adapted isolates [21], these *ex vivo *samples displayed extensive stage specificity in transcriptional regulation during their 48-hour IDC (Additional file 3). However, in contrast to the *in vitro *conditions, the assembled *ex vivo *transcriptional profiles indicate considerable differences in the rates of stage progression (Figure 1a; Additional file 3). Using a Spearman-rank/Pearson correlation method, we identified a best fit of the "age" of each *ex vivo *experimental point based on peak correlation values using the previously generated *P. falciparum *IDC transcriptome *in vitro *[22]. Here we observed that while at the time of blood collection, all parasite populations correspond to the ring stages (10-16 hours post invasion, hpi), the subsequent IDC development was subjected to significant fluctuations (Figure 1b). 5 out of the 11 isolates (BMT061 and BMT077 from Laos, NHP2094 from Thailand and CP040 and CP037 from Cambodia) exhibited an initial developmental arrest in the ring stage for 16-24 hours post collection (Figure 1b). In spite of this initial arrest, these isolates reactivated their IDC progression and developed unidirectionally through trophozoite and schizont stages. The progress of the *ex vivo *cultured *P. falciparum *isolates through these later stages was also uneven. The most extreme example is the Lao isolate BMT077 that showed additional arrests in the trophozoite and early schizont stages at 30 to 48 hours post collection (Figure 1a and 1b). Overall, the developmental shifts in the IDC progression do not correlate with the site of collection, thus excluding the possibility of experimental bias due to culturing techniques in each field laboratory. These data support the previous observation of Lemiuex et al. (2008) who showed that the ages of 25 *P. falciparum *isolates cultured *ex vivo *for 48 hours fell into a large interval of 20-44 hpi [23]. In addition, the correlation coefficients between the mRNA profiles of field isolate time points and the *in vitro *control transcriptome are above 0.55 which demonstrates a good synchronicity of the *ex vivo *cultures (Additional file 4). Taken together, these data show that careful assessment of the parasite stage of development (age) at any experimental time point using a reference dataset is essential in *ex vivo *analyses of *Plasmodium *parasites.

To identify genes whose expression is associated with artemisinin resistance, we compared global transcription levels between the three artemisinin resistant isolates and the remaining sensitive isolates by analyzing steady state mRNA levels in 3 developmental stages; ring (12-16 hpi: hours post invasion), trophozoite (24-28 hpi) and schizont (32-36 hpi) (Figure 1b). For that, we included the experimental time points that show highest correlation value to the *in vitro *reference transcriptome [22] within these sampling intervals (Figure 1b). Overall, we identified 160 (3.9%), 198 (4.9%) and 373 (9.2%) genes that are up-regulated and 278 (6.9%), 344 (8.5%) and 232 (5.7%) genes that are down-regulated in the resistant parasites (p-value < 0.01) at the ring, trophozoite and schizont stages, respectively (Figure 1c, Additional file 5). Interestingly, the majority of the genes over-expressed in the ring stage of the resistant parasites correspond to trophozoite- specific genes (peak mRNA abundance in the normal IDC) and the genes over-expressed in the schizont stages represent mainly ring and trophozoite specific transcripts (Figure 1c). The genes up-regulated in the trophozoite stage are evenly distributed between ring and schizont specific transcripts. This indicates that the transcriptional up-regulations associated with artemisinin resistance constitute either accelerated timing of transcription of trophozoite and schizont genes at the ring stage, or prolonged expression of ring and trophozoite transcripts in the schizont stage. Intriguingly, this pattern was not observed for down-regulated genes whose expression in artemisinin resistant parasites coincides with their expected stage specificity, albeit at lower levels than expected in the artemisinin sensitive isolates (Figure 1c). Taken together, these results suggest that artemisinin resistance is associated with specific modifications of the IDC transcriptional cascade that involve a large number of genes and presumably alter the levels and temporal distributions of biological and cellular functions.

### Functional analysis of artemisinin resistance associated genes

**Figure 2:**
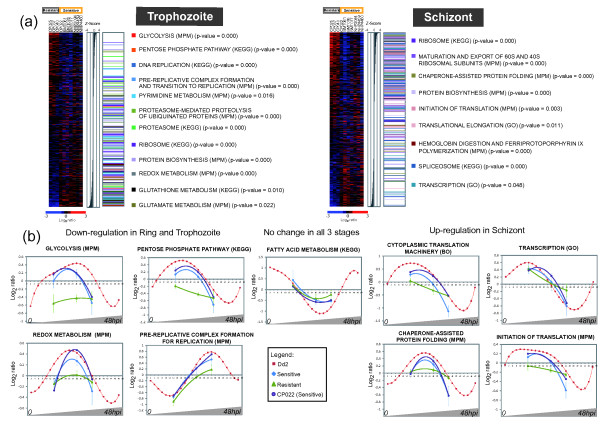
**Functional analyses of differentially expressed genes in the artemisinin resistant parasites**. (a) The heat maps depict differential gene expression in the trophozoite and schizont stages and the mean-centered log_2 _ratios of mRNA levels between the resistant and susceptible isolates (for the complete set of all stages, see Additional file 6). Here the genes were ranked according to descending z-score (SNR) by correlating the expression profiles to the phenotypic class. Gene Set Enrichment Analysis (GSEA) revealed functional pathways down-regulated in trophozoites and up-regulated in schizonts of the resistant parasites shown in the side bar diagrams and ordered by the nominal p-value. Positions of the genes belonging to each identified pathway are indicated by the colored bars in the corresponding z-score ordered gene distribution. (b) Graphs illustrate several functional pathways with significant differential expression between the resistant and susceptible parasites in the three stages. Data points represent the average log_2 _expression ratio for the isolates in each of the groups and across all genes belonging to the pathway for ring (14 hpi), trophozoite (26 hpi) and schizont (34 hpi) stages of the resistant (green triangle) and susceptible (blue diamond) isolates. Plotted are best fit polynomial curves and error bars that indicate the standard deviation amongst the isolates. Included are the average expression ratios for the artemisinin sensitive Cambodian isolate, CP022 (purple circle). For reference, the data are projected onto the centered mRNA abundance profiles of the *in vitro *IDC transcriptome (red square).

To evaluate the physiological relevance of the identified differential gene expression, we utilized Gene Set Enrichment Analysis (GSEA) [24] to explore functional assignments of genes associated with artemisinin resistance (Figure 2; Additional file 6). We found that genes down-regulated at ring and trophozoite stages represent well established biochemical and cellular pathways such as glycolysis, pentose phosphate shunt, REDOX, nucleotide and glutathione synthesis, and the TCA cycle (Figure 2a, b). Under normal growth, these pathways reach transcriptional peaks during the early stages of the IDC, so down-regulation indicates reduced expression in young parasites. In addition, we observed a significant down-regulation of genes associated with DNA replication and protein degradation in the trophozoite and schizont stages (Figure 2a, b; Additional file 6, 7). This down-regulation may represent a delayed onset of expression that normally starts during at the ring/trophozoite transition. At the schizont stage, the artemisinin resistant parasites were characterized by marked over-expression of genes that belong to many pathways associated with protein synthesis, folding and trafficking (Figure 2a, b). These include genes involved in ribosome assembly and maturation, chaperone-assisted protein folding, translational initiation and elongation (Additional file 6, 7). In addition, we observed up-regulation of several additional pathways such as RNA metabolism and hemoglobin degradation, both of which could contribute to increased capacity of protein synthesis in these parasites by boosting global levels of RNA transcripts and concentration of amino acids produced by the food vacuole (Additional file 6, 7). Investigating individual genes in the affected pathways, we observed that while in some cases the differential expression affects most genes (such as glycolysis or REDOX), in other pathways (such as translational initiation or proteasome degradation), only a fraction of genes exhibit differential expression (Additional file 8). These latter genes may represent crucial regulatory or rate limiting steps in these biological processes.

In summary, our data suggest that the specific modulation of the IDC transcriptional cascade observed in artemisinin resistant parasites has at least two major physiological implications that include: down-regulation of metabolic and cellular pathways in the first half of the IDC (up to 28 hpi) and prolonged transcription up-regulation of functionalities associated with protein synthesis and their supporting activities in the late stages (~36 hpi). Interestingly, this transcriptional program was observed in all three Western Cambodian isolates with the delayed clearance but not in the isolate collected in the same region (CP022) that had a normal clearance time (Figure 2b; Additional file 7). This suggests that the altered transcriptional pattern is not a simple reflection of geographical or other generic differences between *P. falciparum *isolates but is indeed associated with artemisinin resistance.

### Differential expression of regulatory proteins may contribute to artemisinin resistance

**Figure 3:**
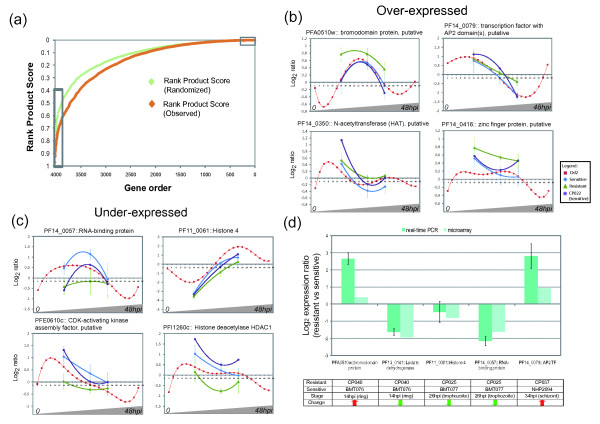
**Classification of differentially expressed genes across the whole IDC and validation of differential expression of 5 genes by qPCR**. (a) Plot of the Ranked Product Score for all 4,015 genes calculated from the geometric mean of the ranked z-scores of the genes in the 3 stages in the generated transcriptome (orange) and in the randomized dataset (green) (for details see material and methods). The graphs represent 4 over (b) and 4 under-expressed (c) genes in the resistant parasites with potential regulatory functions present in the top 5% of each extreme of the rank product distribution, respectively ((a) grey boxes). Data points represent the mean log_2 _expression ratios of the genes in the resistant (green triangle), susceptible Lao and Thai (blue diamond), and susceptible Cambodian isolates (CP022, purple circle) across the three selected stage intervals. The data are projected onto the gene expression profiles analyzed by the *in vitro *IDC transcriptome (red square). Error bars reflect the standard deviation of the log_2 _ratios for each data point. (d) Bars represent the log_2_-transformed fold change measured from relative quantification of a resistant versus a sensitive isolate using PFC0965w as a reference control gene in real-time PCR experiments (teal) and plotted alongside the microarray expression ratios (light blue). Error bars reflect the standard deviation of the log_2 _ratios over triplicates.

Artemisinin resistance in Western Cambodia was shown to exhibit a genetically inheritable pattern [20]. Thus it is feasible to speculate that the broad transcriptional changes associated with this phenotype may have resulted from a small number of genetic mutations in key regulatory proteins such as global transcription factors, chromatin remodeling-associated proteins or cell cycle regulators. We sought to explore the transcriptional data to find further clues for such underlying genetic determinants. For that, we inspected the genes whose expression was changed consistently in the three individual stages as these may be linked with a putative genetic variation (possibly in their regulatory elements). To identify such genes, we determined the rank product score for differentially expressed genes and classified them based on their differential expression in all 3 stages (Figure 3a; Additional file 5). Interestingly, the rank distribution was skewed towards lower values where larger numbers of genes show lower than expected rank scores (Figure 3a). This indicates that there are more genes consistently down-regulated in the artemisinin resistant parasites in all three stages compared to the up-regulated genes that tend to be over-expressed in only one or two of the IDC developmental stages.

Functional enrichment analyses of the top 5% of genes from both extremes of the distribution (see Figure 3a grey boxes) did not uncover any additional functional groups compared to the stage-wise GSEA (Figure 2), however, visual inspection of these gene groups identified 41 putative transcription regulators, of which 22 are under-expressed and 19 are over-expressed. These include transcription factors, RNA binding proteins, cell cycle regulators, chromatin remodeling associated proteins, histone modification enzymes as well as histones (Figure 3b, c, for complete list see Additional file 5 Sheet "Rank product score"). The most remarkable examples are strong down-regulation of genes coding for histone deacetylase 1 protein (HDAC1) (PFI1260c), recently proposed to play a major role in the transcriptional regulation [25], and CDK-activating kinase assembly factor (PFE0610c) whose paralogue was shown be associated with HDAC1 in *P. falciparum *[26]. Also, the artemisinin resistant parasites show a considerable up-regulation of the AP2 containing transcription factor (PF14_0079), bromo-domain containing protein (PFA0510w), putative histone acetyltransferase (*hat*; PF14_0350), zinc finger protein (PF14_0416), and the *P. falciparum *homologue of yeast histone chaperone Rttp106-like transcriptional regulator (PFE0870w). In addition to these DNA interacting regulatory proteins, we identified 10 genes encoding the RNA-recognition motif (RRM) that are involved in regulating mRNA stability or translational repression. Quantitative real-time PCR carried out with 4 of these genes (PFA0510w, PF14_0079, PF11_0061 and PF14_0057) further confirmed that these genes are significantly differentially expressed between the resistant and sensitive isolates (Figure 3d). Differential expression of these genes encoding regulatory factors may contribute to the global changes in the transcriptome observed in the artemisinin resistant parasites.

### Copy number variations (CNV) and genotypes of artemisinin resistant isolates

**Figure 4:**
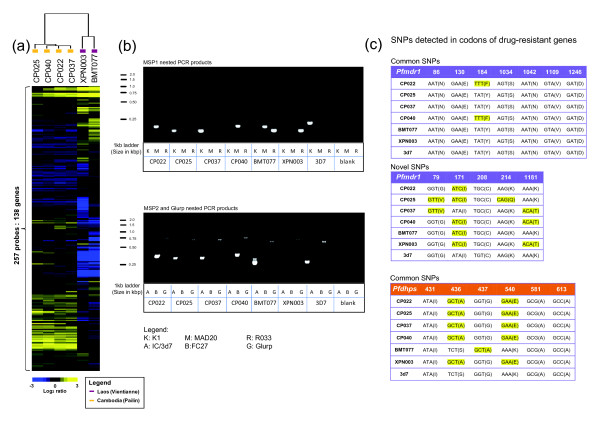
**CGH analysis of artemisinin resistant isolates, Genotyping of isolates and Sequencing of drug-resistant genes to determine haplotypes**. (a) The heat map represents hierarchical clustering of CGH signal (against 3d7 genome) for 257 microarray oligonucleotide elements representing 138 genes in 93 CNV regions identified by GADA analysis (see material and methods). (b) Visualization of *msp1*, *msp2 *and *glurp *nested PCR products of the 6 isolates on a 2.5% agarose gel shows distinctive patterns of product sizes for each clone, except for CP025 and CP037. (c) Tables depict the commonly and rarely found single nucleotide polymorphisms (SNPs) found based on the codon position (top row) and the sequenced bases of the various isolates and corresponding amino acid in brackets. Highlighted in yellow are the codons with mutations compared to wild type 3d7.

Several recent studies have demonstrated a frequent occurrence of gene copy number variants (CNV) in *P. falciparum *and linked these to differential gene expression in field isolates and laboratory strains [27-32]. This can contribute to the variability in drug sensitivities observed among isolates [33,34]. We carried out comparative genomic hybridizations with genomic DNA of the four Cambodian and two Lao isolates and demonstrated that CNV profiles exhibit a clear segregation between these two groups (Figure 4a). Among the 138 genes occurring within the 93 CNV detected segments are Histone 4, GTP cyclohydrolase I (*pfgch1*), *hyp4/5 *exported proteins, *phist *genes and Maurer's cleft two transmembrane proteins (*pfmc-2tm*) [27-32]. Increased copy number of *pfgch1 *has been previously linked with resistance to antifolate antimalarial drugs and its amplification in the Cambodian isolates (p-value = 0.002) may reflect the prolonged use of these drugs in this region [35]. Other newly identified CNVs include several hypothetical genes; an autophagocytosis associated protein and Zn^2+^/Fe^2+ ^permease (data not shown). Overall we observed strong similarities amongst the Cambodian isolates, however none of the identified CNV could be associated with artemisinin resistance. This genetic coherence among the four Cambodian isolates contrasts with their transcriptional heterogeneity and indicates that copy number differences are strongly associated with differentiation in geographical origins. More genetic studies with large number of isolates will be required to assess the role of CNVs in artemisinin resistance.

In addition to the CGH analysis, we carried out "standard" genotyping of genes coding for merozoite surface proteins: *msp1*, *msp2 *and glutamate-rich protein: *glurp *together with sequencing of drug-resistant markers: multidrug resistant protein 1 (*pfmdr1*) and dihydropteroate synthase (*pfdhps*). This analysis revealed that the four Cambodian and two Lao isolates are genetically distinct from each other carrying different alleles of the tested genes (Figure 4b). Of these, CP025 and CP037 appeared highly related, exhibiting identical *msp1 *and *msp2 *nested PCR products of KO33 and IC/3d7 alleles (Figure 4b). However, these two isolates differ in 2 non-synonymous SNPs of codons K214Q and K1181T and a synonymous SNP in codon 171 in the *pfmdr1 *gene (Figure 4c). In addition, CP025 and CP037 exhibit subtle differences in their CNV pattern as measured by CGH (Figure 4a). Interestingly, we found that none of the 6 isolates carry the N86Y *pfmdr1 *mutation (Figure 4c) whose presence is frequent in many Southeast Asian regions [36] and which is commonly associated with chloroquine resistance and mefloquine hypersensitivity [37]. Furthermore, we observed significant haplotype sharing amongst analyzed isolates such as CP040 and CP022 that share the "NEFSNVD" haplotype of *pfmdr1*, containing the non-synonymous Y184F mutation. Moreover, CP025, CP037, CP040, CP022 and XPN003 share the IAGEAA haplotype of *pfdhps *with non-synonymous triple mutants S436A, G437A and K540E, which were previously linked to resistance to sulfadoxine-pyrimethamine [38,39]. The mutations in *dhps *may reflect the selection pressure as a result of an extensive antifolate drug use in this region over several decades. In summary, these genetic studies suggest that while the Western Cambodian isolates are not identical isogenic clones, they share a recent common ancestor that is evident from their CNV profiles. Hence, the highly unique and also uniform CNV pattern identified in these parasites may represent a genetic background that contributes to development of artemisinin resistance and possibly other drug resistant phenotypes in this region.

## Discussion

### Mechanism of artemisinin resistance

Artemisinin resistance of *P. falciparum *is a major threat to malaria control. Understanding its molecular basis is thus essential for determining treatment strategies, mapping the spread of resistance and guiding elimination [20,40-44]. Mutations or amplification of genes encoding transporters or target enzymes have been identified as resistance mechanisms to other antimalarial drugs. It is possible that resistance to artemisinin is unlike these classical mechanisms *in vitro *but instead results from a complex series of genetic and epigenetic events affecting multiple pathways. Here we showed that artemisinin resistant parasites are characterized by a specific modulation of the IDC transcriptome that affects a broad spectrum of genes and biological functions. First, we detected a specific down-regulation of many ring stage-specific metabolic pathways such as energy metabolism, nucleotide synthesis etc. Second, the progress to mature schizont stages was marked by significant increase in transcription of genes associated with essentially all functionalities involved in protein metabolism (Figure 2). Based on these two observations, we propose a plausible model for molecular basis of the artemisinin resistance observed in these Cambodian isolates that is founded on two (possibly independent) events: (i) Limited metabolic activity of the ring stages could lead to lower levels of hemoglobin digestion, less ferrous ions produced and thus reduced activation and conversion of artemisinin drugs to reactive intermediates [45]. This effect may be linked to the high prevalence of hemoglobinopathies, especially Hemoglobin E and α-thalassemia in this area conferring increased oxidant stress [46,47]. This is possibly compounded by decades of unregulated artemisinin use in Western Cambodia which may select for parasites with greater resistance to oxidant stress during the ring form development. Alternatively, the lower metabolic activities of the ring stages may be a prerequisite of the ability of the resistant parasite to become dormant under the artemisinin pressure as demonstrated by several *in vitro *analyses [17,18]. Although this phenomenon is unlikely to be the major factor in artemisinin resistance, it is worth noting that the results obtained in our study are in agreement with the transcriptional analyses of the *in vitro *derived artemisinin tolerant parasites that include mRNA levels of the hypoxanthine phosphoribosyl transferase (PF10_0121), a cell cycle regulator (PFE1415w) and a Heat Shock Protein 70 kDa (PF08_0054) (Additional file 9) [40]. (ii) The increased activity of protein metabolism may contribute to the resistance by withstanding the damaging effects of artemisinin on parasite's proteins by increasing rates of protein turnover and synthesis and thus compensate for loss of active proteins. This is consistent with the presumed artemisinin mode of action in which the drug inflicts substantial damage to the parasite cell either by an oxidative stress (via reactive free radicals) or by direct alkylation of a wide spectrum of cellular components such as proteins, heme, and lipids [48-53].

### Transcriptional regulation and artemisinin resistance

It is important to note that the differences in mRNA levels linked to artemisinin resistance were not the most dramatic transcriptional differences detected among the field isolates. Functional enrichment analysis of genes that vary the most in their expression among the 11 field isolates irrespective of their artemisinin sensitivities, revealed a distinct set of functionalities that varied in gene expression amongst field isolates in general. These include mainly genes encoding proteins of host-parasite interactions, Maurer's clefts proteins, invasion-related proteins, as well as factors of fatty acid metabolism, glycolysis and hemoglobin digestion (Additional file 10). This indicates that the observed transcriptional differences between artemisinin resistant and sensitive strains may represent a new event in transcriptional differentiation of field isolates that does not occur in other parasite populations. Although very little is known about molecular factors of transcriptional regulation in *P. falciparum*, a number of previous studies [25,54-57] have indicated the existence of broad range regulatory mechanisms that affect large groups of genes controlling the progression of the *Plasmodium *life cycle. Although more studies that involve large epidemiological surveys followed by extensive molecular analyses will have to be carried out to support such putative mechanisms, here we explored genes whose expression was affected in all three developmental stages in the resistant Western Cambodian parasites. One of the most remarkable examples of consistently down-regulated genes is *pfhdac1 *and its putative interacting proteins partner CDK-activating kinase, both of which show dramatic down-regulation in all three developmental stages (Figure 3). In our recent study, we showed that HDAC activities play a highly dynamic role in regulating the transcriptome of the *Plasmodium *life cycle and that their inhibition leads to widespread transcriptional changes. Intriguingly, the transcription profile of the resistant parasites is reminiscent of the effect of HDAC inhibitors on the *P. falciparum *gene expression in which most gene induction represent accelerated transcription in the ring and trophozoite stages and prolonged mRNA expression during the schizont stage [25].

Another gene whose differential expression is significant is histone 4 (PF11_0061). Histone 4 is the only nucleosomal subunit present as a single copy gene and thus its down-regulation could have severe implication on the nucleosomal assembly during the *P. falciparum *schizont stage. Depletion of histone 4 in both yeast and human cells causes an arrest or dramatic delay in the progression of the S-phase, which is caused by insufficient nucleosomal assembly and subsequently inhibition of DNA synthesis [58,59]. We observed a modest but statistically significant decrease of histone 4 in the artemisinin resistant *P. falciparum *parasites (Figure 3d) that could analogously lead to a delayed onset of schizogony and thus prolonged expression of genes associated with the typical trophozoite functions such as protein synthesis and hemoglobin digestion (Figure 2). Since the CNV profiles were similar among the four Cambodian isolates regardless of their tolerance level to artemisinin, more studies will be needed to understand their role in regulating transcriptional levels in artemisinin resistant parasites. More importantly, the coherence of CNV profiles among the Cambodian isolates indicates that the parasites in this region share a recent common ancestor. Similar conclusion was made by an admixture study carried out in Southeast Asia that demonstrated a sub-group of recently expanded parasite populations in Western Cambodia [60]. This could be a result of intense administration of a successive number of antimalarials from chloroquine to mefloquine to sulfodoxine-pyrimethamine to treat malaria cases in the Western Cambodian region over the last 80 years. It is plausible that a population with a restricted genotype has emerged with a genetic background that has higher propensity to give rise to resistance of the *P. falciparum *parasites to artemisinin.

## Conclusions

Here we present the most comprehensive transcriptional analysis of the artemisinin tolerant malaria parasites collected at the site of its origin, Western Cambodia. Although much more extensive research on multiple levels will be needed to elucidate the complete mechanism, the results from these studies provide the first testable hypotheses regarding both the global pattern of the transcriptional cascade and candidate genes involved in artemisinin resistance.

## Methods

### Sample collection from patient and during ex-vivo IDC

On admission to the hospital and upon consent, 10 ml of total infected blood was taken from each adult *P. falciparum*-infected patient from the various field sites (Pailin, Cambodia; Xepon, Savannakhet Province, Laos; Mae Sot, Thailand). Samples were filtered by CF11 column purification to remove white blood cells and obtain infected red blood cells which were subjected to *in vitro *culture for one generation of the IDC over 48 hours. For every isolate, a total of 6 to 10 samples were harvested at regular intervals of 2 to 8 hours throughout the IDC. 100-500 μl of packed red blood cells with 1% - 10% parasitemia were collected per time point sampling.

### RNA extraction, cDNA synthesis & Microarray Hybridizations

Total RNA was isolated from all *ex-vivo *samples using the trizol-chloroform-isopropanol precipitation method as described [21]. Synthesis of target DNA for microarray hybridization was carried out as previously described [27,61] with the following exceptions: Reverse-transcription was carried out using 200 units of SuperScript II enzyme (Invitrogen, USA) to create the first strand cDNA. Following PCR amplification with 19 cycles and purification of the product, a total of 5-7_g of double-stranded DNA was obtained for each sample. From this, 4 μg of DNA was labeled with fluorescent Cy5 dye and used for the microarray hybridization (GE Amersham, USA). For the reference pool, cDNA consisting of all IDC stages of 3d7 strain was made identically and labeled with Cy3 dye. Equal amounts of labeled sample from each time point and reference RNA pool was hybridized on the 70-mer *P. falciparum *cDNA microarray chip containing 10,680 oligonucleotides representing 5,343 coding genes [62]. The microarray hybridization was carried out at 65°C in the automated MAUI hybridization system (BioMicro Systems, USA). The microarray chips were scanned using the GenePix 4000 B scanner and GenePix Pro 6.0 software (Molecular Devices, USA).

### Microarray data Processing and analysis

The initial acquired microarray data was filtered to include microarray hybridization "spots" with at least 95% pixels having signal intensity above 2 standard deviations from background for both Cy3 and Cy5 fluorescence intensity. Subsequently, array Lowess normalization was applied to all arrays as implemented by Acuity 4.0 software (Molecular Devices, USA). The assembly of the IDC transcriptome for all isolates was carried out using Fast Fourier Transform as previously described [21] with following modifications; the expression value for each gene is represented by an average value obtained from all oligonucleotide probes in each transcript/gene. To construct the phaseogram of reference IDC transcriptome for further comparisons, genes with expression data in at least 19 out of 24 (80%) time points were extracted, any missing values were imputed and polynomial fit of order 6 was applied to generate smoothed data. This was then analyzed by Fast Fourier Transform method and all 4,634 genes with the mean-centered gene expression log_2 _ratios were sorted according to phase from -π to π (Additional file 2). The phaseogram of each isolate contains mean-centered gene expression data ordered according to the phase value. For each isolate's time course, only genes having at least 80% of data present were extracted and any missing data values were imputed by KNN (K^th ^nearest neighbor) method using R version 2.10.1 package and *impute *function.

### Mapping parasite's sample age to the reference IDC

To map the age of each isolate time point sample relative to the progression of *in vitro *lab strain IDC, Spearman Rank Correlation Coefficient (SRCC) values between global mRNA profiles for each isolate time point and time points in the reference IDC transcriptome [22] (every 2 hour sample time point of the *in vitro *Dd2 lifecycle) were calculated. The stage (hpi) corresponding to the peak SRCC value was assigned as the best estimate of the age of the parasite for that sample collection (see colored box in Figure 1b). Only samples which had SRCC above a value of 0.35 were considered in the following analysis. To select time points for downstream differential analysis, we calculated the frequency distribution of isolate time points found within a window of three consecutive hpi and selected three windows based on the highest representation of isolates with at least one sampling time point found within that window. Isolates with sample time points found within the 4 hour windows of 12-16 hpi, 24-28 hpi and 32-36 hpi corresponding to mid-ring, early-trophozoite and early-schizont stages were grouped accordingly as representing these stages.

### Identifying Differentially Expressed Genes

Z scores were calculated by correlating the average gene expression to noise ratio (SNR) of the mRNA expression ratios to the clearance phenotype (ie. resistant - CP025, CP037, CP040 vs. sensitive - CP022, BMT061, BMT077, BMT076, XPN003, NHP2094, NHP4459, NHP4460) and normalized to sample size.

Where *μ_a _*and *μ_b _*are the mean expression log_2 _ratio of resistant and sensitive parasites respectively and *σ_a _*and *σ_b _*are the standard deviations of resistant and sensitive group respectively. Differentially expressed genes were defined with p-value cut-off < 0.01 from Student t- distribution of the z-scores carried out for the 3 stages. Gene set enrichment analysis [24] was carried out on the resistant vs. sensitive parasites using SNR. A total of 1,261 gene sets comprising of KEGG pathways [63], MPM and functional groups based on previous studies [21,64-66] were included. Nominal p-value and false discovery rate (FDR) were calculated for each gene set from the observed normalized enrichment score (NES) against a null distribution using a gene set-based permutation test. Gene sets with p-value < 0.05 and FDR < 25% were considered to be statistically significant.

### Calculate Timing of Peak mRNA abundance of genes

The time of peak expression for each gene (represented by hpi) is derived from the phase of the Fourier transformation of the *in vitro *reference transcriptome using the formula:

Where *θ *is the phase value of the gene.

### Calculation of Rank Product Score

Using the z-scores (see above) of each gene, we sorted the genes according to descending order and assigned a numerical rank score for every gene at each of the three stages. In order to generate a list of genes consistently up/down-regulated in the three stages, we calculate the geometric mean of the rank score to determine the rank product (RP) score of each gene using the formula:

Where the rank_g_^14hpi^, rank_g_^26hpi^, rank_g_^34hpi ^corresponds to the rank score of the gene at 14 hpi, 26 hpi or 34 hpi respectively.

### Copy Number Difference Analysis

To identify significant CNVs among the 6 isolates, we performed comparative genomic hybridization using genomic DNA extracted from the isolates (CP022, CP025, CP037, CP040, BMT077, XPN003) against genomic DNA of the reference lab strain of 3d7 as previously described [27]. We carried out subtraction analysis between any 2 isolates in multiple pair-wise comparisons and considered only segments that have at least 1.7-fold change in mean amplitude and T-coefficient greater than 3.5 in minimum 2 consecutive gene probes between any 2 isolates as significant. This was performed using the Genomic Alteration Detection Analysis (GADA) program in R package [67].

### Real-time PCR (qPCR)

Real time PCR and the relative quantification of gene expression was performed in at least triplicates using the comparative CT method of calculating -ΔΔCt values and taking PFC0965w as a reference control gene, as previously described [62].

### Genotyping of isolates and Sequencing of *pfmdr1 *and *pfdhps *genes

Genotyping of *msp1*, *msp2 *and *glurp *were carried out as previously described [68] with positive control of lab clone, 3d7, in a nested PCR reaction and products were visualized on a 2.5% agarose gel. Nested PCR reactions were performed as previously described [69] to generate fragments of *mdr1 *and *dhps *genes containing commonly found SNPs and products were sequenced with corresponding forward and reverse primers using ABI Big Dye Terminator cycle sequencing method.

## List of abbreviations used

IDC: Intra-erythrocytic developmental cycle; hpi: hours post invasion; PCT: Parasite clearance time; SRCC: Spearman rank correlation coefficient; PCC: Pearson correlation coefficient; SNR: Signal to noise ratio; NES: Normalized enrichment score; GSEA: Gene set enrichment analysis; KEGG: Kyoto Encyclopedia of Genes and Genomes; MPM: Malaria metabolic pathway maps; GO: Gene Ontology; CNV: Copy number variants; PCR: Polymerase chain reaction; SNP: Single nucleotide polymorphism

## Authors' contributions

MI, KC, NPJD, NJW, PRP, AMD and ZB designed research. SM, JS and KYL performed research. MI organized field work. MI, MM, PNN, FN, BR, PY, DS, KC and AMD organized/provided sample collection. SM, MJM, RR and ZB analyzed data. SM and ZB wrote the paper. SM, MI, MJM, PRP, MM, PNN, FN, NPJD, NJW, AMD and ZB revised the manuscript. The authors declare that they have no conflict of interest. All authors read and approved the final manuscript.

## Supplementary Material

Additional file 1 **Parasite density over course of treatment and treatment outcomes**. Plot of the parasite densities (relative to starting parasite numbers on admission) from the time of patient's 1^st ^admission date up to 100 hours after admission. Of the 4 isolates from Pailin, Cambodia, only 3 (CP025 (red), CP037 (green), CP040 (blue)) display significant delayed parasite clearance time (pct) of 78 and 96 hours from the patients after treatment while CP022 (purple) parasites was cleared earlier at 54 hours after treatment. The initial parasite numbers upon admission (para0) and parasite reduction ratio at 24 hours (prr24) and 48 hours (prr48) are included in the table. Artesunate and mefloquine treatment regimes administered to the patients are listed (box). Click here for file

Additional file 2 **Information about the sampling times, parasitemia of the ring, trophozoite and schizont stages of the 11 isolates**. na: information not available. Click here for file

Additional file 3 ***Ex-vivo *transcriptomes generated of the 11 *P. falciparum *field isolates from South East Asia**. Transcriptomes of the ex-vivo IDC of all 11 field isolates from the 3 geographical locations measured over 48 hour sampling time. Only genes with at least 80% of time points with a positive signal were included for each of these transcriptomes. The phaseograms were constructed by ordering the mean-centered log_2 _microarray expression ratios to the genes ordered by phase calculated from the Fast Fourier Transformation of the Dd2 reference *in vitro *lifecycle. Click here for file

Additional file 4 **Distribution of Pearson correlations between all isolates for the 3 stages**. Including isolate time points that correspond to the 3 stages - 14 hpi, 26 hpi and 34 hpi - graphs shown are average Pearson Correlations calculated from multiple pair wise comparisons between all isolates (graphs on left panel) and the average of the PCC for the resistant and sensitive parasites (graphs on right panel) at 14 hpi (a), at 26 hpi (b) and at 34 hpi (c). Error bars represent the standard deviation of all the pair-wise comparisons. Click here for file

Additional file 5 **Sheets 1-3 lists log_2 _expression data of all genes for the isolates grouped by ring (14 hpi), trophozoite (26 hpi) and schizont (34 hpi) stage and their corresponding z-scores (signal to noise) and p-values using student t-distribution**. Genes with significant difference in expression between the resistant and sensitive isolates (p-value < 0.01) are highlighted in orange. Sheet "rank product score" lists all genes including putative transcriptional regulators (including those with zinc finger, bromodomain, etc., domains predicted by PFAM, Histone/HDAC/HAT-related, AP2-domain containing and potential cell cycle regulators) used in the analysis and their corresponding z-scores and rank product scores for ring (14 hpi), trophozoite (26 hpi) and schizont (34 hpi) stages. Click here for file

Additional file 6 **Functional analyses of differential expression in all stages of the artemisinin resistant parasites**. For each of the 3 stages, genes were ranked according to the z-score by correlating the expression profiles to the phenotypic class. The mean-centered log_2 _ratios for each gene of the resistant (CP025, CP037, and CP040) and sensitive (CP022, BMT061, BMT076, BMT077, XPN003, NHP2094, NHP4459, NHP4460) isolates are represented in these clusters. Gene Set Enrichment Analysis [24] of the ranked clusters gave rise to gene sets down-regulated in rings and trophozoites and up-regulated in schizonts in the resistant parasites as shown ordered by the nominal p-value, false discovery rate (FDR) q-value and Normalized Enrichment Score (NES). Significant gene sets were based on cut-off p-value of 0.05 and FDR q-value of 0.25. Click here for file

Additional file 7 **Functional pathways with significant differential expression in rings, trophozoites and schizonts of artemisinin resistant and sensitive parasites**. Gene sets (functional groups) which are denoted in the graphs are obtained from various data sets of previous studies [63,66,70] and plotted are all pathways that are significantly differentially expressed in artemisinin resistant parasites in at least one IDC stage. For each pathway, each data point at ring (14 hpi), trophozoite (26 hpi) and schizont (34 hpi) of the resistant (green triangle) and sensitive (blue diamond) series was calculated from taking the average of the expression log_2 _ratio of a gene across all isolates in that phenotypic group (resistant or sensitive) and then averaged for all the genes associated with that pathway. Best fit polynomial curves were plotted (lines). Error bars indicate the standard deviation among the isolates for that pathway. Included separately are the average expression ratios of all genes belonging to that pathway for CP022 isolate (purple circle) from Pailin, Cambodia and reference *in vitro *strain (red square). Click here for file

Additional file 8 **Relative expression of individual genes associated with four functional pathways that have significant differential expression in artemisinin resistant parasite**. Top: Example of four functional gene sets with significant differential expression between resistant and sensitive parasites and the relative gene expression of all members of the gene sets at the three stages. Each set of data points are the average log_2 _expression ratios of the isolates in a group and averaged for all the genes in that pathway at 14, 26 and 34 hpi. The curves are the best fit polynomial curves to the data points. Bottom: Each of the 3 graphs plotted depict the average log_2 _gene expression ratios of the isolates in the resistant (green triangle) or sensitive (blue diamond) group with the standard deviation represented by error bars in each particular pathway at14, 26 or 34 hpi. Click here for file

Additional file 9 **Transcriptional profiles of the 3 genes in the artemisinin resistant parasites compared with previous data published **[18]. The mean expression log_2 _ratio (data point) and SD (error bars) among the resistant (green triangle) and sensitive (blue diamond) isolates for each stage are plotted for the genes: PF10_0121 - hypoxanthine phosphoribosyltransferase (rank 3207/4029; p-value = 0.1), PF08_0054 - Heat Shock Protein 70 kDa (rank 14/4041; p-value = 0.0004) and PFE1415w- cell cycle regulator (rank 3837/4041; p-value = 0.01). The polynomial represents the best fit curve through the data points. The arrow indicates the approximate stage in which significant increased or lowered expression was observed in artesunate-tolerant parasites and the fold change [18] (Witkowski et al., 2010). Click here for file

Additional file 10 **Functional analysis and clustering based on general differences in gene expression among field isolates without phenotypic classification**. (a) Clusters are represented by the log_2 _expression ratios for all genes ordered according to the standard deviation (SD) for each gene in a descending manner. GSEA [24] performed on this pre-ranked list of genes identified these functional gene sets as differentially expressed among field isolates without any phenotypic classification for the 3 stages. (b) Hierarchically clustered isolates for the genes showing greatest variation in expression ratios (taking genes with SD value at the 95^th ^percentile cut off). Each color denotes the location: Laos, Mae Sot or Pailin that the isolates originate from. The raw data reported in this paper has been deposited in the NCBI's Gene Expression Omnibus database [71] and are accessible through GEO Series accession number GSE25883. http://www.ncbi.nlm.nih.gov/geo/query/acc.cgi?acc=GSE25883 Click here for file
