# Supplementary material for: Artemisinin resistance in Plasmodium falciparum is associated with an altered temporal pattern of transcription
Source: BMC Genomics. 2011 Aug 3;12:391. doi: 10.1186/1471-2164-12-391 (PMC3163569; doi:10.1186/1471-2164-12-391)
Supplement: Additional file 1 — Parasite density over course of treatment and treatment outcomes. Plot of the parasite densities (relative to starting parasite numbers on admission) from the time of patient's 1st admission date up to 100 hours after admission. Of the 4 isolates from Pailin, Cambodia, only 3 (CP025 (red), CP037 (green), CP040 (blue)) display significant delayed parasite clearance time (pct) of 78 and 96 hours from the patients after treatment while CP022 (purple) parasites was cleared earlier at 54 hours after treatment. The initial parasite numbers upon admission (para0) and parasite reduction ratio at 24 hours (prr24) and 48 hours (prr48) are included in the table. Artesunate and mefloquine treatment regimes administered to the patients are listed (box). [file 1471-2164-12-391-S1.PDF]

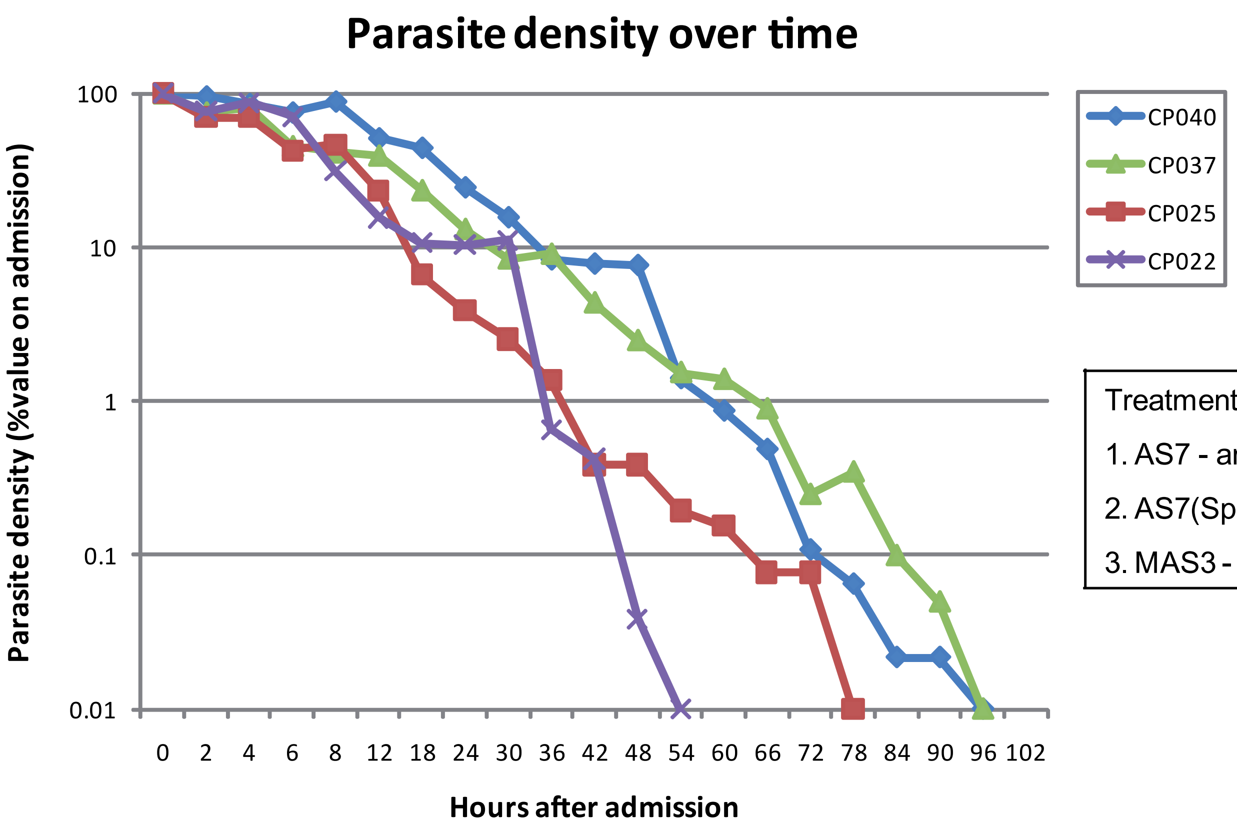

Treatment arms:

1. AS7 - artesunate 6mg/kg once daily for 7 days;

2. AS7(Split) - artesunate 3mg/kg twice daily for 7 days;

3. MAS3 - artesunate 8mg/kg once daily for 3 days followed by mefloquine;

Sample	Arm	treatcode	para0	prr24	prr48	modelpc50	modelpc90	pct
CP 022	AS7(Split)	2	103996.7969	9.627906799	2599.919922	7.168783188	18.45552826	54.00
CP 025	AS7(Split)	2	20598.40039	25.7480011	257.480011	5.413582802	17.98236847	**78.00**
CP 037	AS7	1	80384	7.619047165	40.19200134	8.547142029	29.2358799	**96.00**
CP 040	MAS3	3	73852.79688	4.083333015	13.06666565	13.860672	30.82144165	**96.00**
