# Supplementary material for: Artemisinin resistance in Plasmodium falciparum is associated with an altered temporal pattern of transcription
Source: BMC Genomics. 2011 Aug 3;12:391. doi: 10.1186/1471-2164-12-391 (PMC3163569; doi:10.1186/1471-2164-12-391)
Supplement: Additional file 3 — Ex-vivo transcriptomes generated of the 11 P. falciparum field isolates from South East Asia. Transcriptomes of the ex-vivo IDC of all 11 field isolates from the 3 geographical locations measured over 48 hour sampling time. Only genes with at least 80% of time points with a positive signal were included for each of these transcriptomes. The phaseograms were constructed by ordering the mean-centered log2 microarray expression ratios to the genes ordered by phase calculated from the Fast Fourier Transformation of the Dd2 reference in vitro lifecycle. [file 1471-2164-12-391-S3.PDF]

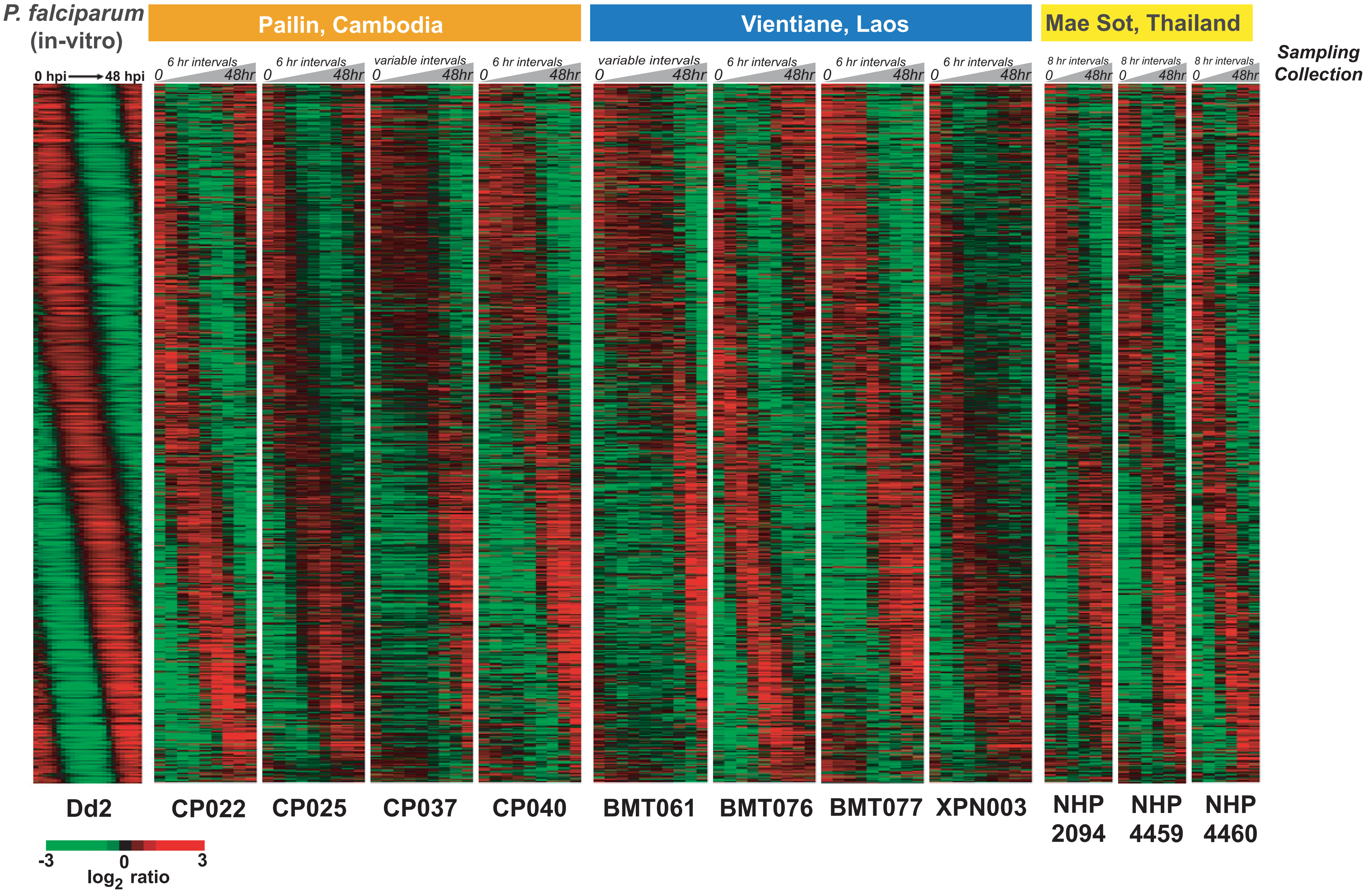

P. falciparum
(in-vitro)
Pailin, Cambodia
Vientiane, Laos
Mae Sot, Thailand
Sampling Collection
0 hpi
48 hpi
6 hr intervals
variable intervals
8 hr intervals
0
48hr
Dd2
CP022
CP025
CP037
CP040
BMT061
BMT076
BMT077
XPN003
NHP 2094
NHP 4459
NHP 4460
-3
0
3
log2 ratio
