# Supplementary figures and images for: Artemisinin resistance in Plasmodium falciparum is associated with an altered temporal pattern of transcription
Source: BMC Genomics. 2011 Aug 3;12:391. doi: 10.1186/1471-2164-12-391 (PMC3163569; doi:10.1186/1471-2164-12-391)

(a)

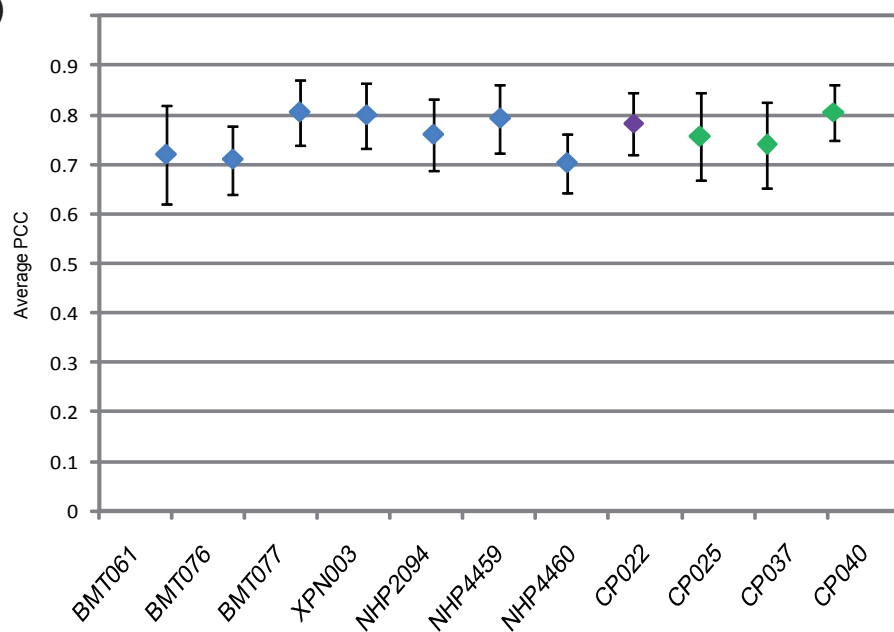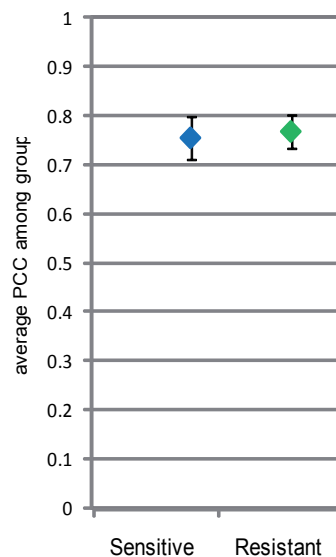

(b)

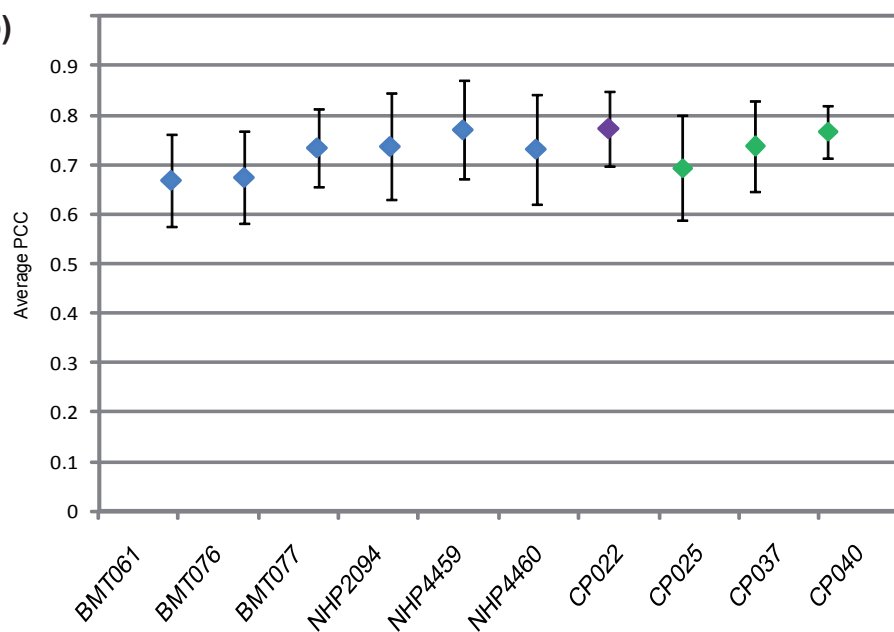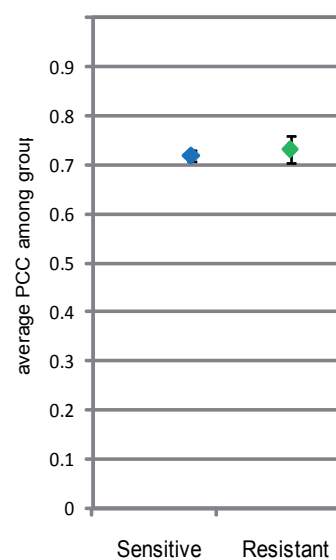

(c).

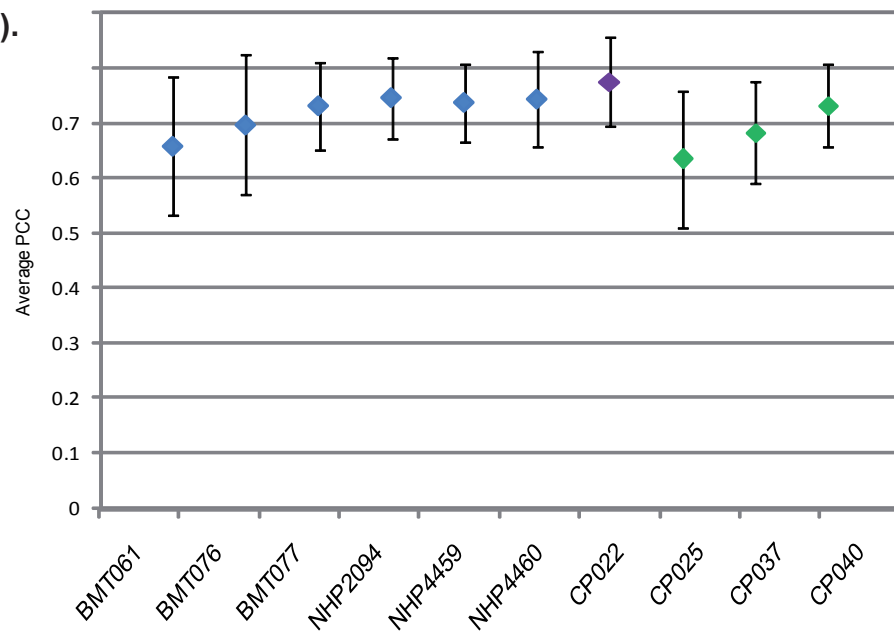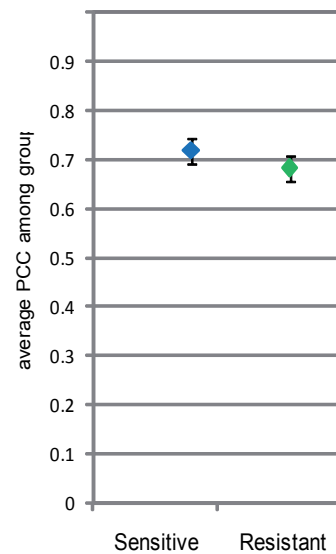

Supplement: Additional file 4 — Distribution of Pearson correlations between all isolates for the 3 stages. Including isolate time points that correspond to the 3 stages - 14 hpi, 26 hpi and 34 hpi - graphs shown are average Pearson Correlations calculated from multiple pair wise comparisons between all isolates (graphs on left panel) and the average of the PCC for the resistant and sensitive parasites (graphs on right panel) at 14 hpi (a), at 26 hpi (b) and at 34 hpi (c). Error bars represent the standard deviation of all the pair-wise comparisons. [file 1471-2164-12-391-S4.PDF]

A

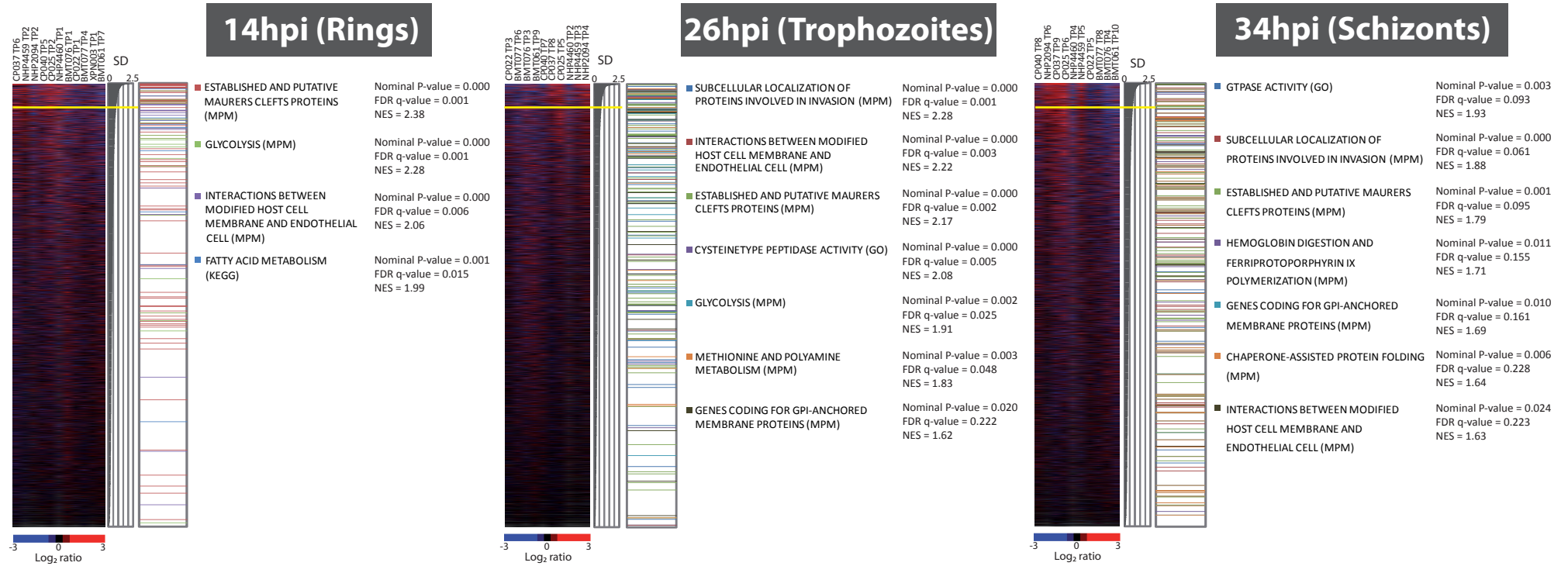

B

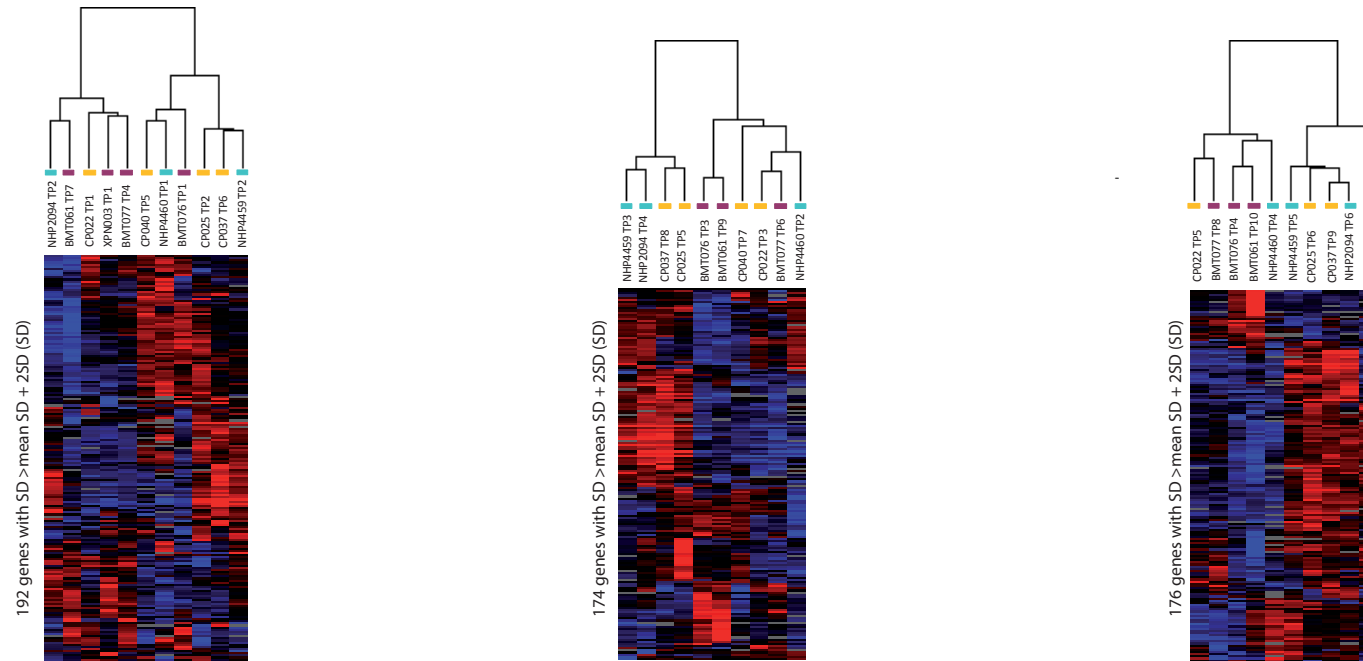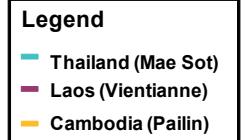

Supplement: Additional file 10 — Functional analysis and clustering based on general differences in gene expression among field isolates without phenotypic classification. (a) Clusters are represented by the log2 expression ratios for all genes ordered according to the standard deviation (SD) for each gene in a descending manner. GSEA [24] performed on this pre-ranked list of genes identified these functional gene sets as differentially expressed among field isolates without any phenotypic classification for the 3 stages. (b) Hierarchically clustered isolates for the genes showing greatest variation in expression ratios (taking genes with SD value at the 95th percentile cut off). Each color denotes the location: Laos, Mae Sot or Pailin that the isolates originate from. The raw data reported in this paper has been deposited in the NCBI's Gene Expression Omnibus database [71] and are accessible through GEO Series accession number GSE25883. http://www.ncbi.nlm.nih.gov/geo/query/acc.cgi?acc=GSE25883 [file 1471-2164-12-391-S10.PDF]
