# Supplementary material for: Artemisinin resistance in Plasmodium falciparum is associated with an altered temporal pattern of transcription
Source: BMC Genomics. 2011 Aug 3;12:391. doi: 10.1186/1471-2164-12-391 (PMC3163569; doi:10.1186/1471-2164-12-391)
Supplement: Additional file 6 — Functional analyses of differential expression in all stages of the artemisinin resistant parasites. For each of the 3 stages, genes were ranked according to the z-score by correlating the expression profiles to the phenotypic class. The mean-centered log2 ratios for each gene of the resistant (CP025, CP037, and CP040) and sensitive (CP022, BMT061, BMT076, BMT077, XPN003, NHP2094, NHP4459, NHP4460) isolates are represented in these clusters. Gene Set Enrichment Analysis [24] of the ranked clusters gave rise to gene sets down-regulated in rings and trophozoites and up-regulated in schizonts in the resistant parasites as shown ordered by the nominal p-value, false discovery rate (FDR) q-value and Normalized Enrichment Score (NES). Significant gene sets were based on cut-off p-value of 0.05 and FDR q-value of 0.25. [file 1471-2164-12-391-S6.PDF]

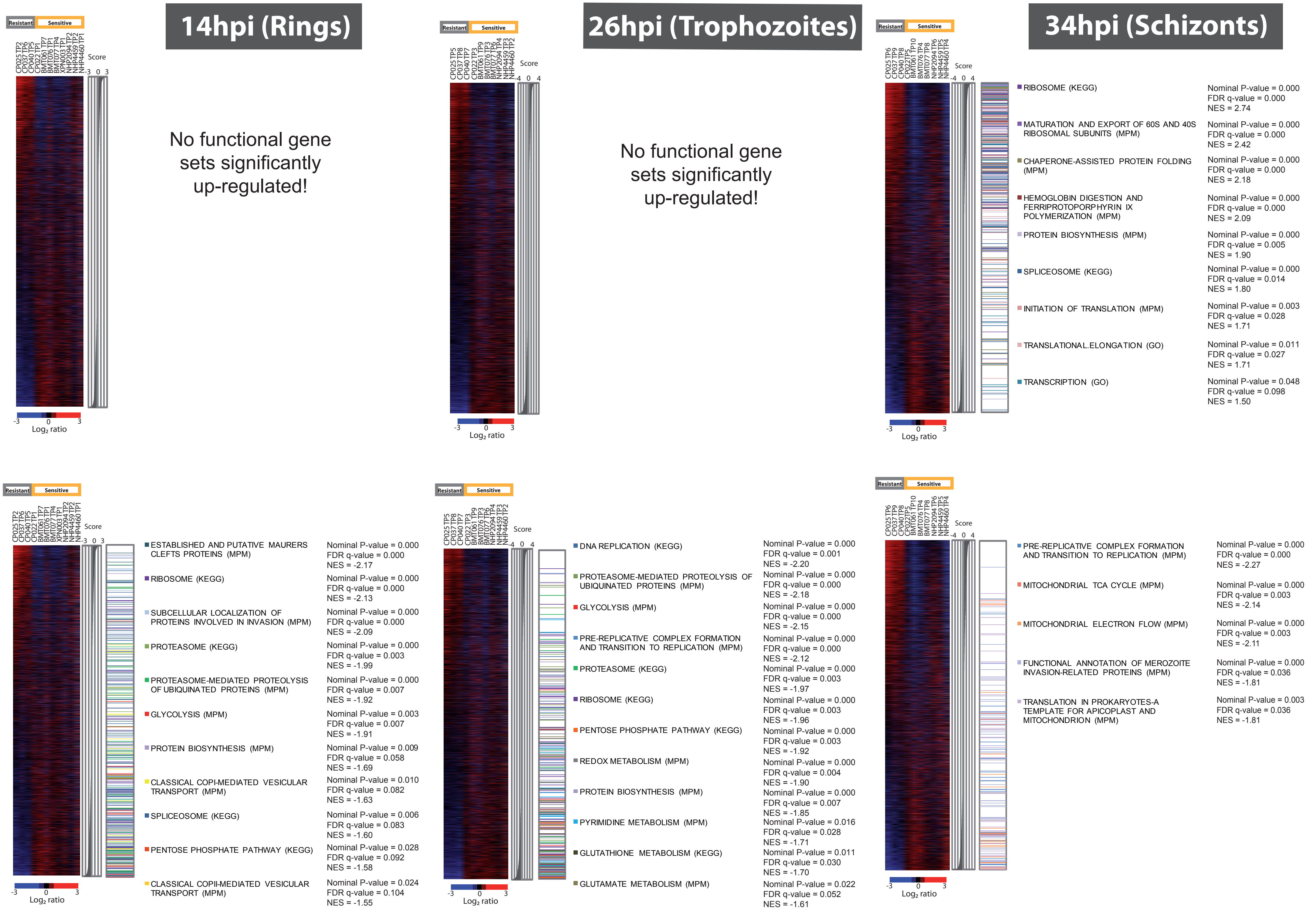
14hpi (Rings)
26hpi (Trophozoites)
34hpi (Schizonts)
Resistant
Sensitive
Score
Log2 ratio
No functional gene sets significantly up-regulated!
No functional gene sets significantly up-regulated!
RIBOSOME (KEGG)
Nominal P-value = 0.000
FDR q-value = 0.000
NES = 2.74
MATURATION AND EXPORT OF 60S AND 40S RIBOSOMAL SUBUNITS (MPM)
Nominal P-value = 0.000
FDR q-value = 0.000
NES = 2.42
CHAPERONE-ASSISTED PROTEIN FOLDING (MPM)
Nominal P-value = 0.000
FDR q-value = 0.000
NES = 2.18
HEMOGLOBIN DIGESTION AND FERRIPROTOPORPHYRIN IX POLYMERIZATION (MPM)
Nominal P-value = 0.000
FDR q-value = 0.000
NES = 2.09
PROTEIN BIOSYNTHESIS (MPM)
Nominal P-value = 0.000
FDR q-value = 0.005
NES = 1.90
SPLICEOSOME (KEGG)
Nominal P-value = 0.000
FDR q-value = 0.014
NES = 1.80
INITIATION OF TRANSLATION (MPM)
Nominal P-value = 0.003
FDR q-value = 0.028
NES = 1.71
TRANSLATIONAL.ELONGATION (GO)
Nominal P-value = 0.011
FDR q-value = 0.027
NES = 1.71
TRANSCRIPTION (GO)
Nominal P-value = 0.048
FDR q-value = 0.098
NES = 1.50
ESTABLISHED AND PUTATIVE MAURERS CLEFTS PROTEINS (MPM)
Nominal P-value = 0.000
FDR q-value = 0.000
NES = -2.17
RIBOSOME (KEGG)
Nominal P-value = 0.000
FDR q-value = 0.000
NES = -2.13
SUBCELLULAR LOCALIZATION OF PROTEINS INVOLVED IN INVASION (MPM)
Nominal P-value = 0.000
FDR q-value = 0.000
NES = -2.09
PROTEASOME (KEGG)
Nominal P-value = 0.000
FDR q-value = 0.003
NES = -1.99
PROTEASOME-MEDIATED PROTEOLYSIS OF UBIQUINATED PROTEINS (MPM)
Nominal P-value = 0.000
FDR q-value = 0.007
NES = -1.92
GLYCOLYSIS (MPM)
Nominal P-value = 0.003
FDR q-value = 0.007
NES = -1.91
PROTEIN BIOSYNTHESIS (MPM)
Nominal P-value = 0.009
FDR q-value = 0.058
NES = -1.69
CLASSICAL COPI-MEDIATED VESICULAR TRANSPORT (MPM)
Nominal P-value = 0.010
FDR q-value = 0.082
NES = -1.63
SPLICEOSOME (KEGG)
Nominal P-value = 0.006
FDR q-value = 0.083
NES = -1.60
PENTOSE PHOSPHATE PATHWAY (KEGG)
Nominal P-value = 0.028
FDR q-value = 0.092
NES = -1.58
CLASSICAL COPII-MEDIATED VESICULAR TRANSPORT (MPM)
Nominal P-value = 0.024
FDR q-value = 0.104
NES = -1.55
DNA REPLICATION (KEGG)
Nominal P-value = 0.000
FDR q-value = 0.001
NES = -2.20
PROTEASOME-MEDIATED PROTEOLYSIS OF UBIQUINATED PROTEINS (MPM)
Nominal P-value = 0.000
FDR q-value = 0.000
NES = -2.18
GLYCOLYSIS (MPM)
Nominal P-value = 0.000
FDR q-value = 0.000
NES = -2.15
PRE-REPLICATIVE COMPLEX FORMATION AND TRANSITION TO REPLICATION (MPM)
Nominal P-value = 0.000
FDR q-value = 0.000
NES = -2.12
PROTEASOME (KEGG)
Nominal P-value = 0.000
FDR q-value = 0.003
NES = -1.97
RIBOSOME (KEGG)
Nominal P-value = 0.000
FDR q-value = 0.003
NES = -1.96
PENTOSE PHOSPHATE PATHWAY (KEGG)
Nominal P-value = 0.000
FDR q-value = 0.003
NES = -1.92
REDOX METABOLISM (MPM)
Nominal P-value = 0.000
FDR q-value = 0.004
NES = -1.90
PROTEIN BIOSYNTHESIS (MPM)
Nominal P-value = 0.000
FDR q-value = 0.007
NES = -1.85
PYRIMIDINE METABOLISM (MPM)
Nominal P-value = 0.016
FDR q-value = 0.028
NES = -1.71
GLUTATHIONE METABOLISM (KEGG)
Nominal P-value = 0.011
FDR q-value = 0.030
NES = -1.70
GLUTAMATE METABOLISM (MPM)
Nominal P-value = 0.022
FDR q-value = 0.052
NES = -1.61
PRE-REPLICATIVE COMPLEX FORMATION AND TRANSITION TO REPLICATION (MPM)
Nominal P-value = 0.000
FDR q-value = 0.000
NES = -2.27
MITOCHONDRIAL TCA CYCLE (MPM)
Nominal P-value = 0.000
FDR q-value = 0.003
NES = -2.14
MITOCHONDRIAL ELECTRON FLOW (MPM)
Nominal P-value = 0.000
FDR q-value = 0.003
NES = -2.11
FUNCTIONAL ANNOTATION OF MEROZOITE INVASION-RELATED PROTEINS (MPM)
Nominal P-value = 0.000
FDR q-value = 0.036
NES = -1.81
TRANSLATION IN PROKARYOTES-A TEMPLATE FOR APICOPLAST AND MITOCHONDRION (MPM)
Nominal P-value = 0.003
FDR q-value = 0.036
NES = -1.81
