# Supplementary material for: Artemisinin resistance in Plasmodium falciparum is associated with an altered temporal pattern of transcription
Source: BMC Genomics. 2011 Aug 3;12:391. doi: 10.1186/1471-2164-12-391 (PMC3163569; doi:10.1186/1471-2164-12-391)
Supplement: Additional file 7 — Functional pathways with significant differential expression in rings, trophozoites and schizonts of artemisinin resistant and sensitive parasites. Gene sets (functional groups) which are denoted in the graphs are obtained from various data sets of previous studies [63,66,70] and plotted are all pathways that are significantly differentially expressed in artemisinin resistant parasites in at least one IDC stage. For each pathway, each data point at ring (14 hpi), trophozoite (26 hpi) and schizont (34 hpi) of the resistant (green triangle) and sensitive (blue diamond) series was calculated from taking the average of the expression log2 ratio of a gene across all isolates in that phenotypic group (resistant or sensitive) and then averaged for all the genes associated with that pathway. Best fit polynomial curves were plotted (lines). Error bars indicate the standard deviation among the isolates for that pathway. Included separately are the average expression ratios of all genes belonging to that pathway for CP022 isolate (purple circle) from Pailin, Cambodia and reference in vitro strain (red square). [file 1471-2164-12-391-S7.PDF]

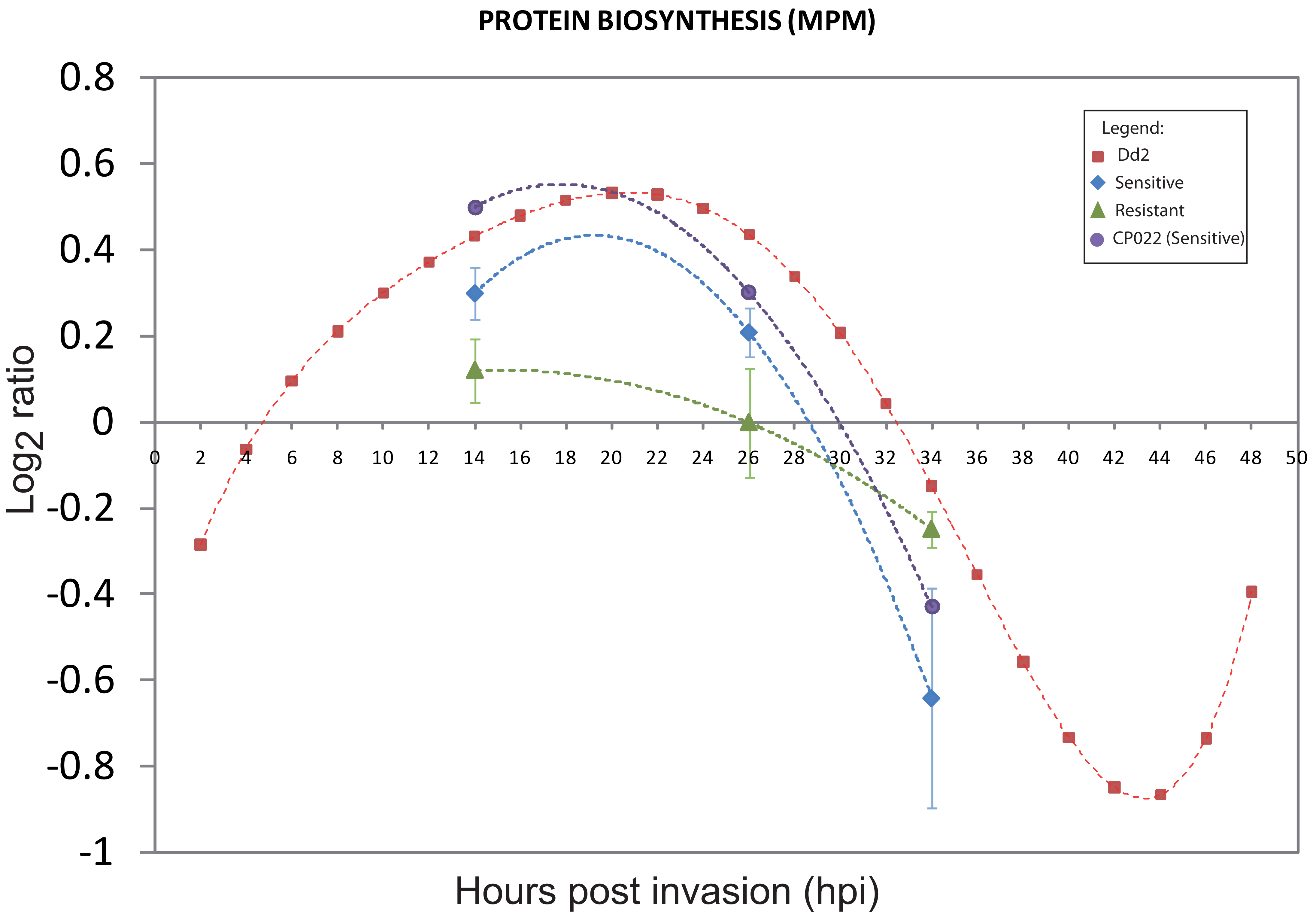
PROTEIN BIOSYNTHESIS (MPM)
Legend:
Dd2
Sensitive
Resistant
CP022 (Sensitive)
0.8
0.6
0.4
0.2
0
-0.2
-0.4
-0.6
-0.8
-1
0 2 4 6 8 10 12 14 16 18 20 22 24 26 28 30 32 34 36 38 40 42 44 46 48 50
Log2 ratio
Hours post invasion (hpi)

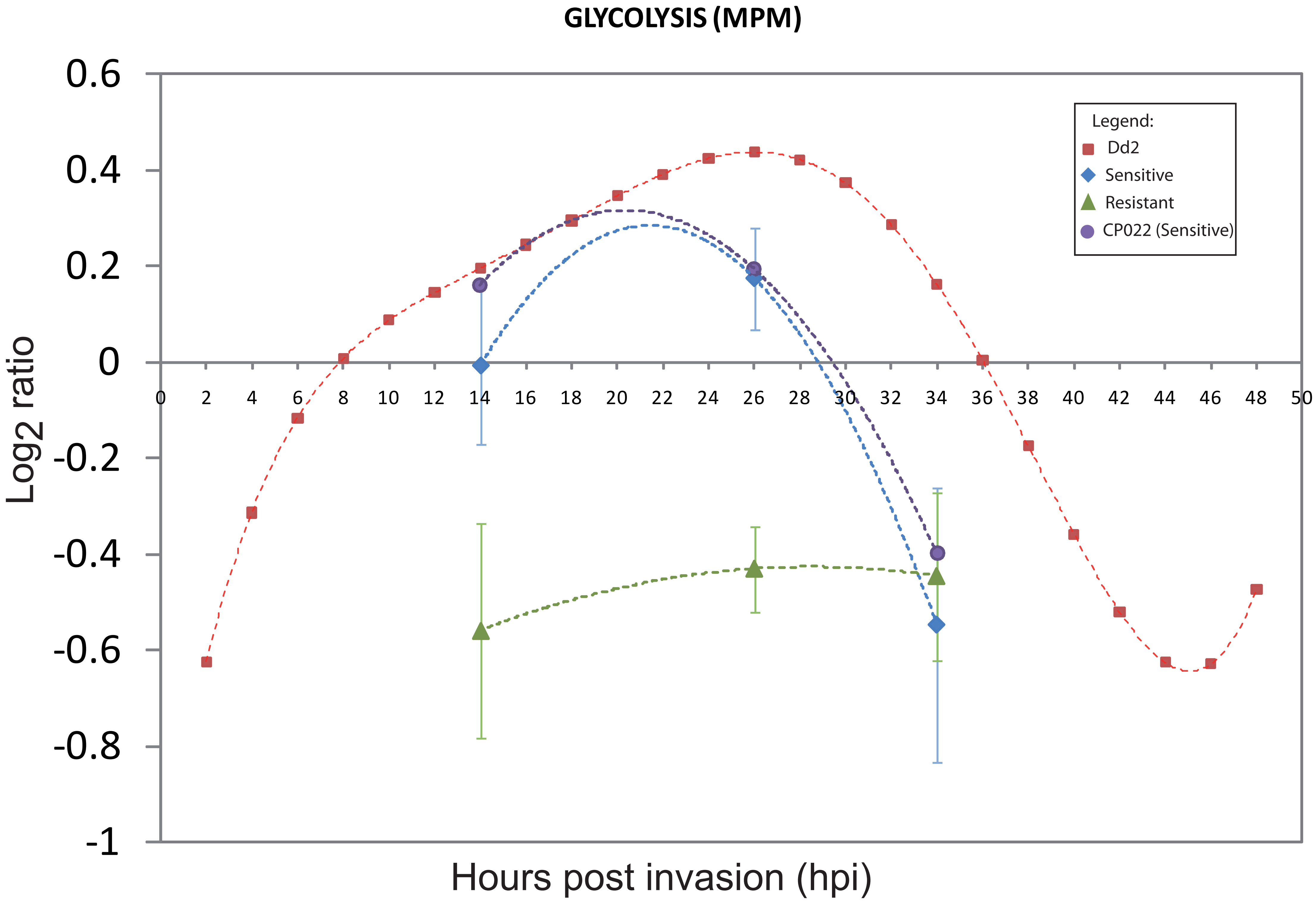
GLYCOLYSIS (MPM)
Legend:
Dd2
Sensitive
Resistant
CP022 (Sensitive)
Log2 ratio
Hours post invasion (hpi)
0.6
0.4
0.2
0
-0.2
-0.4
-0.6
-0.8
-1
0 2 4 6 8 10 12 14 16 18 20 22 24 26 28 30 32 34 36 38 40 42 44 46 48 50

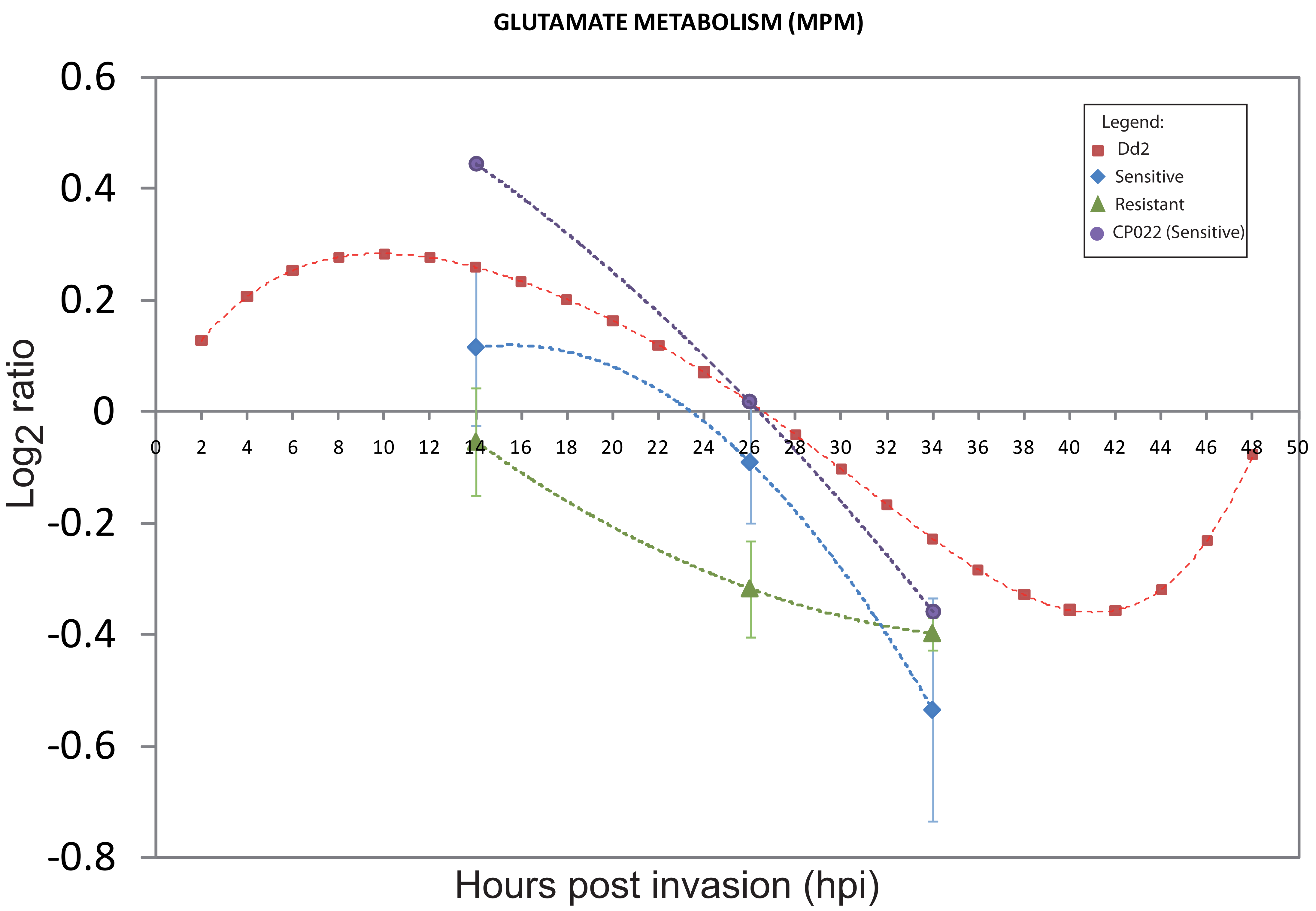
GLUTAMATE METABOLISM (MPM)
Legend:
Dd2
Sensitive
Resistant
CP022 (Sensitive)
Log2 ratio
0.6
0.4
0.2
0
-0.2
-0.4
-0.6
-0.8
0 2 4 6 8 10 12 14 16 18 20 22 24 26 28 30 32 34 36 38 40 42 44 46 48 50
Hours post invasion (hpi)

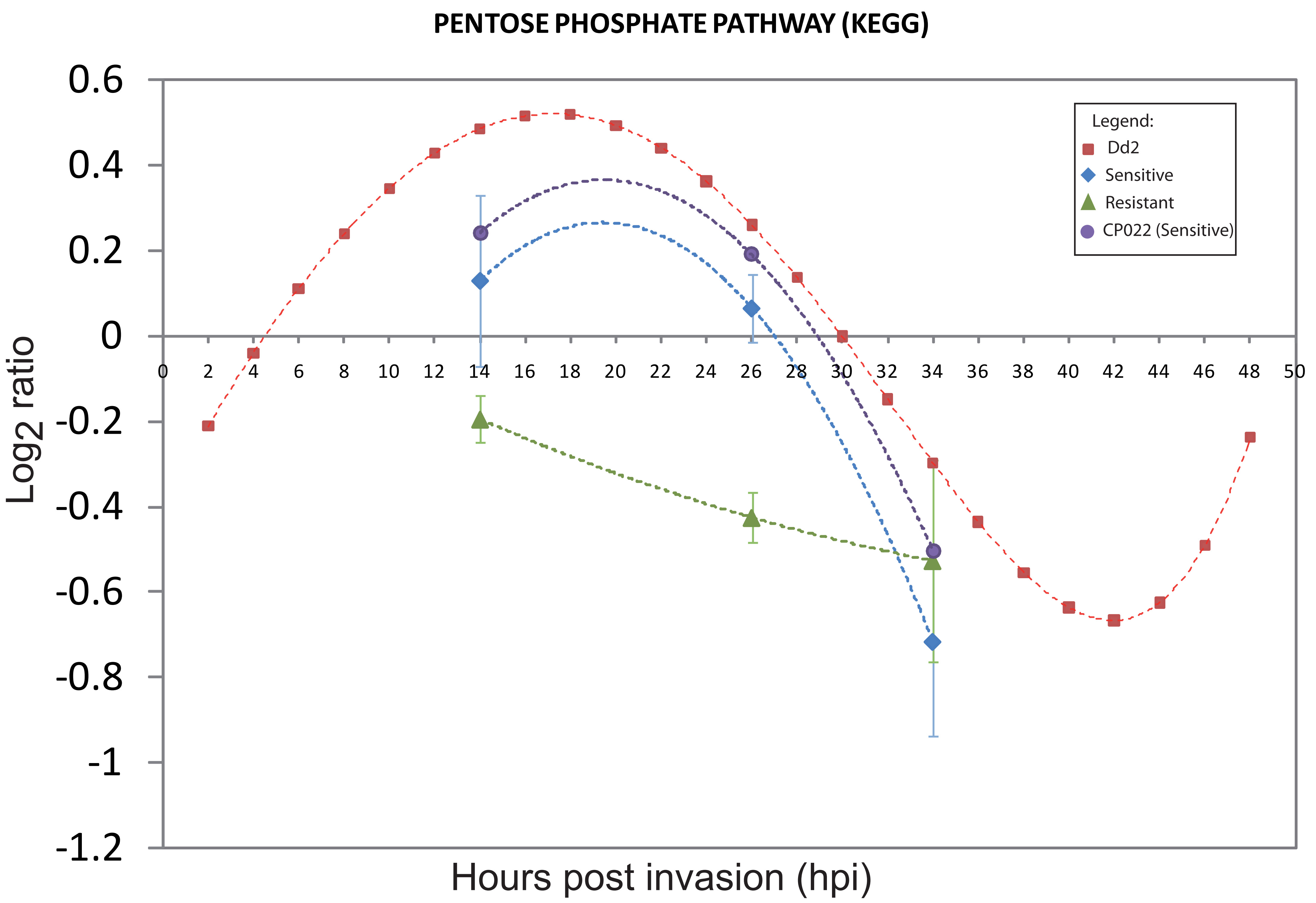
PENTOSE PHOSPHATE PATHWAY (KEGG)
0.6
0.4
0.2
0
-0.2
-0.4
-0.6
-0.8
-1
-1.2
Log2 ratio
0 2 4 6 8 10 12 14 16 18 20 22 24 26 28 30 32 34 36 38 40 42 44 46 48 50
Hours post invasion (hpi)
Legend:
Dd2
Sensitive
Resistant
CP022 (Sensitive)

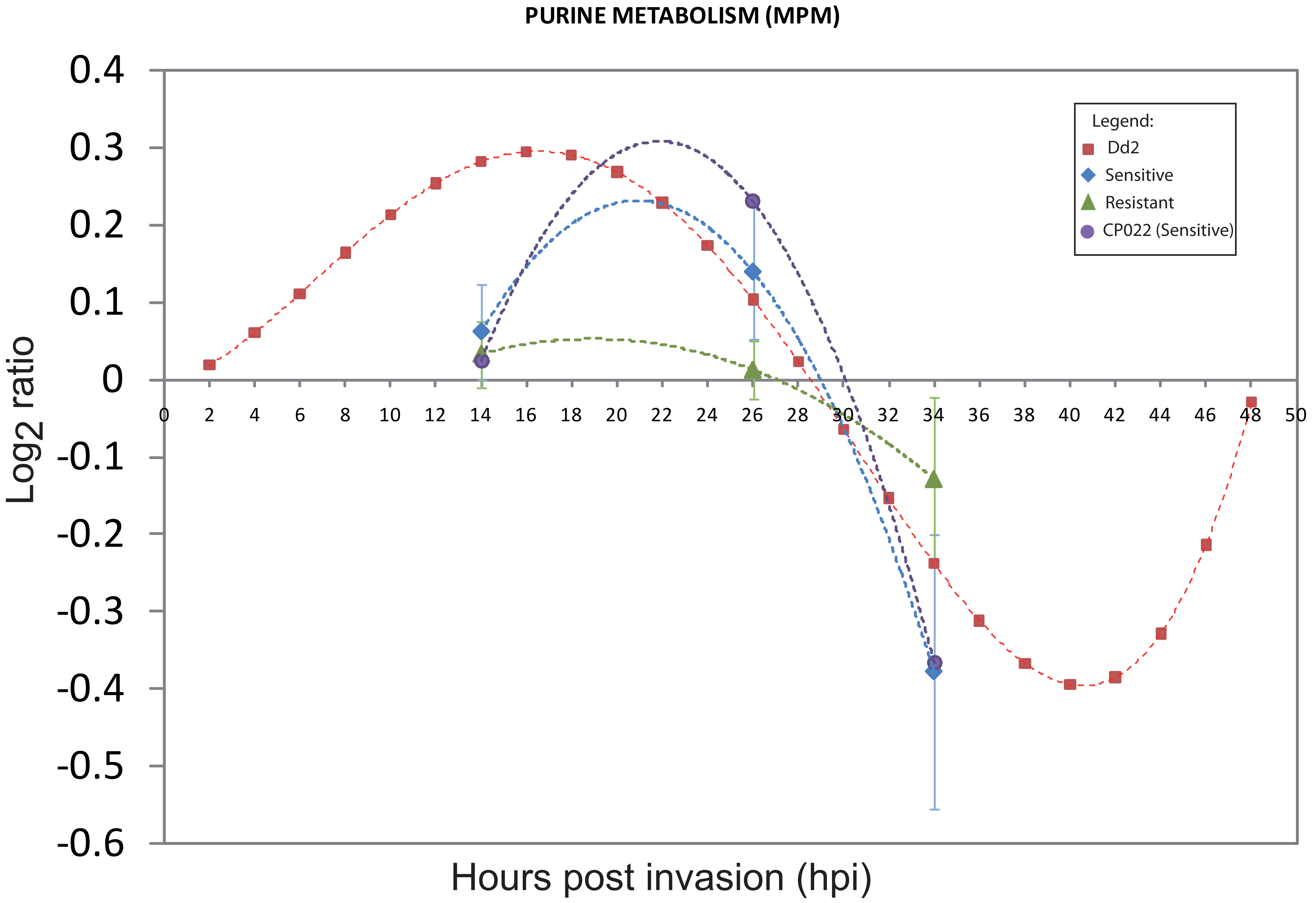

PURINE METABOLISM (MPM)
Legend:
Dd2
Sensitive
Resistant
CP022 (Sensitive)
0.4
0.3
0.2
0.1
0
-0.1
-0.2
-0.3
-0.4
-0.5
-0.6
0 2 4 6 8 10 12 14 16 18 20 22 24 26 28 30 32 34 36 38 40 42 44 46 48 50
Log2 ratio
Hours post invasion (hpi)

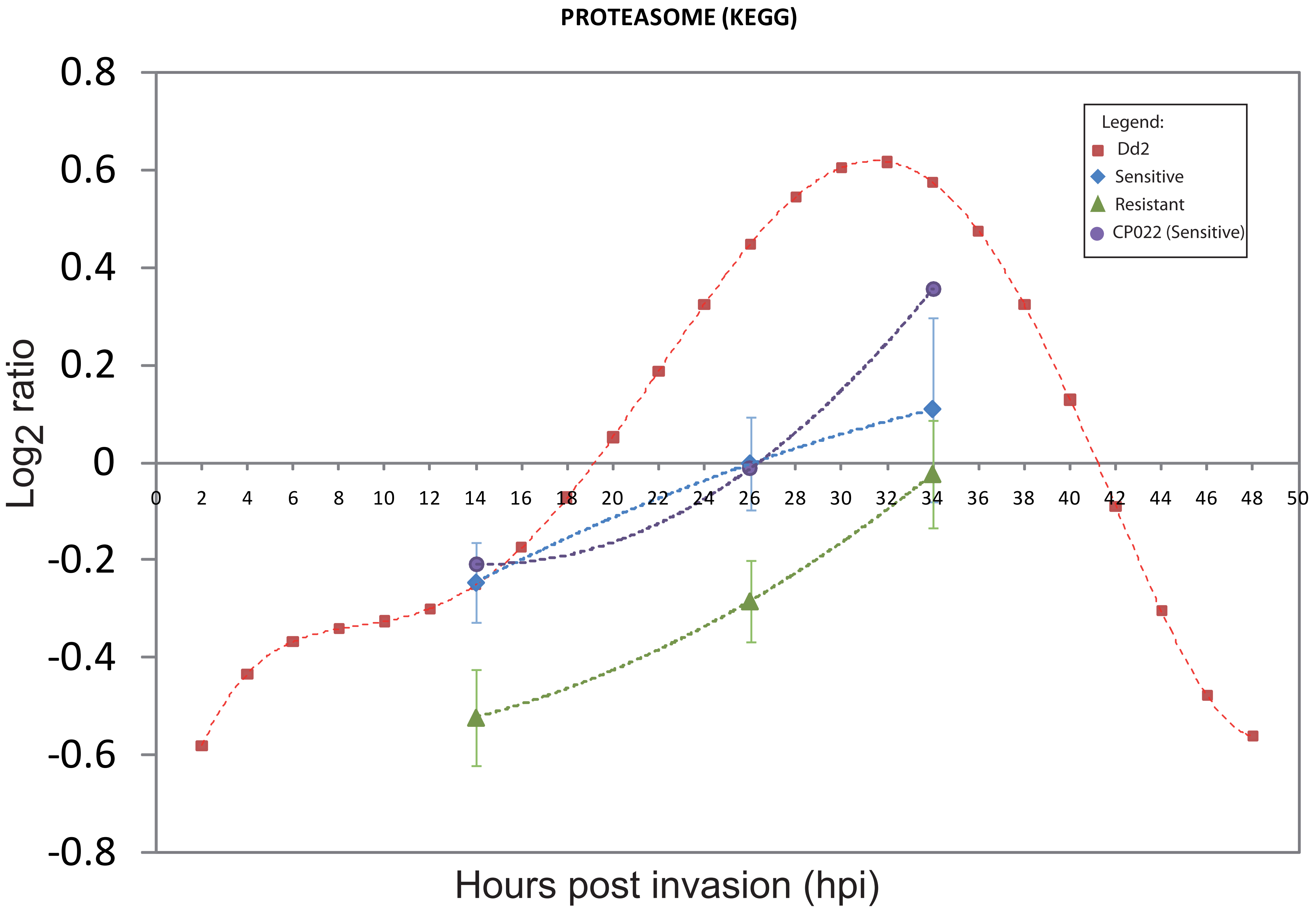

PROTEASOME (KEGG)
0.8
0.6
0.4
0.2
0
-0.2
-0.4
-0.6
-0.8
Log2 ratio
0 2 4 6 8 10 12 14 16 18 20 22 24 26 28 30 32 34 36 38 40 42 44 46 48 50
Hours post invasion (hpi)
Legend:
Dd2
Sensitive
Resistant
CP022 (Sensitive)

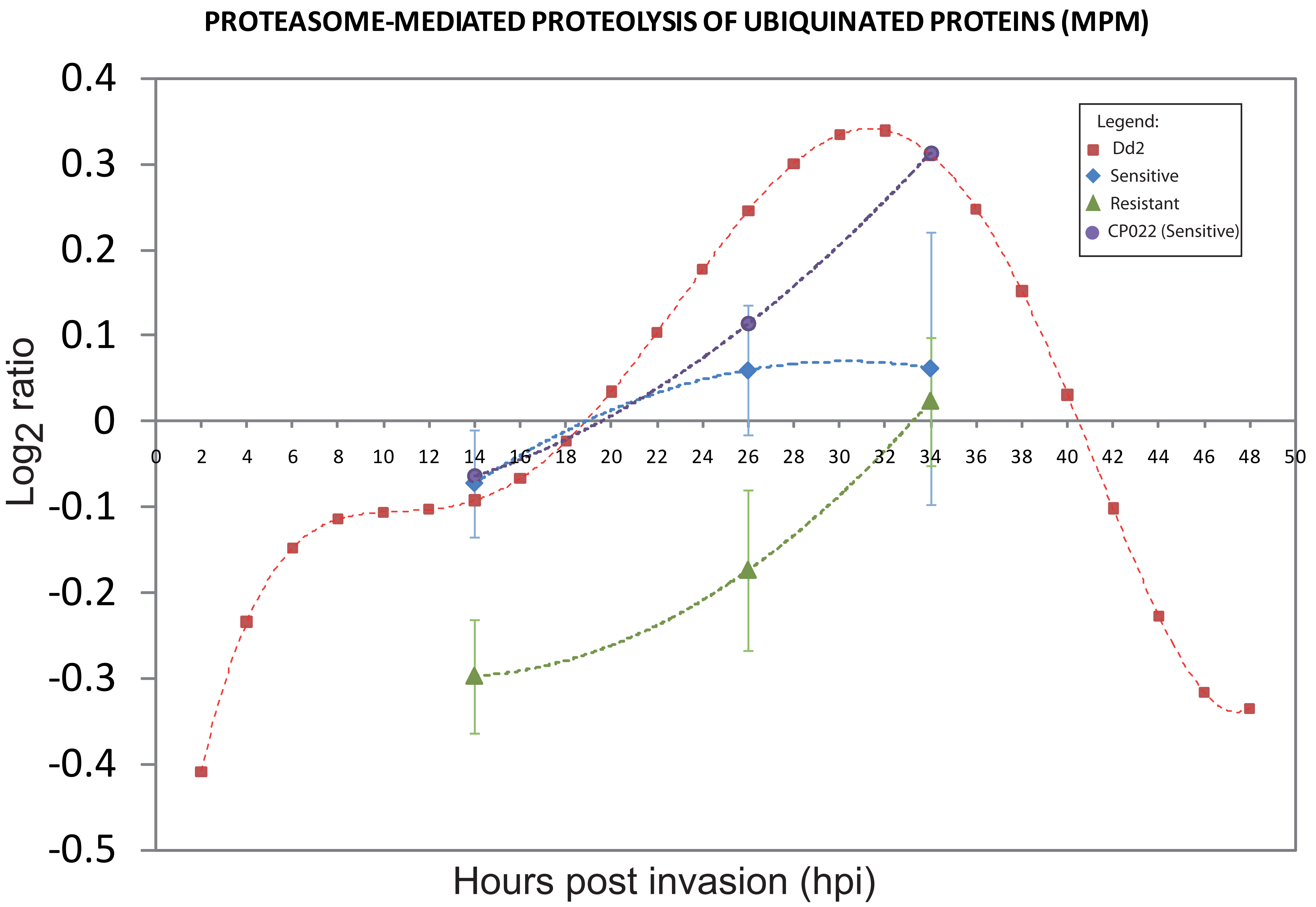
PROTEASOME-MEDIATED PROTEOLYSIS OF UBIQUINATED PROTEINS (MPM)
Legend:
Dd2
Sensitive
Resistant
CP022 (Sensitive)
0.4
0.3
0.2
0.1
0
-0.1
-0.2
-0.3
-0.4
-0.5
0 2 4 6 8 10 12 14 16 18 20 22 24 26 28 30 32 34 36 38 40 42 44 46 48 50
Log2 ratio
Hours post invasion (hpi)

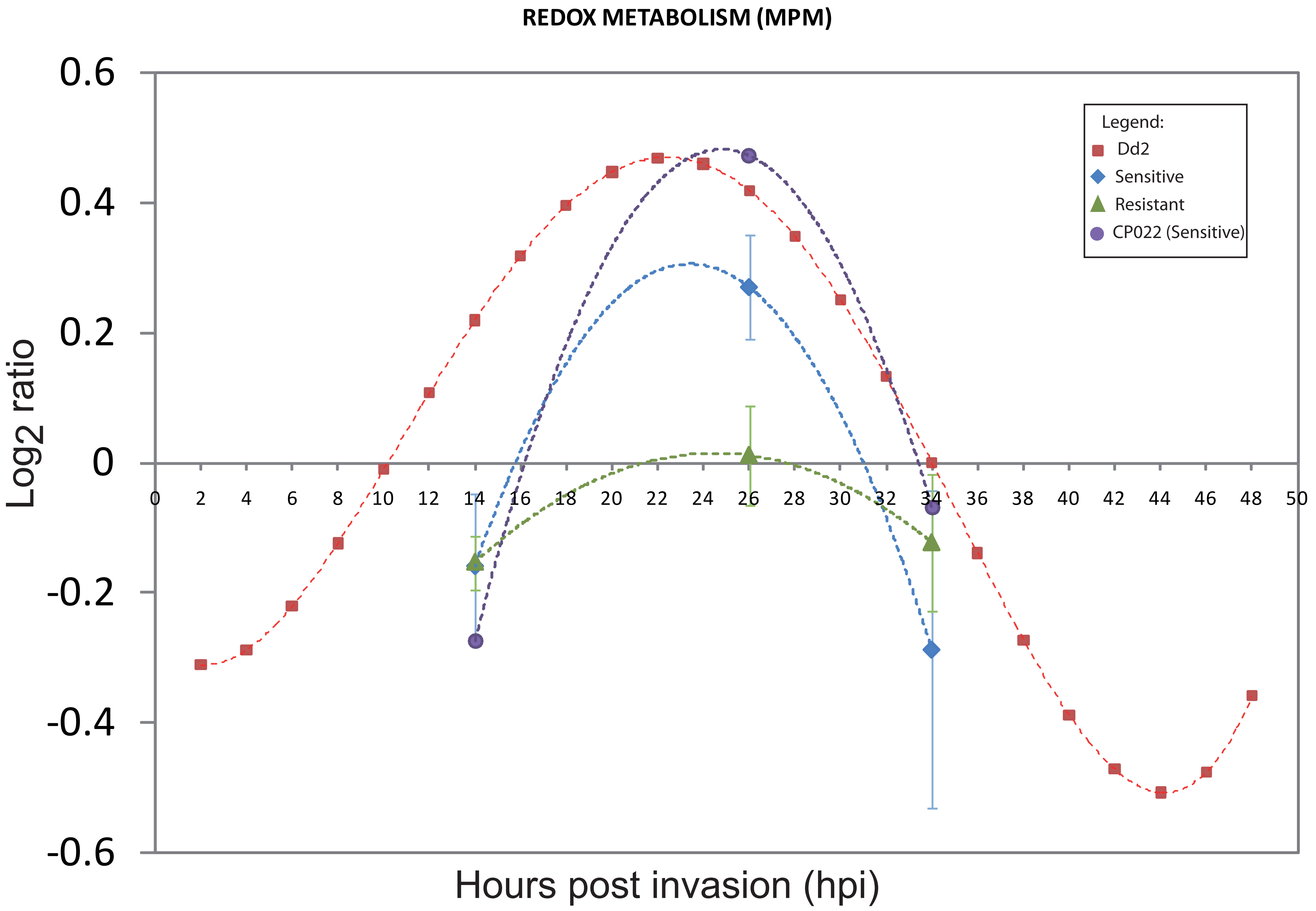
REDOX METABOLISM (MPM)
0.6
0.4
0.2
0
-0.2
-0.4
-0.6
Log2 ratio
0 2 4 6 8 10 12 14 16 18 20 22 24 26 28 30 32 34 36 38 40 42 44 46 48 50
Hours post invasion (hpi)
Legend:
Dd2
Sensitive
Resistant
CP022 (Sensitive)

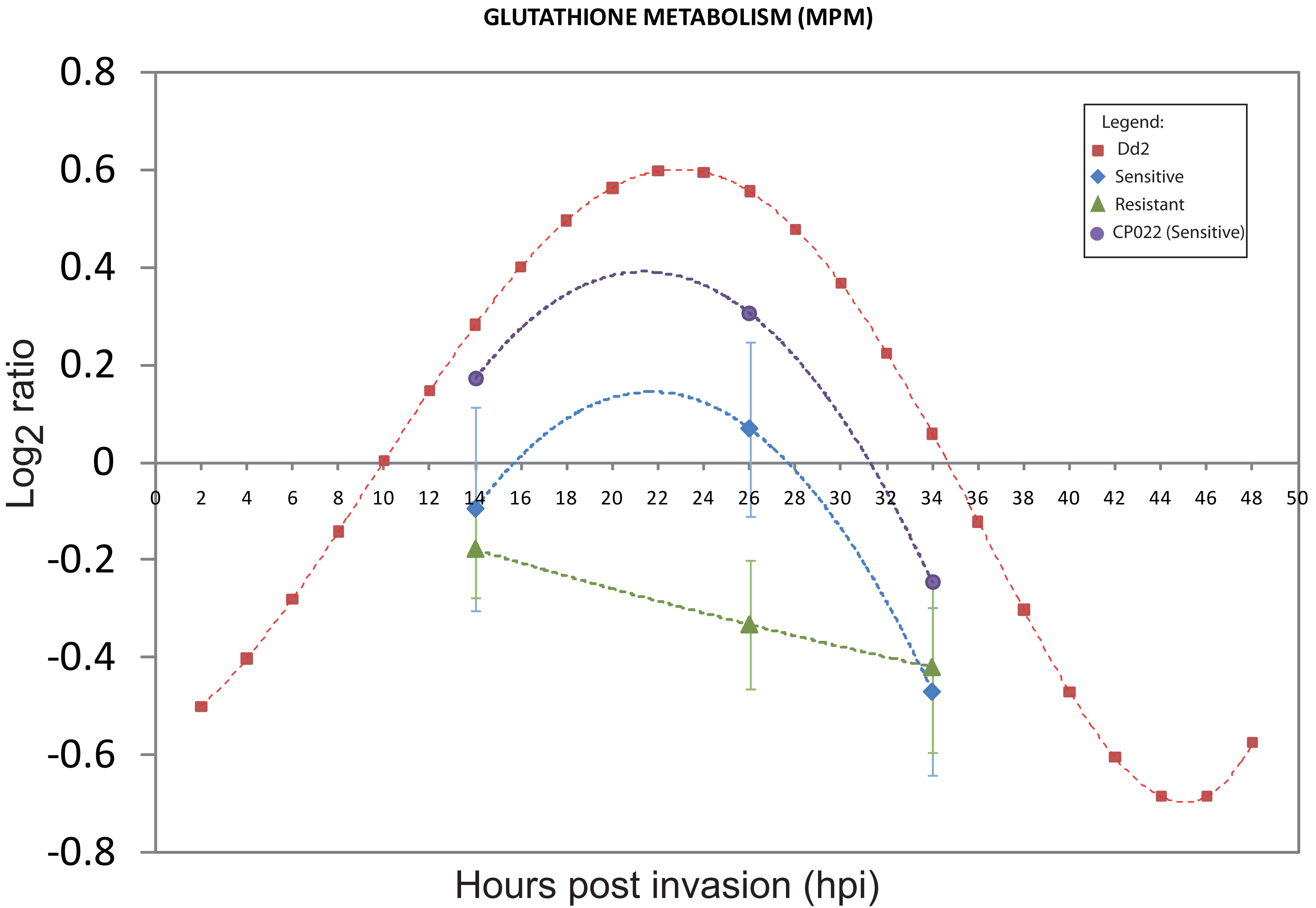
GLUTATHIONE METABOLISM (MPM)
Legend:
Dd2
Sensitive
Resistant
CP022 (Sensitive)
Log2 ratio
0.8
0.6
0.4
0.2
0
-0.2
-0.4
-0.6
-0.8
0 2 4 6 8 10 12 14 16 18 20 22 24 26 28 30 32 34 36 38 40 42 44 46 48 50
Hours post invasion (hpi)

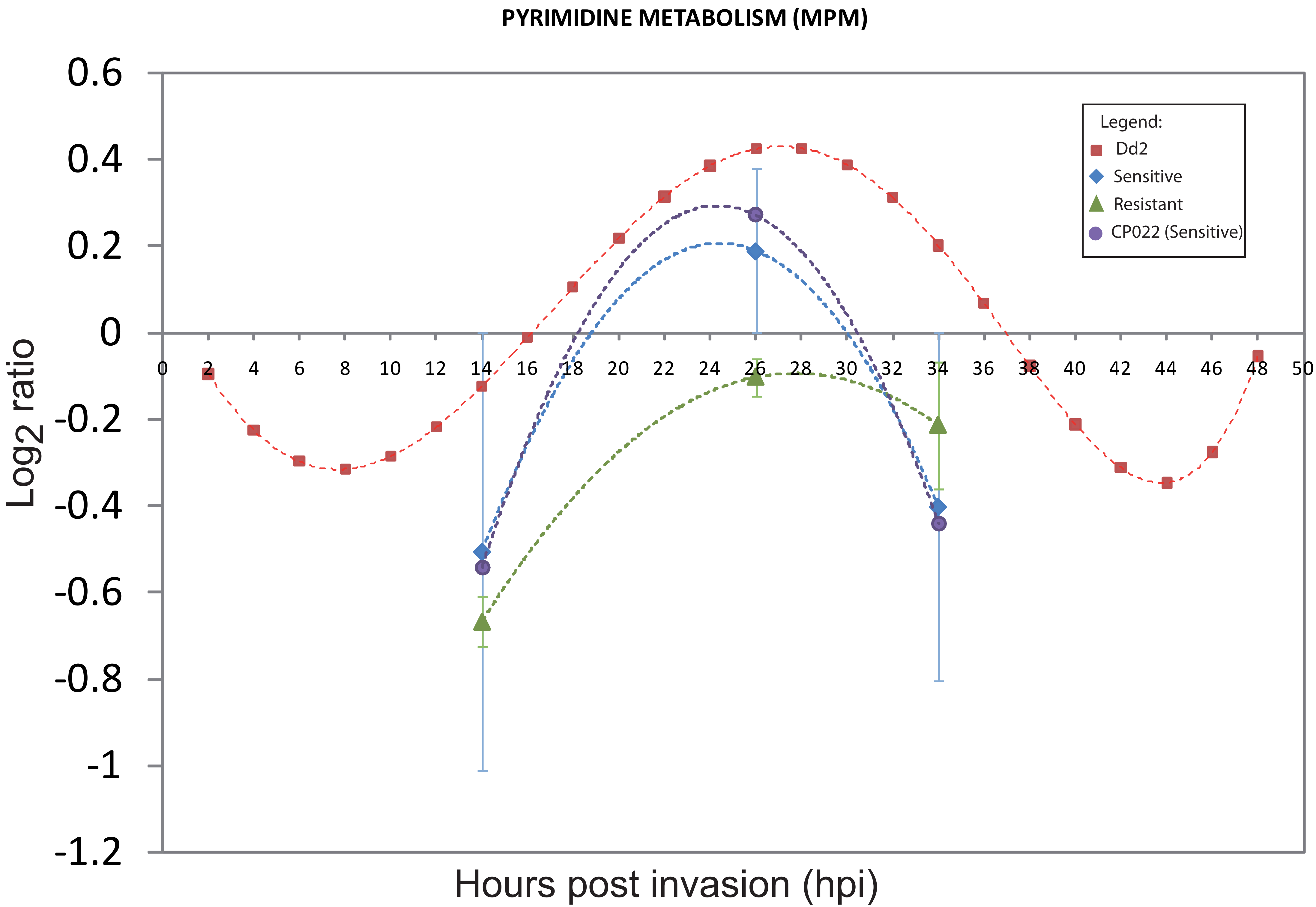
PYRIMIDINE METABOLISM (MPM)
Legend:
Dd2
Sensitive
Resistant
CP022 (Sensitive)
Log2 ratio
0.6
0.4
0.2
0
-0.2
-0.4
-0.6
-0.8
-1
-1.2
0 2 4 6 8 10 12 14 16 18 20 22 24 26 28 30 32 34 36 38 40 42 44 46 48 50
Hours post invasion (hpi)

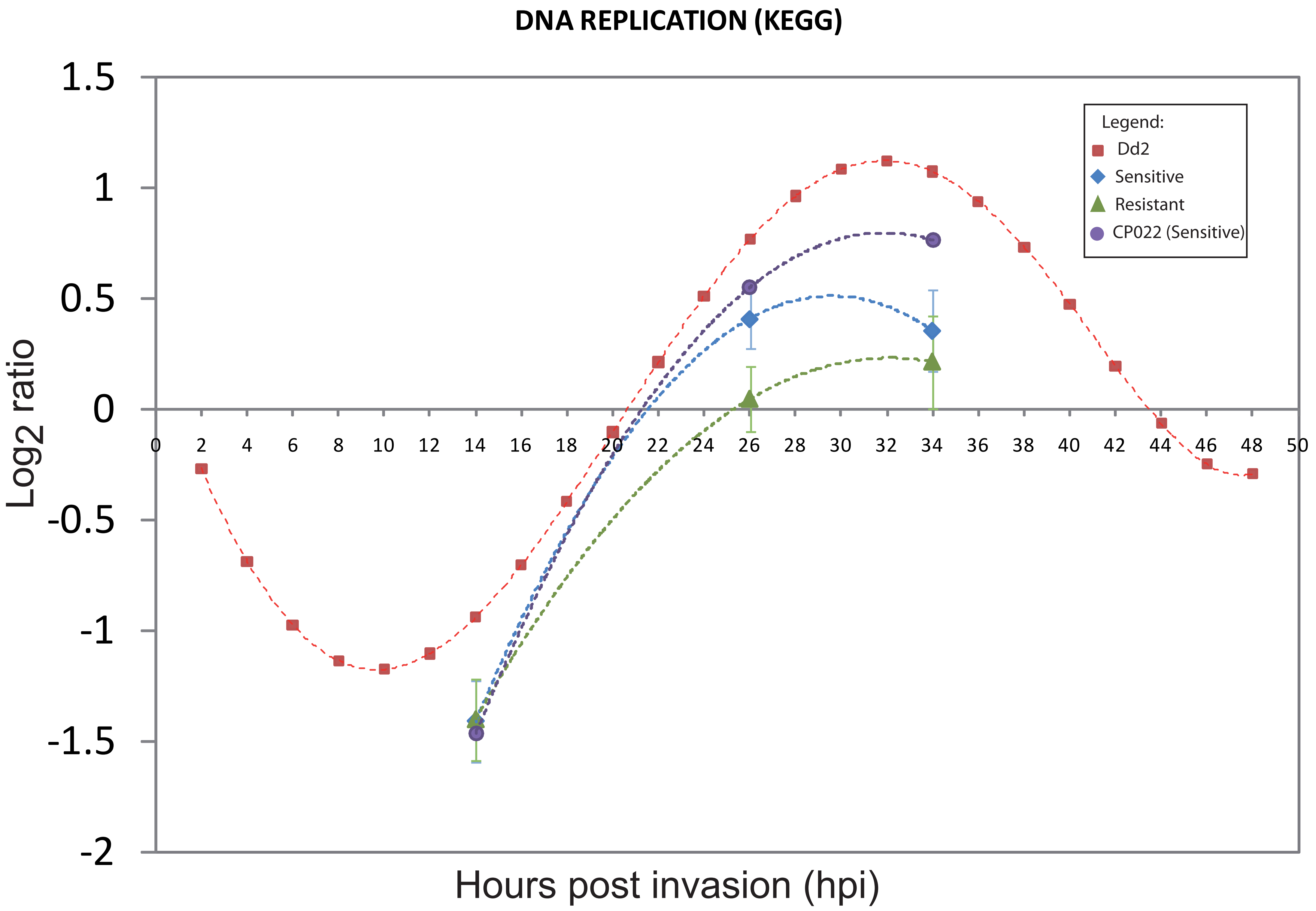
DNA REPLICATION (KEGG)
Legend:
Dd2
Sensitive
Resistant
CP022 (Sensitive)
1.5
1
0.5
0
-0.5
-1
-1.5
-2
Log2 ratio
0 2 4 6 8 10 12 14 16 18 20 22 24 26 28 30 32 34 36 38 40 42 44 46 48 50
Hours post invasion (hpi)

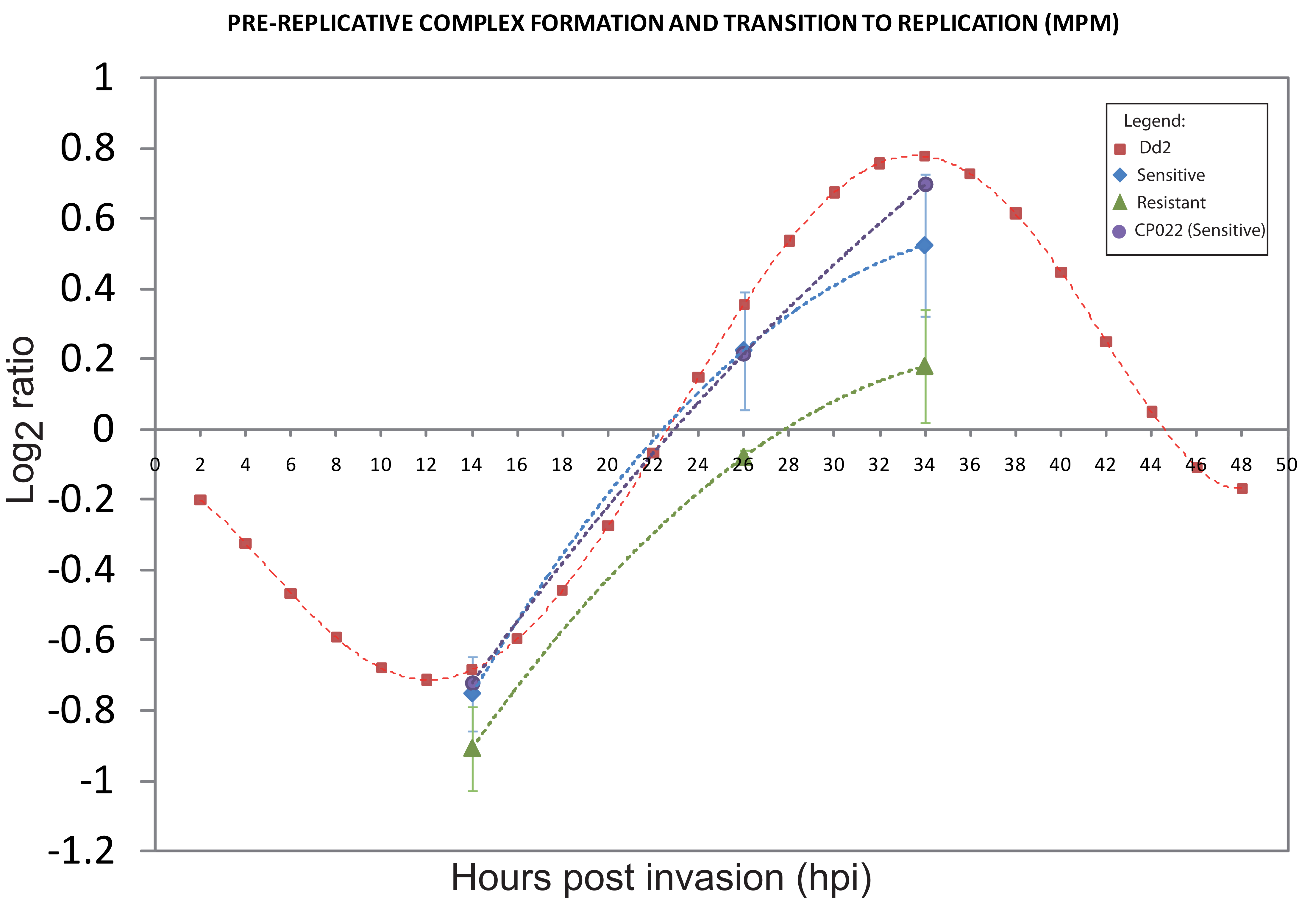
PRE-REPLICATIVE COMPLEX FORMATION AND TRANSITION TO REPLICATION (MPM)
Log2 ratio
Hours post invasion (hpi)
Legend:
Dd2
Sensitive
Resistant
CP022 (Sensitive)
1
0.8
0.6
0.4
0.2
0
-0.2
-0.4
-0.6
-0.8
-1
-1.2
0 2 4 6 8 10 12 14 16 18 20 22 24 26 28 30 32 34 36 38 40 42 44 46 48 50

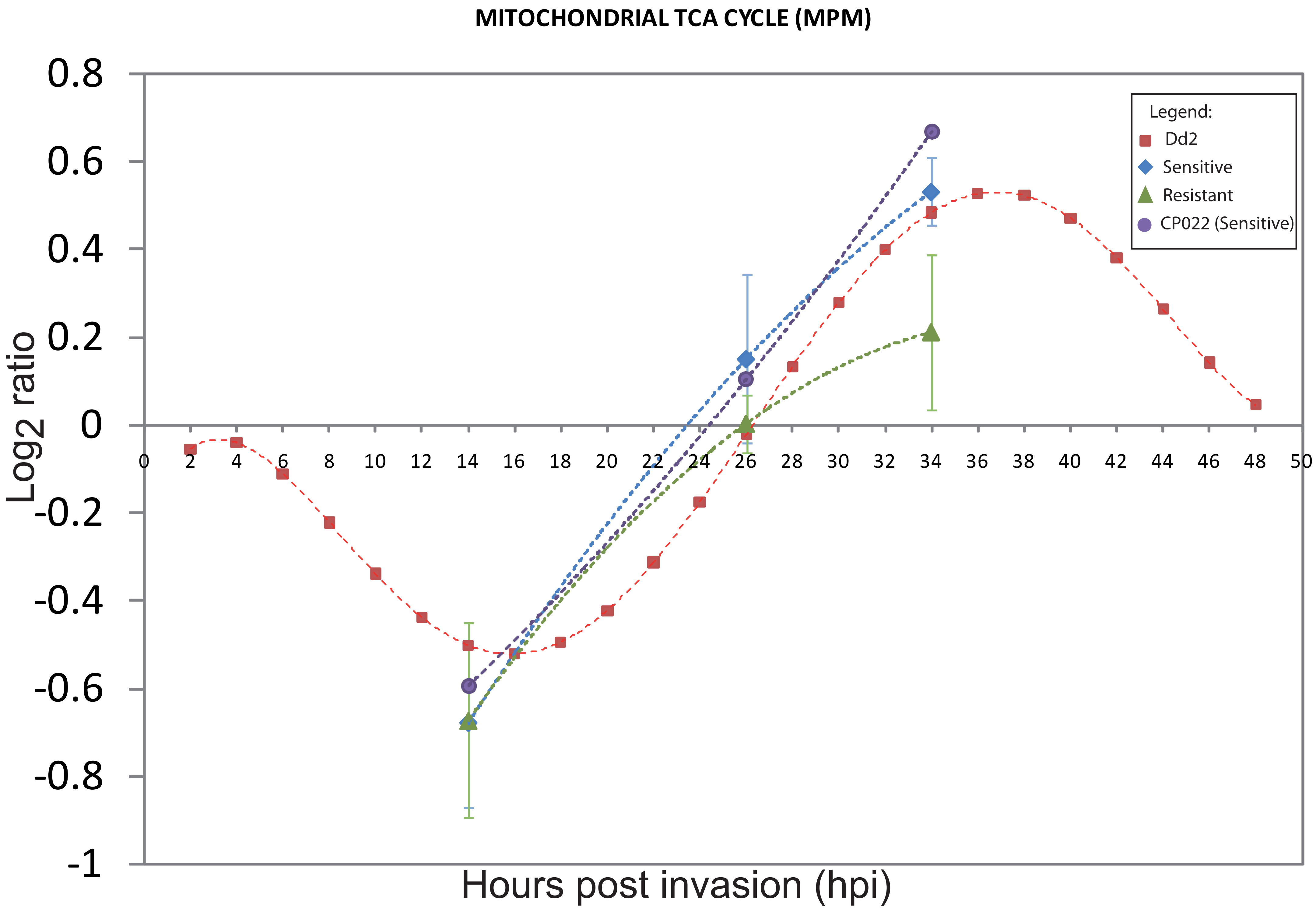
MITOCHONDRIAL TCA CYCLE (MPM)
Log2 ratio
Hours post invasion (hpi)
Legend:
Dd2
Sensitive
Resistant
CP022 (Sensitive)
0.8
0.6
0.4
0.2
0
-0.2
-0.4
-0.6
-0.8
-1
0 2 4 6 8 10 12 14 16 18 20 22 24 26 28 30 32 34 36 38 40 42 44 46 48 50

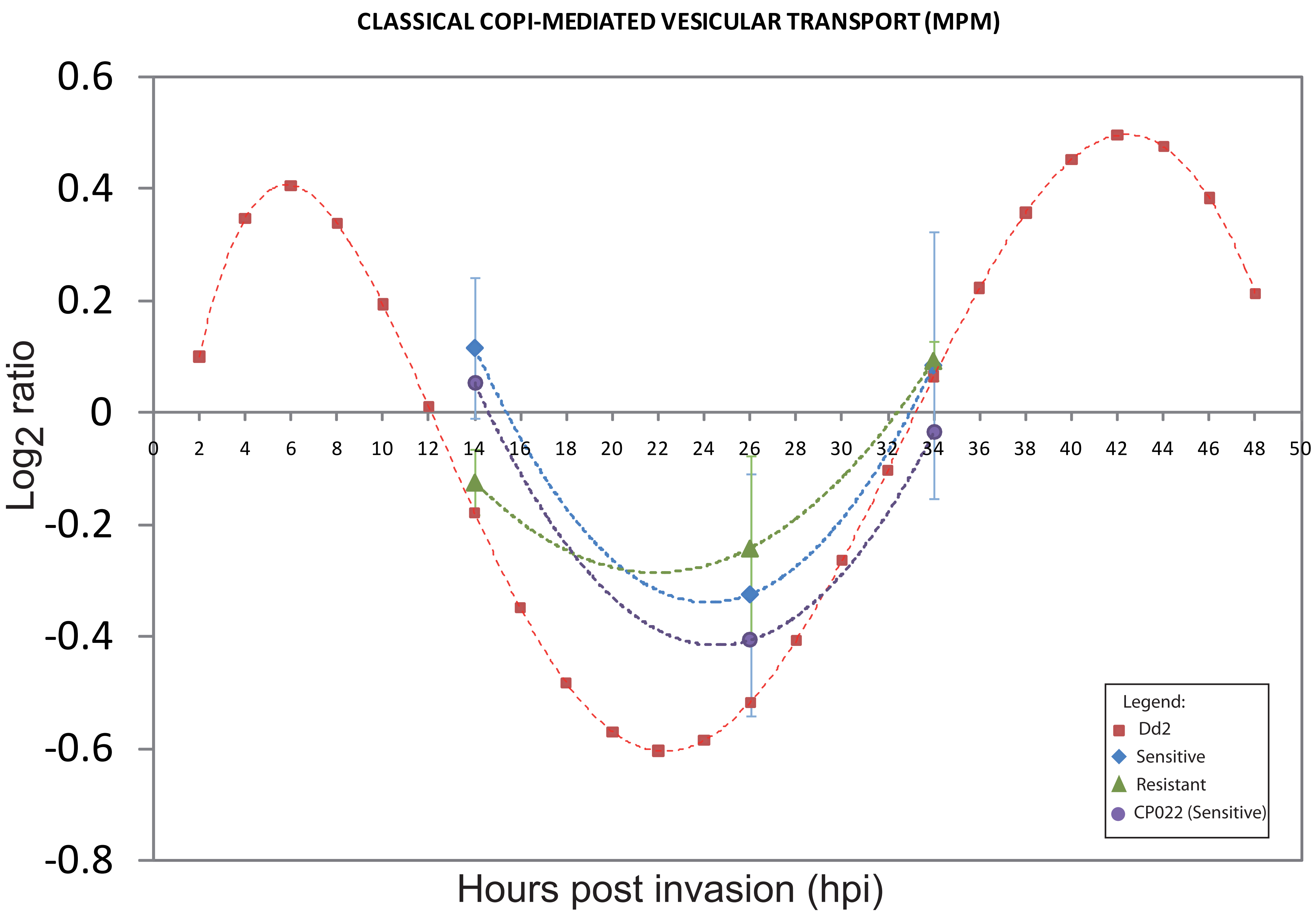
CLASSICAL COPI-MEDIATED VESICULAR TRANSPORT (MPM)
0.6
0.4
0.2
0
-0.2
-0.4
-0.6
-0.8
Log2 ratio
0 2 4 6 8 10 12 14 16 18 20 22 24 26 28 30 32 34 36 38 40 42 44 46 48 50
Hours post invasion (hpi)
Legend:
Dd2
Sensitive
Resistant
CP022 (Sensitive)

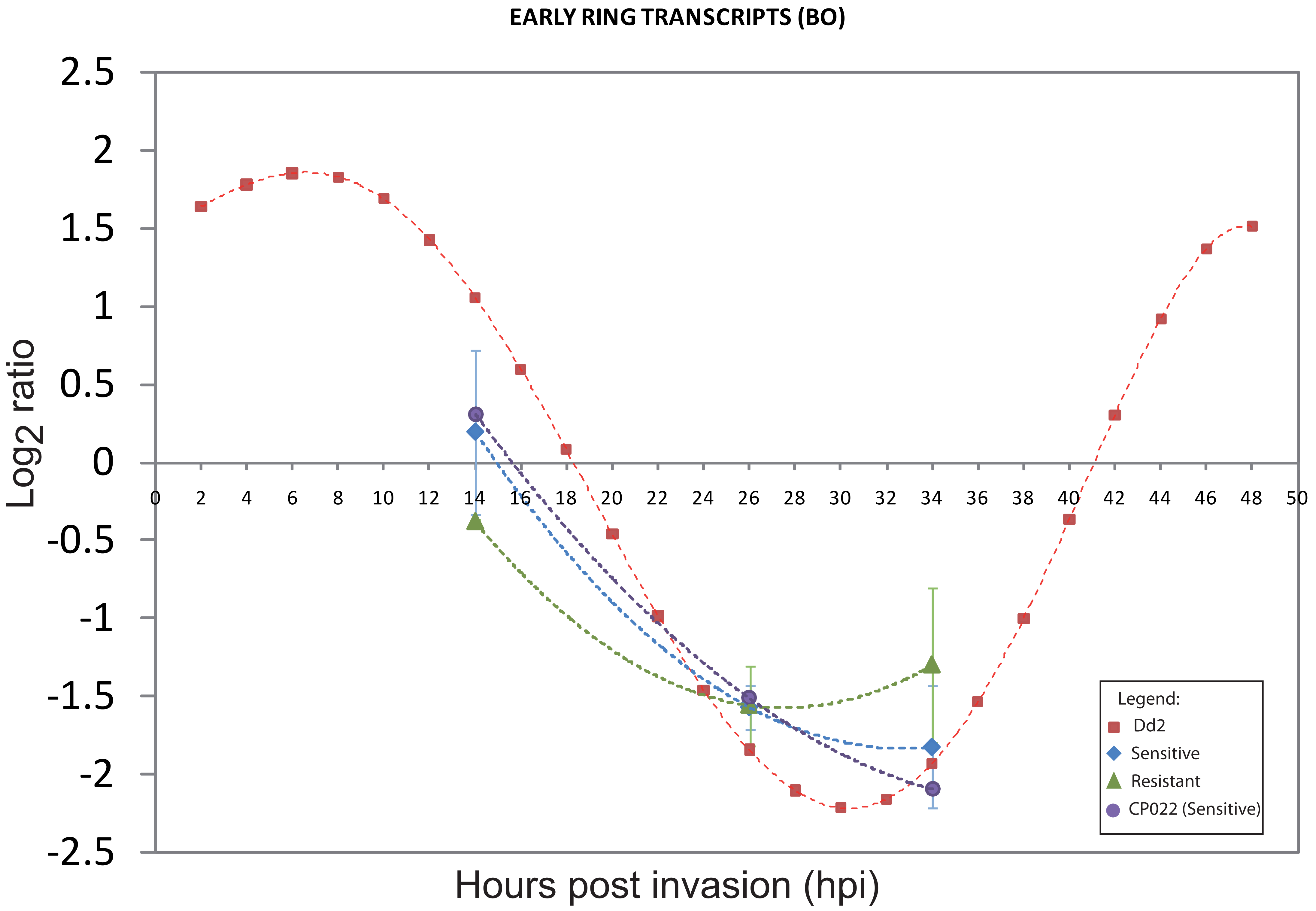
EARLY RING TRANSCRIPTS (BO)
2.5
2
1.5
1
0.5
0
-0.5
-1
-1.5
-2
-2.5
Log2 ratio
0 2 4 6 8 10 12 14 16 18 20 22 24 26 28 30 32 34 36 38 40 42 44 46 48 50
Hours post invasion (hpi)
Legend:
Dd2
Sensitive
Resistant
CP022 (Sensitive)

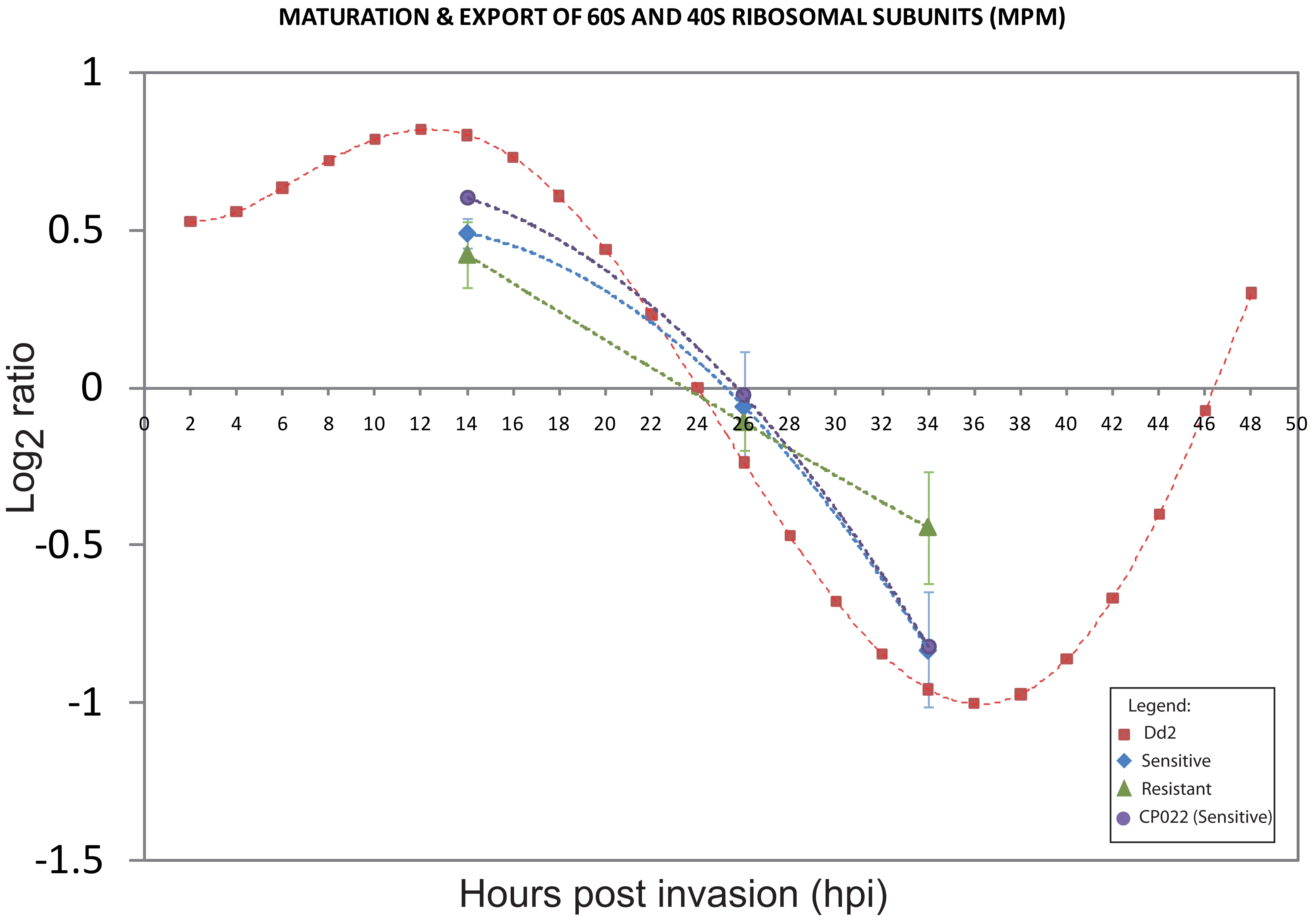

MATURATION & EXPORT OF 60S AND 40S RIBOSOMAL SUBUNITS (MPM)
1
0.5
0
-0.5
-1
-1.5
Log2 ratio
0 2 4 6 8 10 12 14 16 18 20 22 24 26 28 30 32 34 36 38 40 42 44 46 48 50
Hours post invasion (hpi)
Legend:
Dd2
Sensitive
Resistant
CP022 (Sensitive)

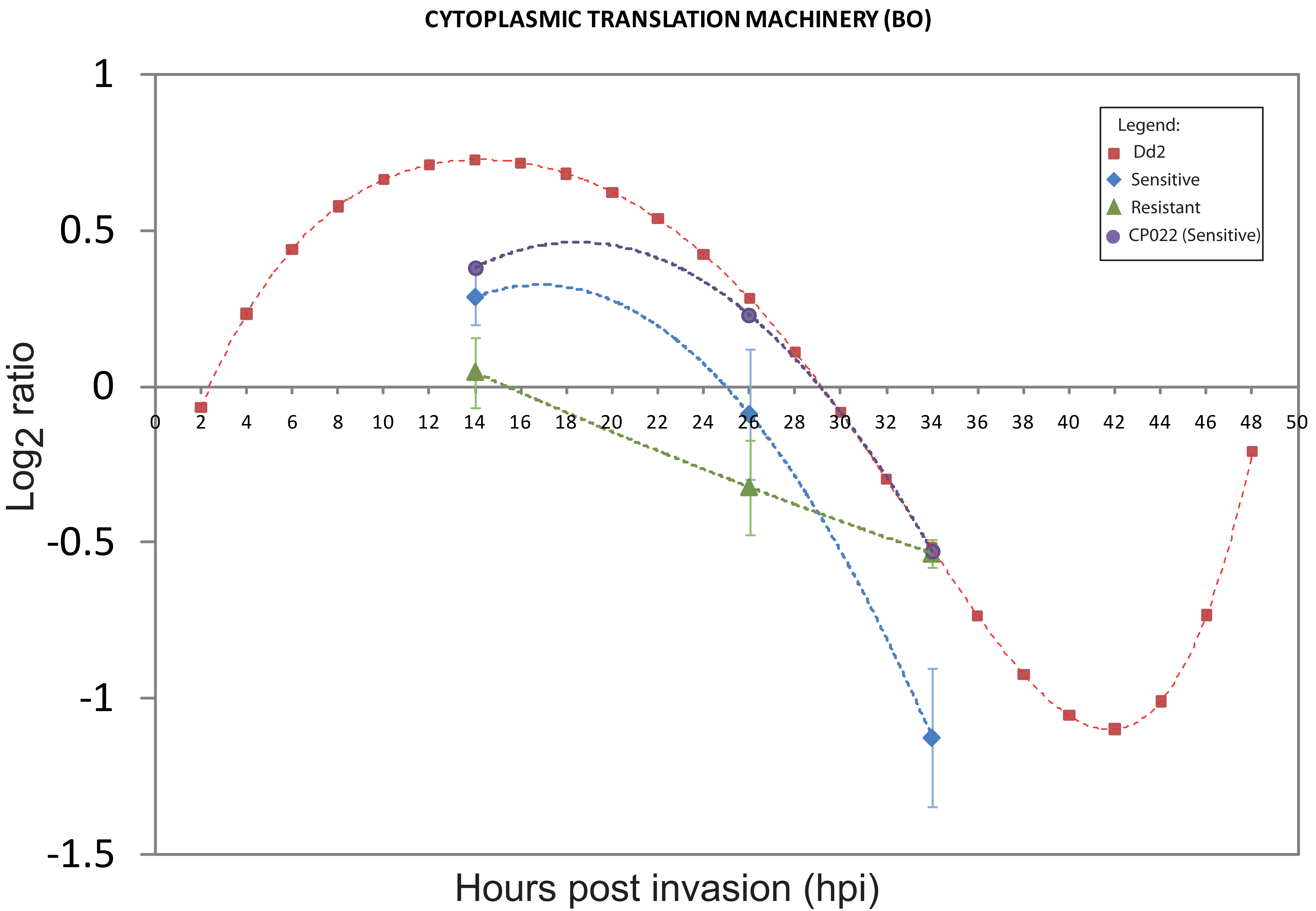

CYTOPLASMIC TRANSLATION MACHINERY (BO)
Legend:
Dd2
Sensitive
Resistant
CP022 (Sensitive)
1
0.5
0
-0.5
-1
-1.5
Log2 ratio
0 2 4 6 8 10 12 14 16 18 20 22 24 26 28 30 32 34 36 38 40 42 44 46 48 50
Hours post invasion (hpi)

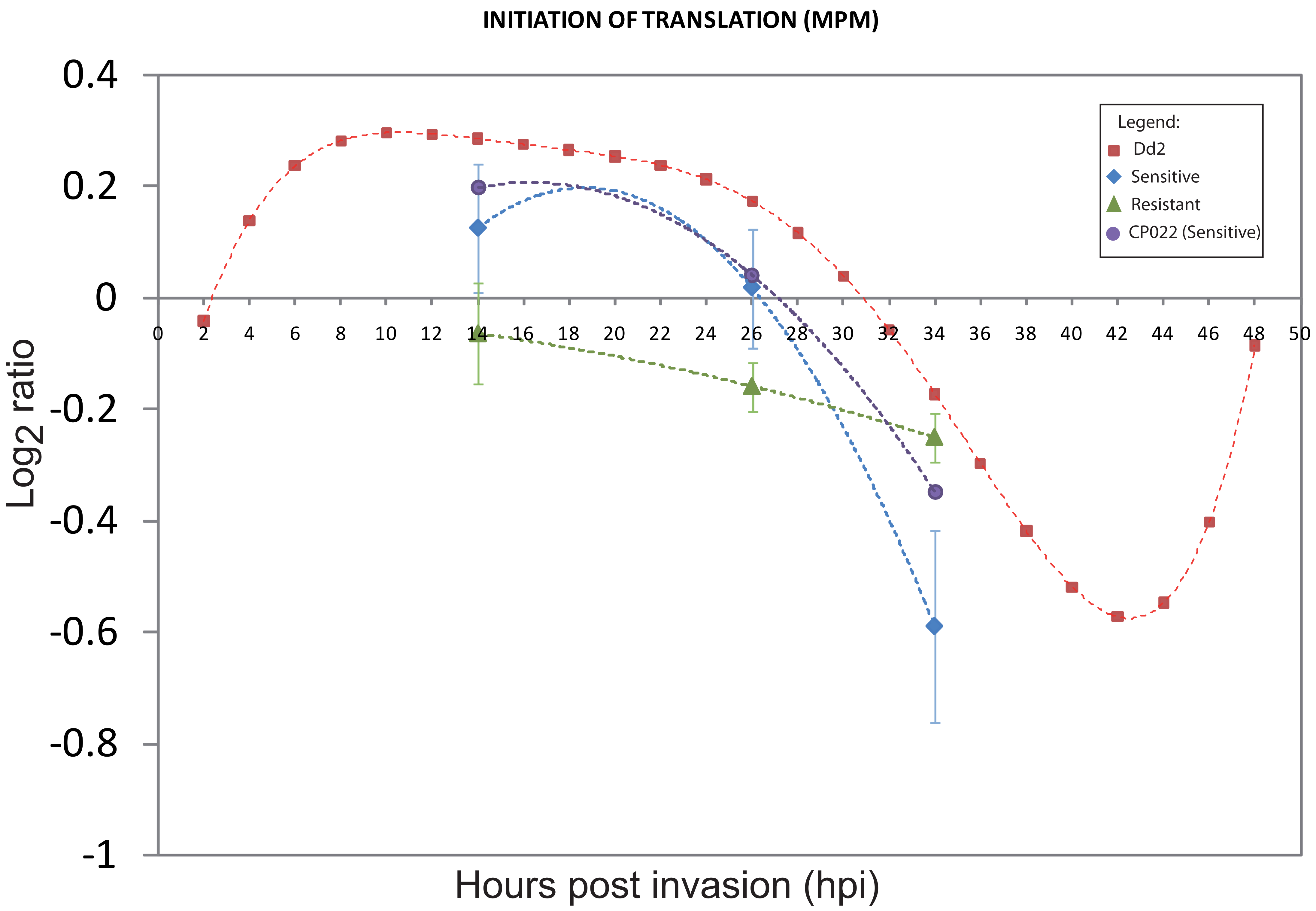

INITIATION OF TRANSLATION (MPM)
Legend:
Dd2
Sensitive
Resistant
CP022 (Sensitive)
Log2 ratio
0.4
0.2
0
-0.2
-0.4
-0.6
-0.8
-1
0 2 4 6 8 10 12 14 16 18 20 22 24 26 28 30 32 34 36 38 40 42 44 46 48 50
Hours post invasion (hpi)

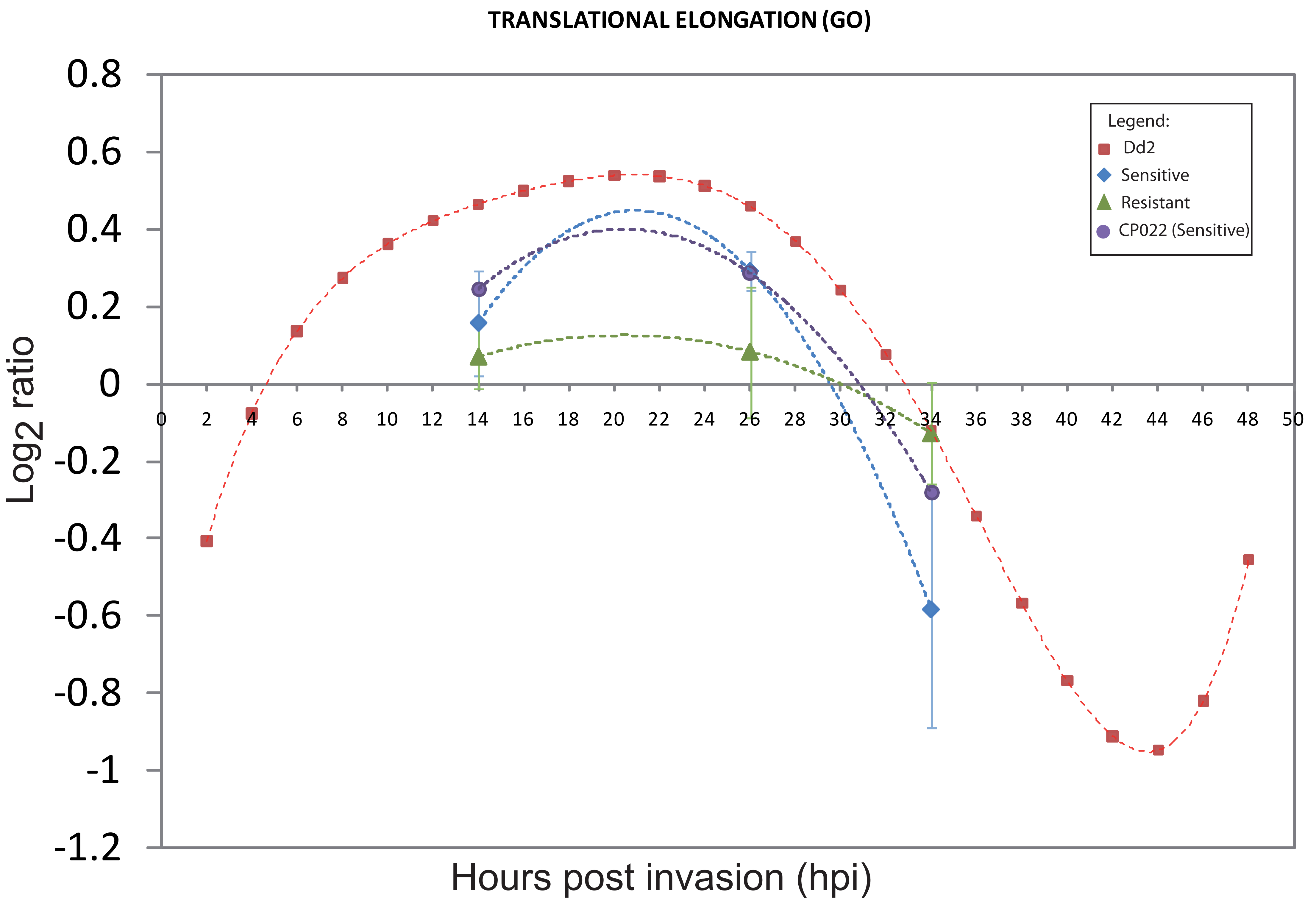

TRANSLATIONAL ELONGATION (GO)
Legend:
Dd2
Sensitive
Resistant
CP022 (Sensitive)
0.8
0.6
0.4
0.2
0
-0.2
-0.4
-0.6
-0.8
-1
-1.2
0 2 4 6 8 10 12 14 16 18 20 22 24 26 28 30 32 34 36 38 40 42 44 46 48 50
Log2 ratio
Hours post invasion (hpi)

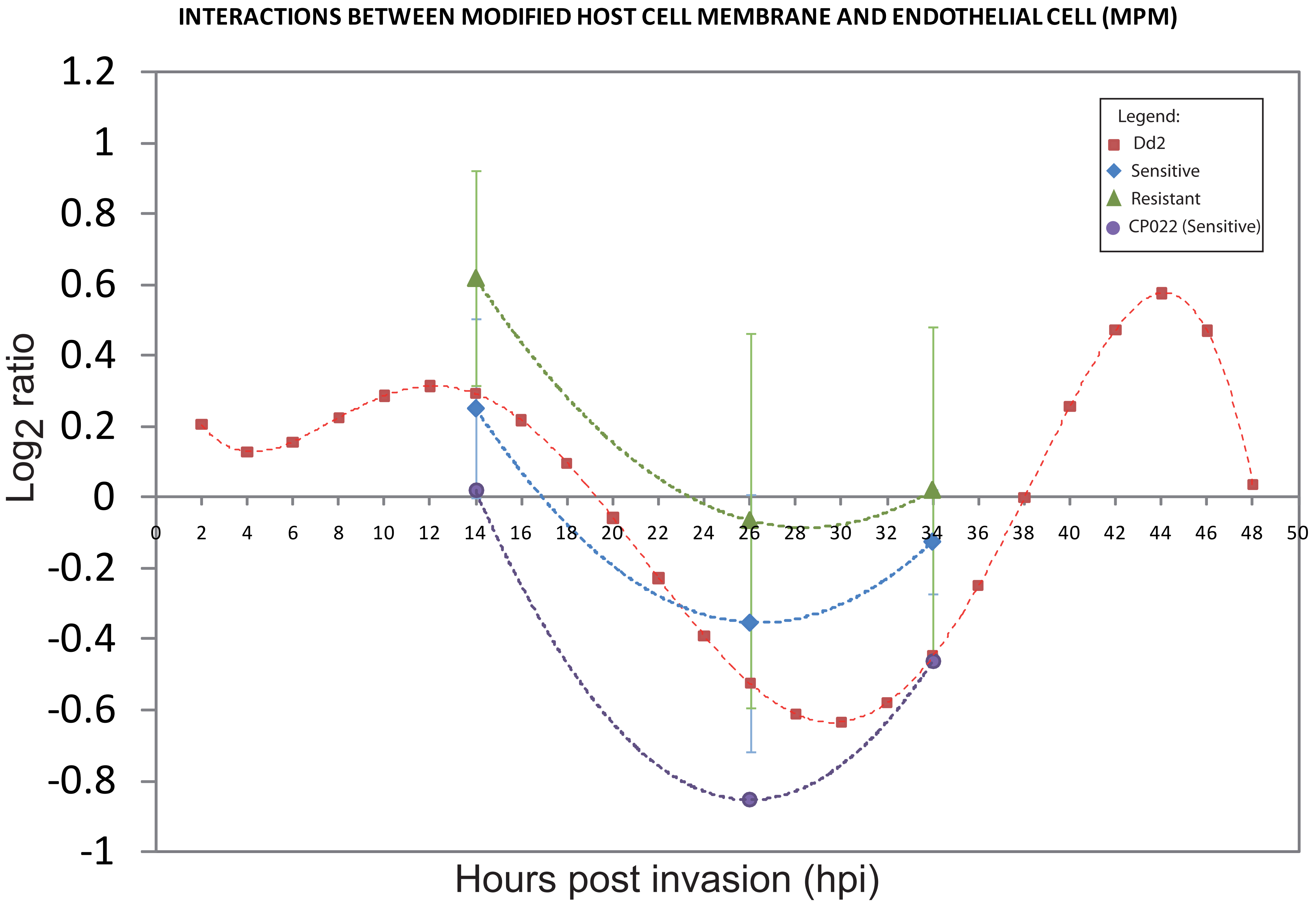
INTERACTIONS BETWEEN MODIFIED HOST CELL MEMBRANE AND ENDOTHELIAL CELL (MPM)
Legend:
Dd2
Sensitive
Resistant
CP022 (Sensitive)
Log2 ratio
1.2
1
0.8
0.6
0.4
0.2
0
-0.2
-0.4
-0.6
-0.8
-1
0 2 4 6 8 10 12 14 16 18 20 22 24 26 28 30 32 34 36 38 40 42 44 46 48 50
Hours post invasion (hpi)

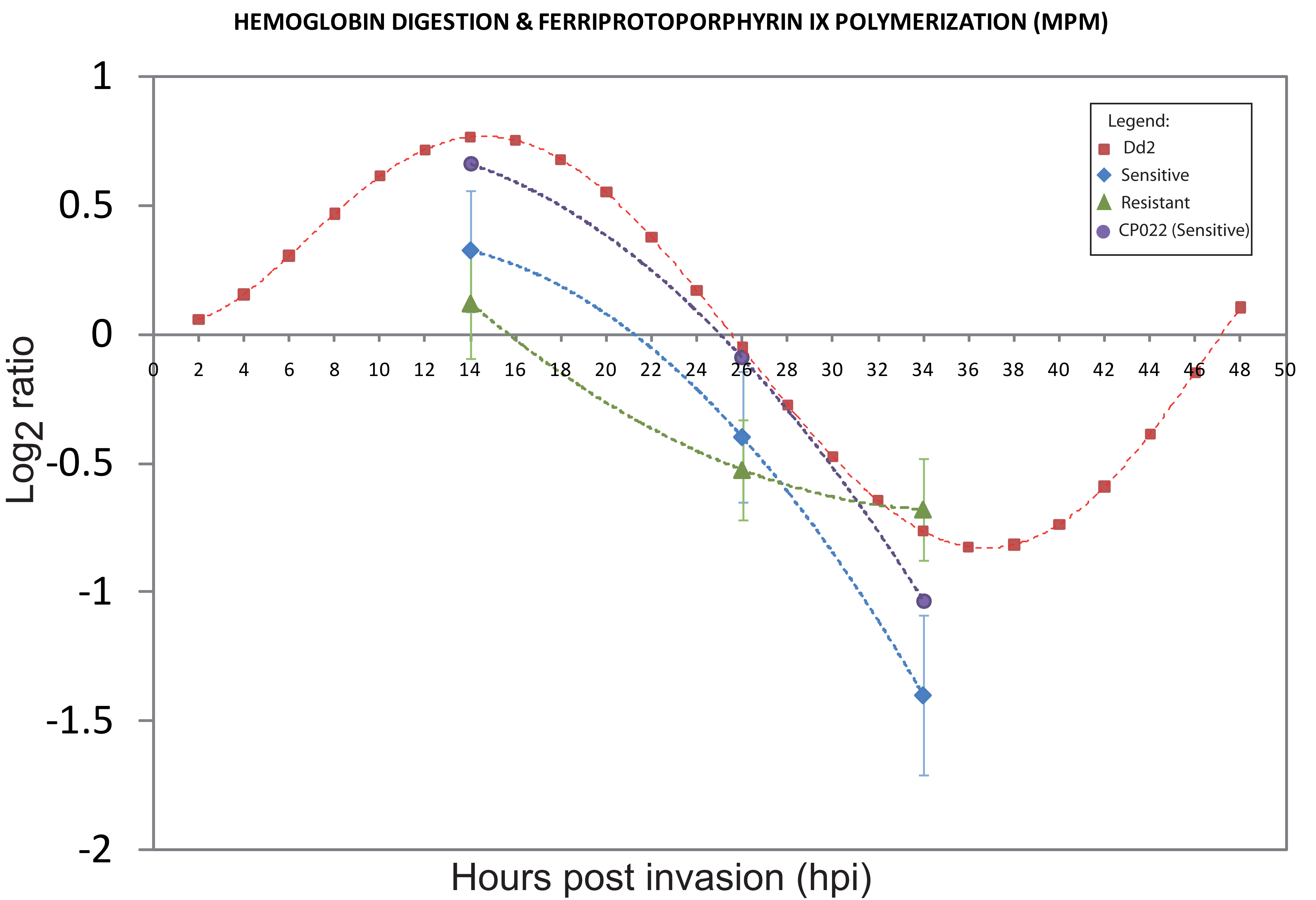
HEMOGLOBIN DIGESTION & FERRIPROTOPORPHYRIN IX POLYMERIZATION (MPM)
Legend:
Dd2
Sensitive
Resistant
CP022 (Sensitive)
Log2 ratio
1
0.5
0
-0.5
-1
-1.5
-2
0
2
4
6
8
10
12
14
16
18
20
22
24
26
28
30
32
34
36
38
40
42
44
46
48
50
Hours post invasion (hpi)

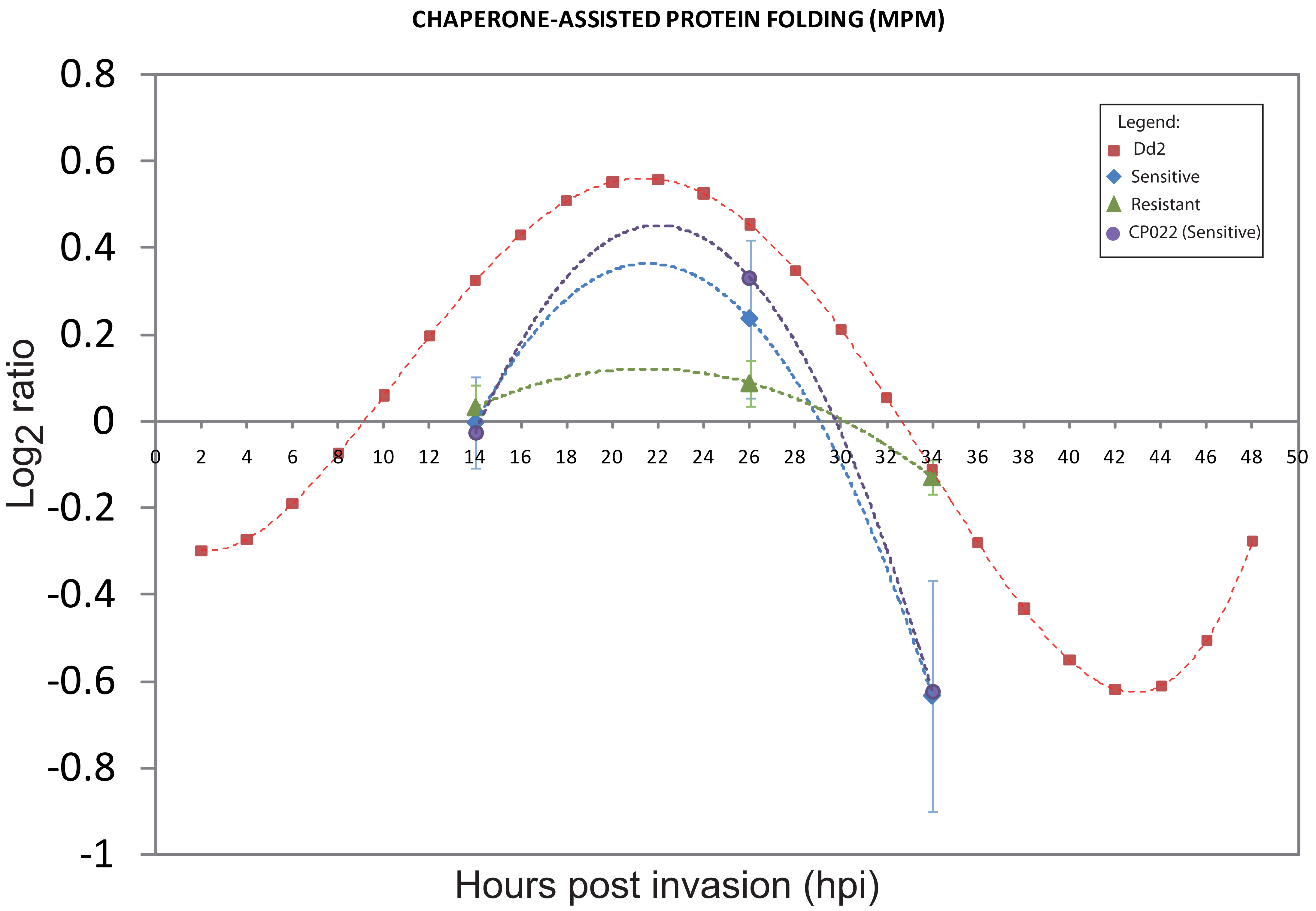

CHAPERONE-ASSISTED PROTEIN FOLDING (MPM)
Legend:
Dd2
Sensitive
Resistant
CP022 (Sensitive)
0.8
0.6
0.4
0.2
0
-0.2
-0.4
-0.6
-0.8
-1
Log2 ratio
0 2 4 6 8 10 12 14 16 18 20 22 24 26 28 30 32 34 36 38 40 42 44 46 48 50
Hours post invasion (hpi)

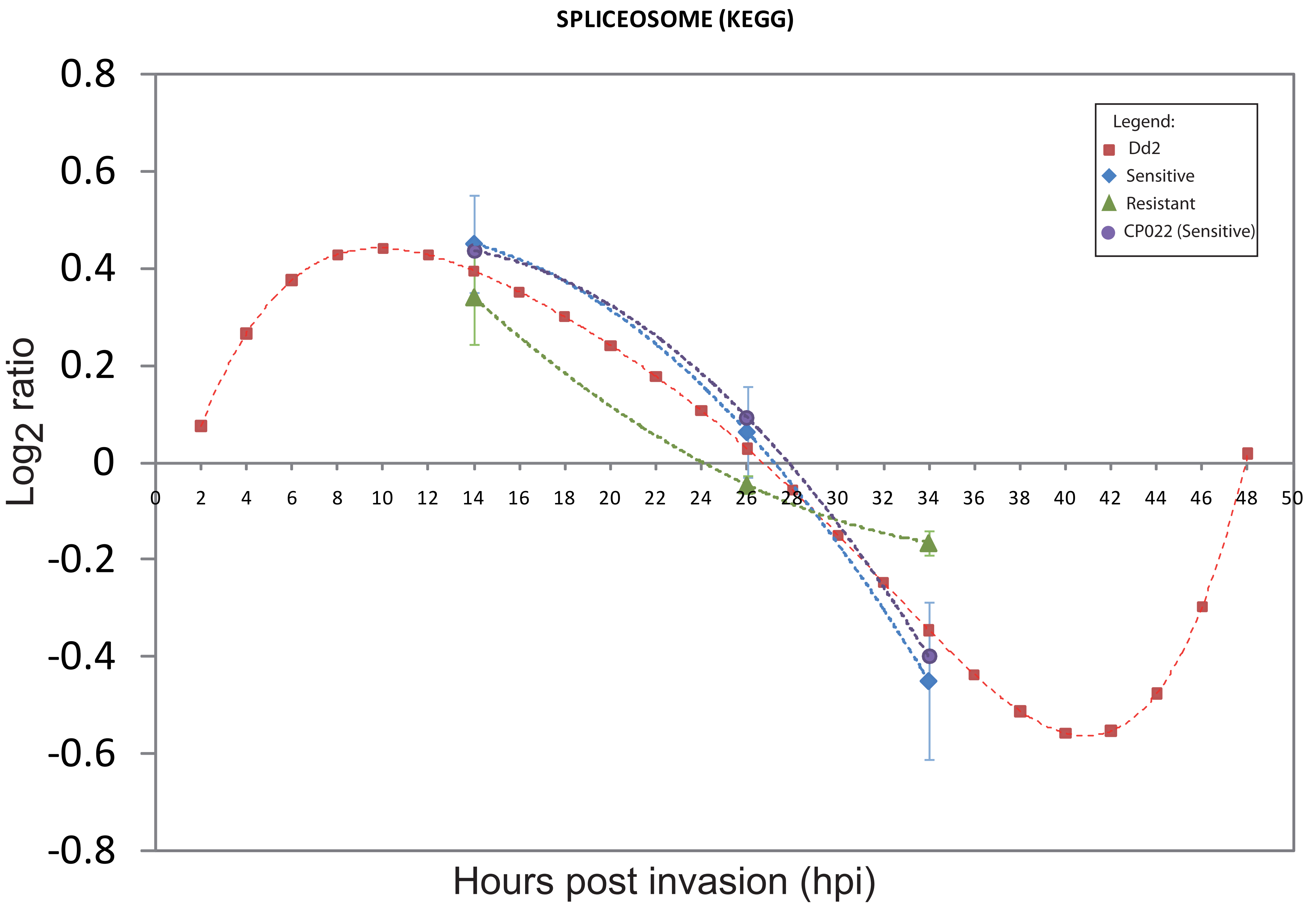
SPLICEOSOME (KEGG)
Legend:
Dd2
Sensitive
Resistant
CP022 (Sensitive)
0.8
0.6
0.4
0.2
0
-0.2
-0.4
-0.6
-0.8
Log2 ratio
0 2 4 6 8 10 12 14 16 18 20 22 24 26 28 30 32 34 36 38 40 42 44 46 48 50
Hours post invasion (hpi)

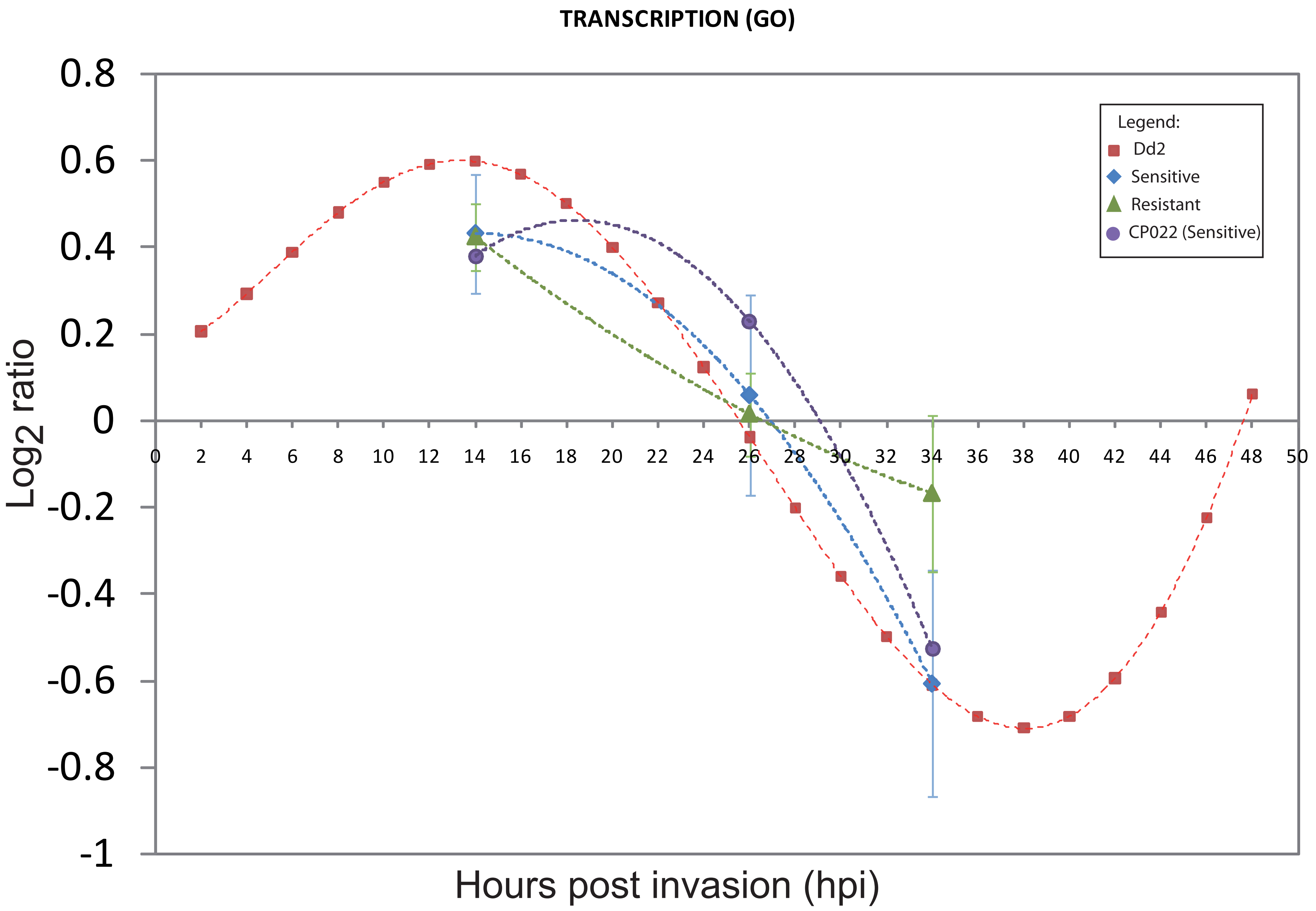
TRANSCRIPTION (GO)
Legend:
Dd2
Sensitive
Resistant
CP022 (Sensitive)
Log2 ratio
0.8
0.6
0.4
0.2
0
-0.2
-0.4
-0.6
-0.8
-1
0 2 4 6 8 10 12 14 16 18 20 22 24 26 28 30 32 34 36 38 40 42 44 46 48 50
Hours post invasion (hpi)

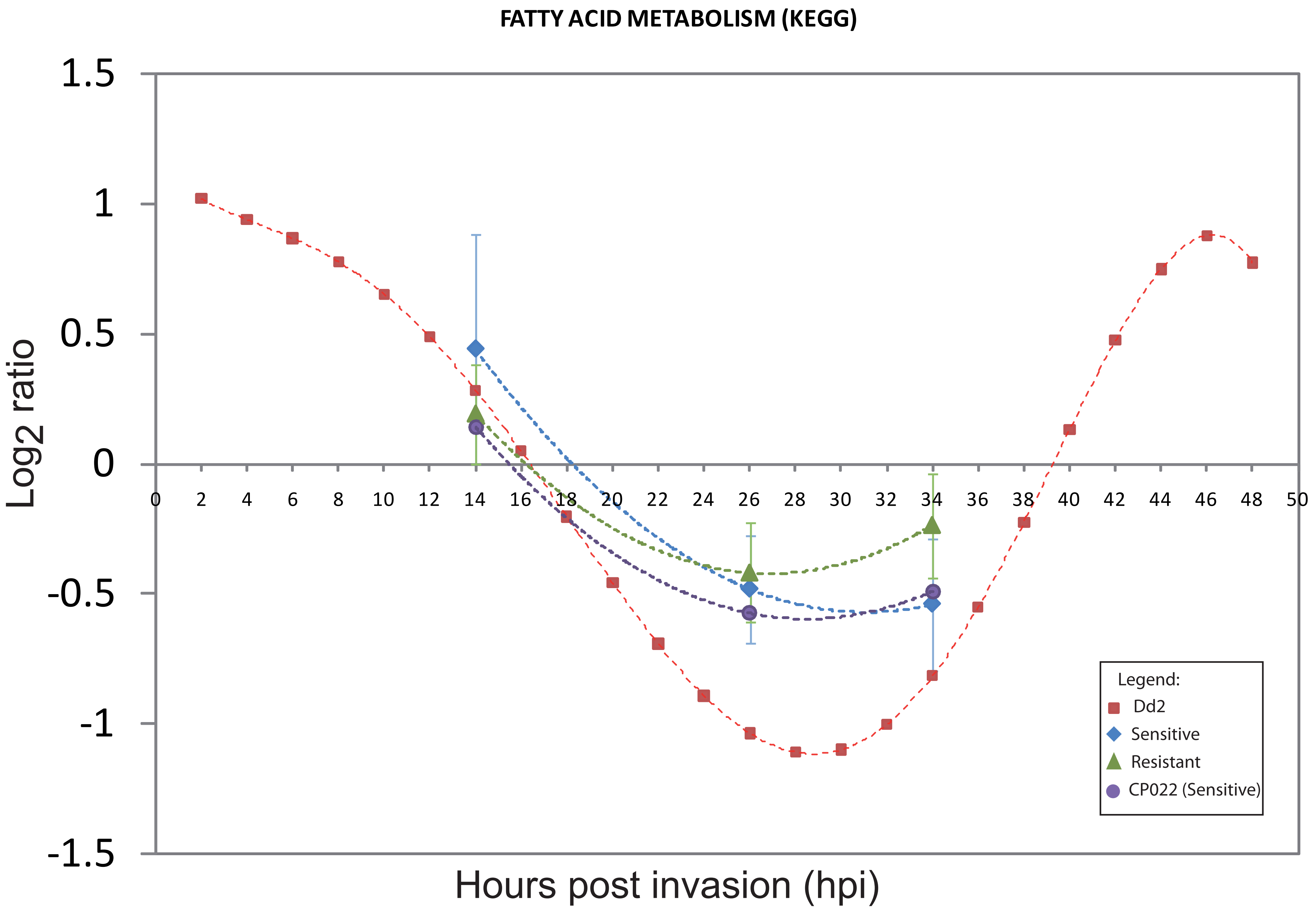
FATTY ACID METABOLISM (KEGG)
1.5
1
0.5
0
-0.5
-1
-1.5
Log2 ratio
0 2 4 6 8 10 12 14 16 18 20 22 24 26 28 30 32 34 36 38 40 42 44 46 48 50
Hours post invasion (hpi)
Legend:
Dd2
Sensitive
Resistant
CP022 (Sensitive)
