# Supplementary material for: Artemisinin resistance in Plasmodium falciparum is associated with an altered temporal pattern of transcription
Source: BMC Genomics. 2011 Aug 3;12:391. doi: 10.1186/1471-2164-12-391 (PMC3163569; doi:10.1186/1471-2164-12-391)
Supplement: Additional file 8 — Relative expression of individual genes associated with four functional pathways that have significant differential expression in artemisinin resistant parasite. Top: Example of four functional gene sets with significant differential expression between resistant and sensitive parasites and the relative gene expression of all members of the gene sets at the three stages. Each set of data points are the average log2 expression ratios of the isolates in a group and averaged for all the genes in that pathway at 14, 26 and 34 hpi. The curves are the best fit polynomial curves to the data points. Bottom: Each of the 3 graphs plotted depict the average log2 gene expression ratios of the isolates in the resistant (green triangle) or sensitive (blue diamond) group with the standard deviation represented by error bars in each particular pathway at14, 26 or 34 hpi. [file 1471-2164-12-391-S8.PDF]

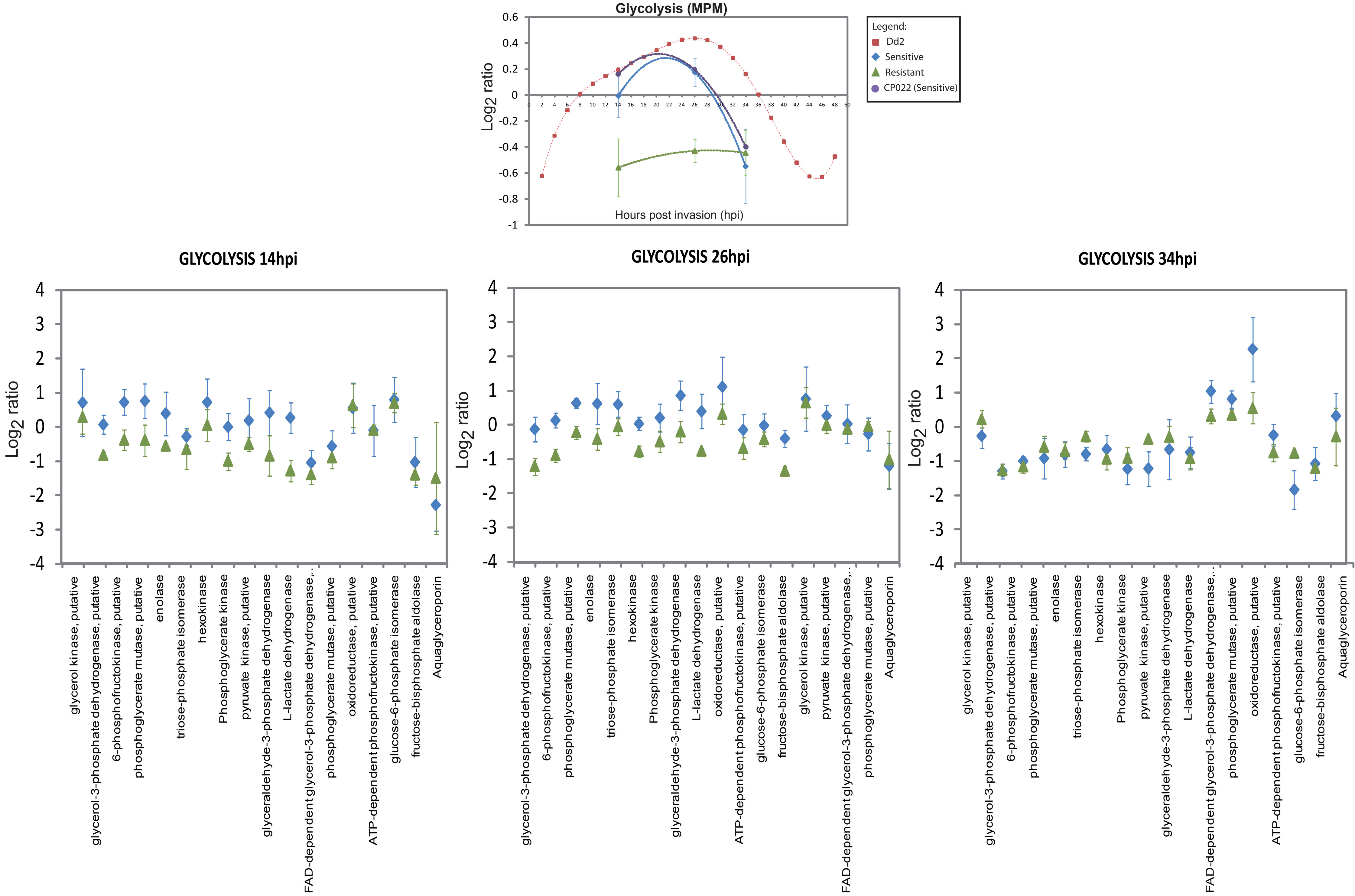

Glycolysis (MPM)
Log2 ratio
Hours post invasion (hpi)
Legend:
Dd2
Sensitive
Resistant
CP022 (Sensitive)
GLYCOLYSIS 14hpi
GLYCOLYSIS 26hpi
GLYCOLYSIS 34hpi
Log2 ratio
glycerol kinase, putative
glycerol-3-phosphate dehydrogenase, putative
6-phosphofructokinase, putative
phosphoglycerate mutase, putative
enolase
triose-phosphate isomerase
hexokinase
Phosphoglycerate kinase
pyruvate kinase, putative
glyceraldehyde-3-phosphate dehydrogenase
L-lactate dehydrogenase
FAD-dependent glycerol-3-phosphate dehydrogenase,...
phosphoglycerate mutase, putative
oxidoreductase, putative
ATP-dependent phosphofructokinase, putative
glucose-6-phosphate isomerase
fructose-bisphosphate aldolase
Aquaglyceroporin

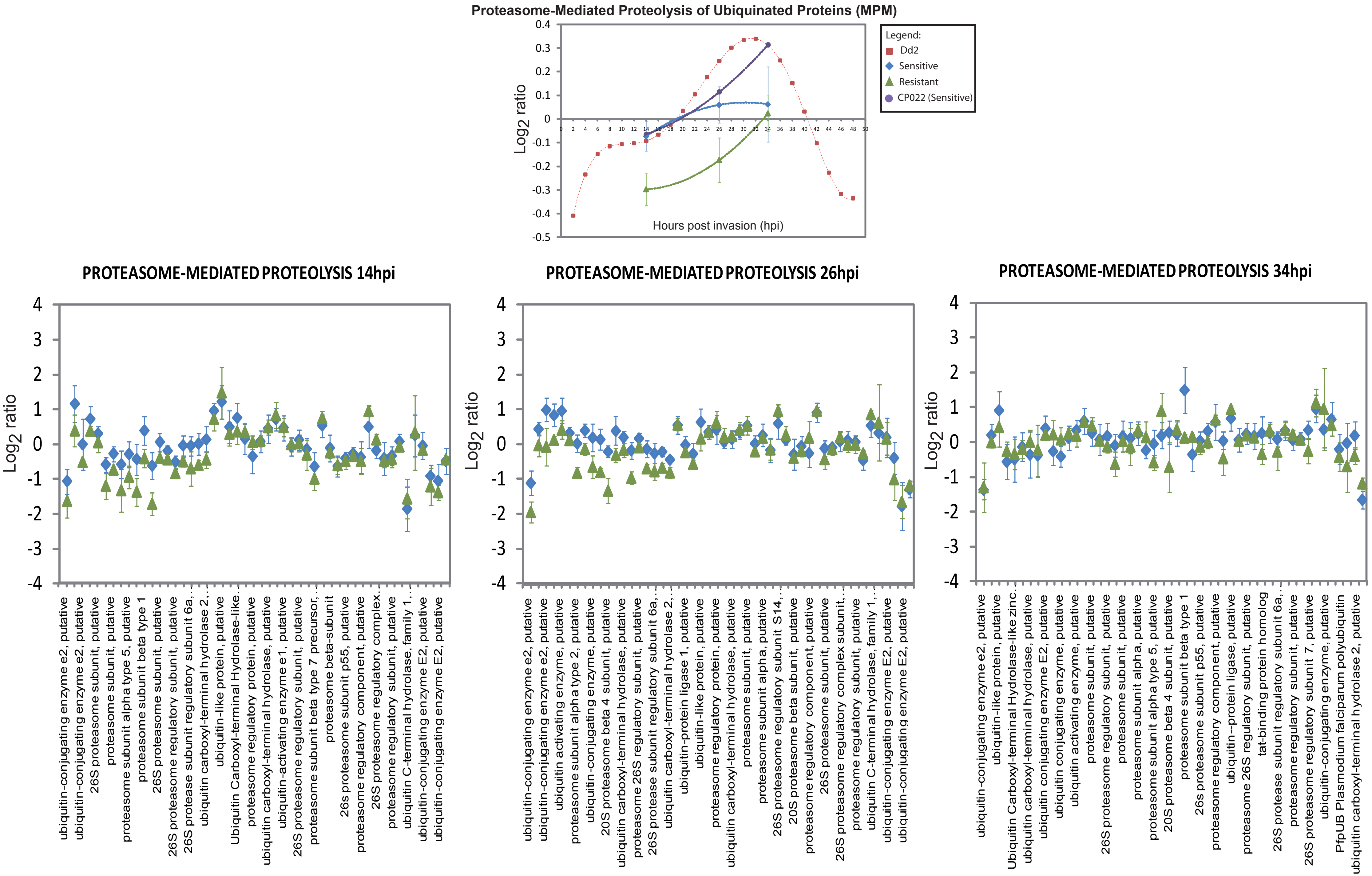
Proteasome-Mediated Proteolysis of Ubiquinated Proteins (MPM)
Log2 ratio
Hours post invasion (hpi)
Legend:
Dd2
Sensitive
Resistant
CP022 (Sensitive)
PROTEASOME-MEDIATED PROTEOLYSIS 14hpi
Log2 ratio
ubiquitin-conjugating enzyme e2, putative
ubiquitin-conjugating enzyme e2, putative
26S proteasome subunit, putative
proteasome subunit, putative
proteasome subunit alpha type 5, putative
proteasome subunit beta type 1
26S proteasome subunit, putative
26S proteasome regulatory subunit, putative
26S protease subunit regulatory subunit 6a,..
ubiquitin carboxyl-terminal hydrolase 2,..
ubiquitin-like protein, putative
Ubiquitin Carboxyl-terminal Hydrolase-like..
proteasome regulatory protein, putative
ubiquitin carboxyl-terminal hydrolase, putative
ubiquitin-activating enzyme e1, putative
26S proteasome regulatory subunit, putative
proteasome subunit beta type 7 precursor,..
proteasome beta-subunit
26s proteasome subunit p55, putative
proteasome regulatory component, putative
26S proteasome regulatory complex..
proteasome regulatory subunit, putative
ubiquitin C-terminal hydrolase, family 1,..
ubiquitin-conjugating enzyme E2, putative
ubiquitin-conjugating enzyme E2, putative
PROTEASOME-MEDIATED PROTEOLYSIS 26hpi
Log2 ratio
ubiquitin-conjugating enzyme e2, putative
ubiquitin-conjugating enzyme e2, putative
ubiquitin activating enzyme, putative
proteasome subunit alpha type 2, putative
ubiquitin-conjugating enzyme, putative
20S proteasome beta 4 subunit, putative
ubiquitin carboxyl-terminal hydrolase, putative
proteasome 26S regulatory subunit, putative
26S protease subunit regulatory subunit 6a,..
ubiquitin carboxyl-terminal hydrolase 2,..
ubiquitin-protein ligase 1, putative
ubiquitin-like protein, putative
proteasome regulatory protein, putative
ubiquitin carboxyl-terminal hydrolase, putative
proteasome subunit, putative
proteasome subunit alpha, putative
26S proteasome regulatory subunit S14,..
20S proteasome beta subunit, putative
proteasome regulatory component, putative
26S proteasome subunit, putative
26S proteasome regulatory complex subunit,..
proteasome regulatory subunit, putative
ubiquitin C-terminal hydrolase, family 1,..
ubiquitin-conjugating enzyme E2, putative
ubiquitin-conjugating enzyme E2, putative
PROTEASOME-MEDIATED PROTEOLYSIS 34hpi
Log2 ratio
ubiquitin-conjugating enzyme e2, putative
ubiquitin-like protein, putative
Ubiquitin Carboxyl-terminal Hydrolase-like zinc..
ubiquitin carboxyl-terminal hydrolase, putative
ubiquitin conjugating enzyme E2, putative
ubiquitin conjugating enzyme, putative
ubiquitin activating enzyme, putative
proteasome subunit, putative
26S proteasome regulatory subunit, putative
proteasome subunit, putative
proteasome subunit alpha, putative
proteasome subunit alpha type 5, putative
20S proteasome beta 4 subunit, putative
proteasome subunit beta type 1
26s proteasome subunit p55, putative
proteasome regulatory component, putative
ubiquitin--protein ligase, putative
proteasome 26S regulatory subunit, putative
tat-binding protein homolog
26S protease subunit regulatory subunit 6a,..
proteasome regulatory subunit, putative
26S proteasome regulatory subunit 7, putative
ubiquitin-conjugating enzyme, putative
PfUB Plasmodium falciparum polyubiquitin
ubiquitin carboxyl-terminal hydrolase 2, putative

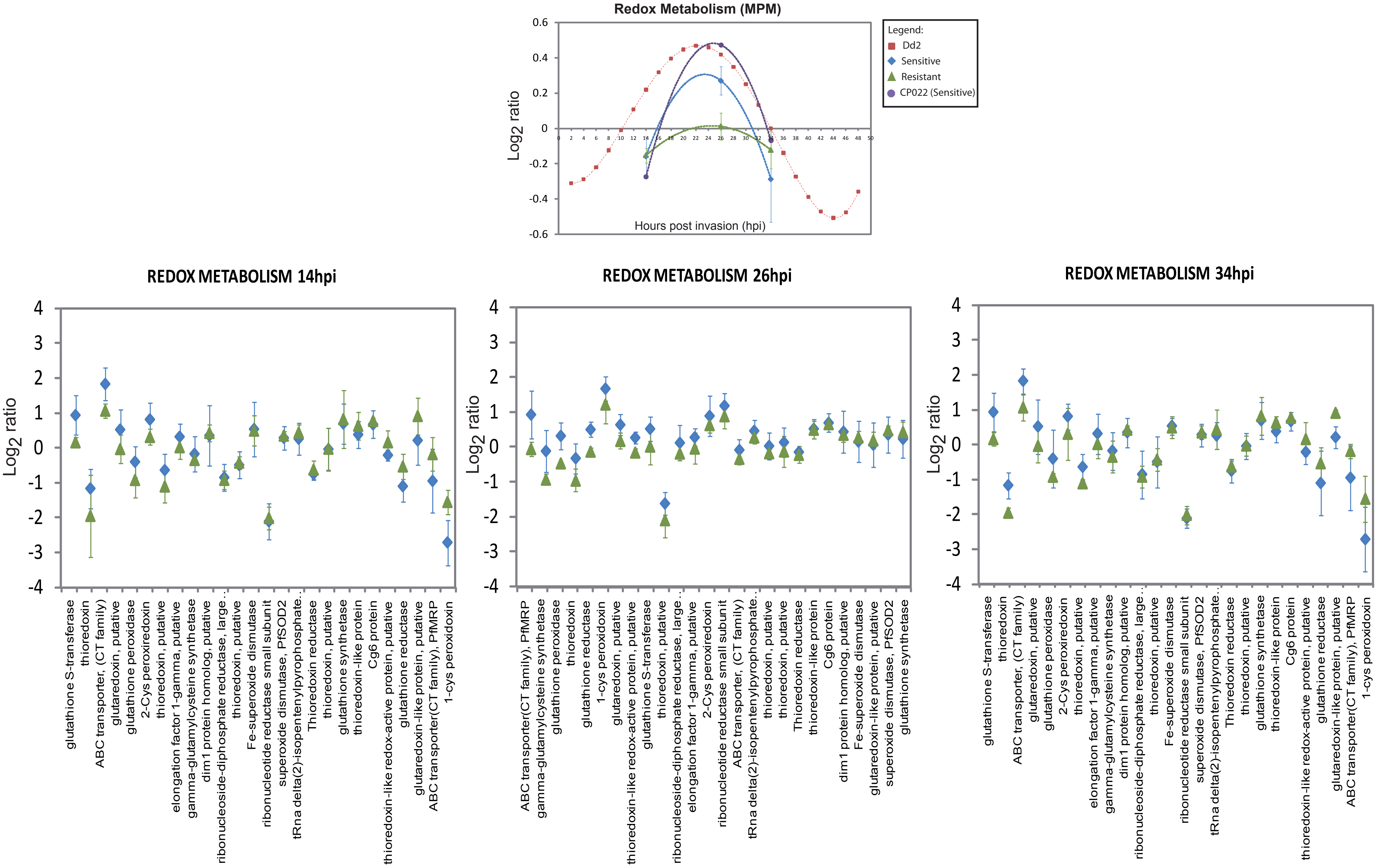

Redox Metabolism (MPM)
Legend:
Dd2
Sensitive
Resistant
CP022 (Sensitive)
Log2 ratio
Hours post invasion (hpi)
REDOX METABOLISM 14hpi
REDOX METABOLISM 26hpi
REDOX METABOLISM 34hpi
glutathione S-transferase
thioredoxin
ABC transporter, (CT family)
glutaredoxin, putative
glutathione peroxidase
2-Cys peroxiredoxin
thioredoxin, putative
elongation factor 1-gamma, putative
gamma-glutamylcysteine synthetase
dim1 protein homolog, putative
ribonucleoside-diphosphate reductase, large…
Fe-superoxide dismutase
ribonucleotide reductase small subunit
superoxide dismutase, PfSOD2
tRna delta(2)-isopentenylpyrophosphate…
Thioredoxin reductase
glutathione synthetase
thioredoxin-like protein
Cg6 protein
thioredoxin-like redox-active protein, putative
glutathione reductase
glutaredoxin-like protein, putative
ABC transporter(CT family), PfMRP
1-cys peroxidoxin
1-cys peroxidoxin

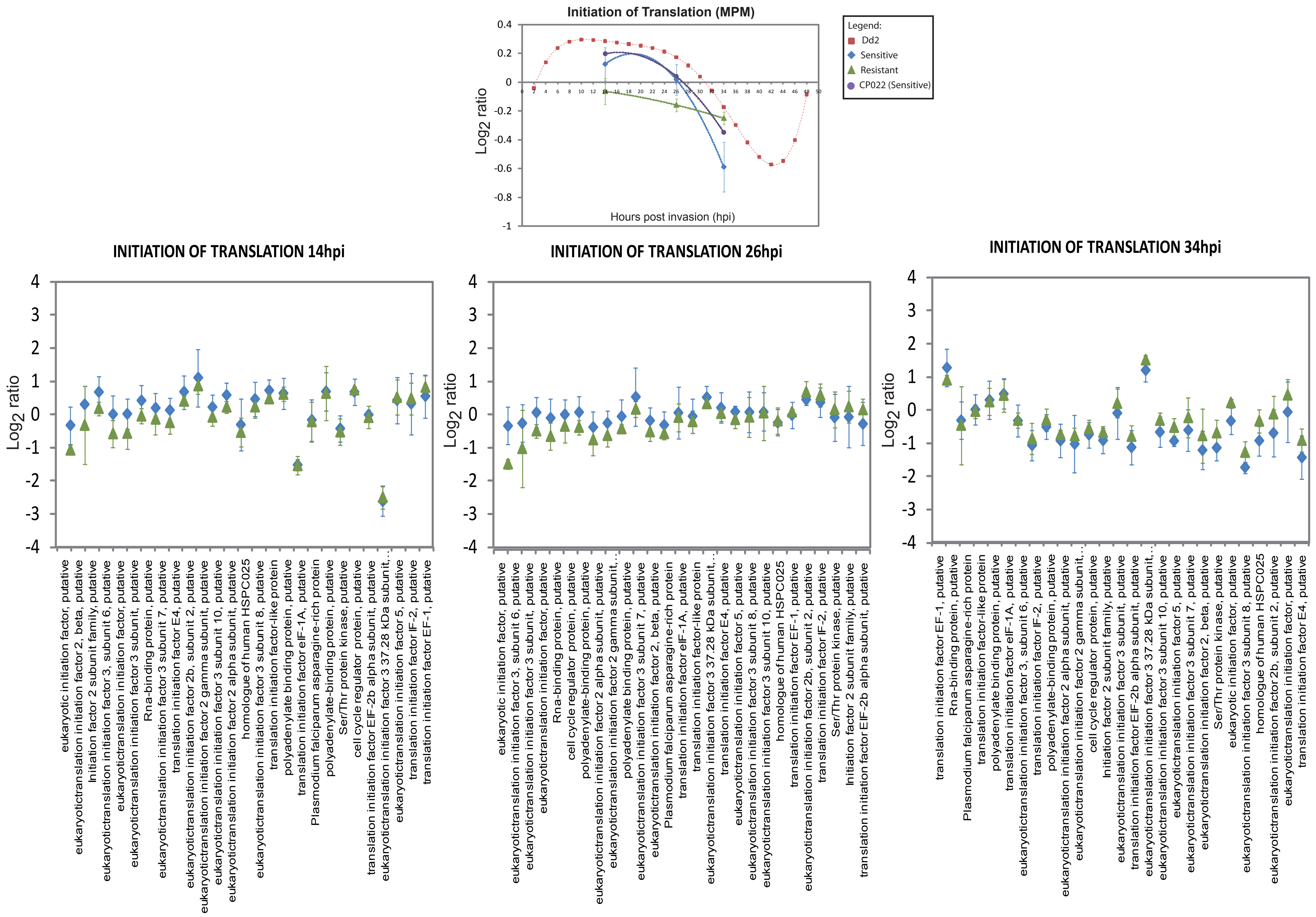

Initiation of Translation (MPM)
Legend:
Dd2
Sensitive
Resistant
CP022 (Sensitive)
Log2 ratio
Hours post invasion (hpi)
0.4
0.2
0
-0.2
-0.4
-0.6
-0.8
-1
0 2 4 6 8 10 12 14 16 18 20 22 24 26 28 30 32 34 36 38 40 42 44 46 48 50
INITIATION OF TRANSLATION 14hpi
INITIATION OF TRANSLATION 26hpi
INITIATION OF TRANSLATION 34hpi
Log2 ratio
4
3
2
1
0
-1
-2
-3
-4
eukaryotic initiation factor, putative
eukaryotictranslation initiation factor 2, beta, putative
Initiation factor 2 subunit family, putative
eukaryotictranslation initiation factor 3, subunit 6, putative
eukaryotictranslation initiation factor, putative
eukaryotictranslation initiation factor 3 subunit, putative
Rna-binding protein, putative
eukaryotictranslation initiation factor 3 subunit 7, putative
translation initiation factor E4, putative
eukaryotictranslation initiation factor 2b, subunit 2, putative
eukaryotictranslation initiation factor 2 gamma subunit, putative
eukaryotictranslation initiation factor 3 subunit 10, putative
eukaryotictranslation initiation factor 2 alpha subunit, putative
homologue of human HSPC025
eukaryotictranslation initiation factor 3 subunit 8, putative
translation initiation factor-like protein
polyadenylate binding protein, putative
translation initiation factor eIF-1A, putative
Plasmodium falciparum asparagine-rich protein
polyadenylate-binding protein, putative
Ser/Thr protein kinase, putative
cell cycle regulator protein, putative
translation initiation factor EIF-2b alpha subunit, putative
eukaryotictranslation initiation factor 3 37.28 kDa subunit,...
eukaryotictranslation initiation factor 5, putative
translation initiation factor IF-2, putative
translation initiation factor EF-1, putative
eukaryotic initiation factor, putative
eukaryotictranslation initiation factor 3, subunit 6, putative
eukaryotictranslation initiation factor 3 subunit, putative
eukaryotictranslation initiation factor, putative
Rna-binding protein, putative
cell cycle regulator protein, putative
polyadenylate-binding protein, putative
eukaryotictranslation initiation factor 2 alpha subunit, putative
eukaryotictranslation initiation factor 2 gamma subunit,...
polyadenylate binding protein, putative
eukaryotictranslation initiation factor 3 subunit 7, putative
eukaryotictranslation initiation factor 2, beta, putative
Plasmodium falciparum asparagine-rich protein
translation initiation factor eIF-1A, putative
translation initiation factor-like protein
eukaryotictranslation initiation factor 3 37.28 kDa subunit,...
translation initiation factor E4, putative
eukaryotictranslation initiation factor 5, putative
eukaryotictranslation initiation factor 3 subunit 8, putative
eukaryotictranslation initiation factor 3 subunit 10, putative
homologue of human HSPC025
translation initiation factor EF-1, putative
eukaryotictranslation initiation factor 2b, subunit 2, putative
translation initiation factor IF-2, putative
Ser/Thr protein kinase, putative
Initiation factor 2 subunit family, putative
translation initiation factor EIF-2b alpha subunit, putative
translation initiation factor EF-1, putative
Rna-binding protein, putative
Plasmodium falciparum asparagine-rich protein
translation initiation factor-like protein
polyadenylate binding protein, putative
translation initiation factor eIF-1A, putative
eukaryotictranslation initiation factor 3, subunit 6, putative
translation initiation factor IF-2, putative
polyadenylate-binding protein, putative
eukaryotictranslation initiation factor 2 alpha subunit, putative
eukaryotictranslation initiation factor 2 gamma subunit,...
cell cycle regulator protein, putative
Initiation factor 2 subunit family, putative
eukaryotictranslation initiation factor 3 subunit, putative
translation initiation factor EIF-2b alpha subunit, putative
eukaryotictranslation initiation factor 3 37.28 kDa subunit,...
eukaryotictranslation initiation factor 3 subunit 10, putative
eukaryotictranslation initiation factor 5, putative
eukaryotictranslation initiation factor 3 subunit 7, putative
eukaryotictranslation initiation factor 2, beta, putative
Ser/Thr protein kinase, putative
eukaryotic initiation factor, putative
eukaryotictranslation initiation factor 3 subunit 8, putative
homologue of human HSPC025
eukaryotictranslation initiation factor 2b, subunit 2, putative
eukaryotictranslation initiation factor, putative
translation initiation factor E4, putative
