# Supplementary material for: Artemisinin resistance in Plasmodium falciparum is associated with an altered temporal pattern of transcription
Source: BMC Genomics. 2011 Aug 3;12:391. doi: 10.1186/1471-2164-12-391 (PMC3163569; doi:10.1186/1471-2164-12-391)
Supplement: Additional file 9 — Transcriptional profiles of the 3 genes in the artemisinin resistant parasites compared with previous data published [18]. The mean expression log2 ratio (data point) and SD (error bars) among the resistant (green triangle) and sensitive (blue diamond) isolates for each stage are plotted for the genes: PF10_0121 - hypoxanthine phosphoribosyltransferase (rank 3207/4029; p-value = 0.1), PF08_0054 - Heat Shock Protein 70 kDa (rank 14/4041; p-value = 0.0004) and PFE1415w- cell cycle regulator (rank 3837/4041; p-value = 0.01). The polynomial represents the best fit curve through the data points. The arrow indicates the approximate stage in which significant increased or lowered expression was observed in artesunate-tolerant parasites and the fold change [18] (Witkowski et al., 2010). [file 1471-2164-12-391-S9.PDF]

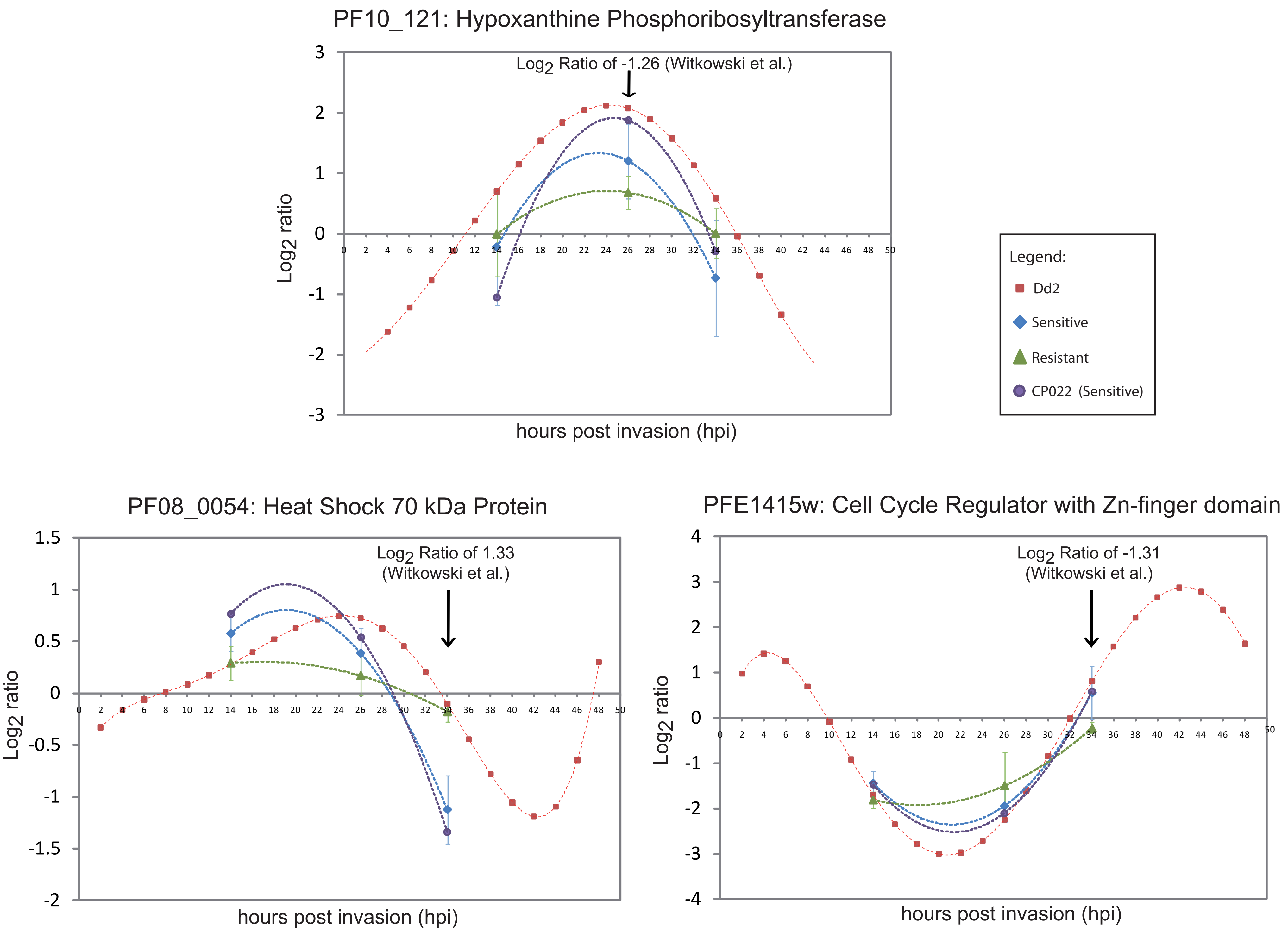

PF10_121: Hypoxanthine Phosphoribosyltransferase
Log2 Ratio of -1.26 (Witkowski et al.)
Log2 ratio
hours post invasion (hpi)
Legend:
Dd2
Sensitive
Resistant
CP022 (Sensitive)
PF08_0054: Heat Shock 70 kDa Protein
Log2 Ratio of 1.33
(Witkowski et al.)
Log2 ratio
hours post invasion (hpi)
PFE1415w: Cell Cycle Regulator with Zn-finger domain
Log2 Ratio of -1.31
(Witkowski et al.)
Log2 ratio
hours post invasion (hpi)
